# Evolution of Artificial Arginine Analogues—Fluorescent Guanidiniocarbonyl-Indoles as Efficient Oxo-Anion Binders

**DOI:** 10.3390/molecules27093005

**Published:** 2022-05-07

**Authors:** Daniel Sebena, Kevin Rudolph, Bibhisan Roy, Christoph Wölper, Till Nitschke, Sarah Lampe, Michael Giese, Jens Voskuhl

**Affiliations:** 1 Faculty of Chemistry (Organic Chemistry), University of Duisburg-Essen, 45117 Essen, Germany; daniel.sebena@uni-due.de (D.S.); kevin.rudolph@uni-due.de (K.R.); bibhisan.roy@uni-due.de (B.R.); till.nitschke@stud.uni-due.de (T.N.); sarah.lampe89@web.de (S.L.); 2 Faculty of Chemistry (Inorganic Chemistry), University of Duisburg-Essen, 45117 Essen, Germany; christoph.woelper@uni-due.de

**Keywords:** oxo-anion binders, molecular recognition, fluorescence, supramolecular chemistry

## Abstract

In this article, we present fluorescent guanidiniocarbonyl-indoles as versatile oxo-anion binders. Herein, the guanidiniocarbonyl-indole (GCI) and methoxy-guanidiniocarbonyl-indole (MGCI) were investigated as ethylamides and compared with the well-known guanidiniocarbonyl-pyrrole (GCP) concerning their photophysical properties as well as their binding behavior towards oxo-anions. Hence, a variety of anionic species, such as carboxylates, phosphonates and sulfonates, have been studied regarding their binding properties with GCP, GCI and MGCI using UV-Vis titrations, in combination with the determination of the complex stoichiometry using the Job method. The emission properties were studied in relation to the pH value using fluorescence spectroscopy as well as the determination of the photoluminescence quantum yields (PLQY). Density functional theory (DFT) calculations were undertaken to obtain a better understanding of the ground-lying electronic properties of the investigated oxo-anion binders. Additionally, X-ray diffraction of GCP and GCI was conducted. We found that GCI and MGCI efficiently bind carboxylates, phosphonates and sulfonates in buffered aqueous solution and in a similar range as GCP (K_ass_ ≈ 1000–18,000 M^−1^, in bis-tris buffer, pH = 6); thus, they could be regarded as promising emissive oxo-anion binders. They also exhibit a visible fluorescence with a sufficient PLQY. Additionally, the excitation and emission wavelength of MGCI was successfully shifted closer to the visible region of the electromagnetic spectrum by introducing a methoxy-group into the core structure, which makes them interesting for biological applications.

## 1. Introduction

The molecular recognition of oxo-anions in water is one of the key challenges in today’s supramolecular chemistry, as for applications in biological, medicinal or environmental fields [1,2], a high binding in aqueous media as well as significant specificity towards the desired target is needed. However, the design of synthetic anion receptors for this purpose remains challenging [2]. Despite the difficulty to synthesise anion receptors, which are soluble in water, water itself is a highly competitive polar solvent, able to hydrate host and guest through hydrogen-bond donation and acceptance [2]. Additionally, anions themselves possess properties which make them difficult to bind in aqueous solutions; in contrast to cations of the same charge and size, they are more heavily solvated in water and quite large, leading to weaker electrostatic interactions with a positive charged binding partner [2].

**Figure 1:**
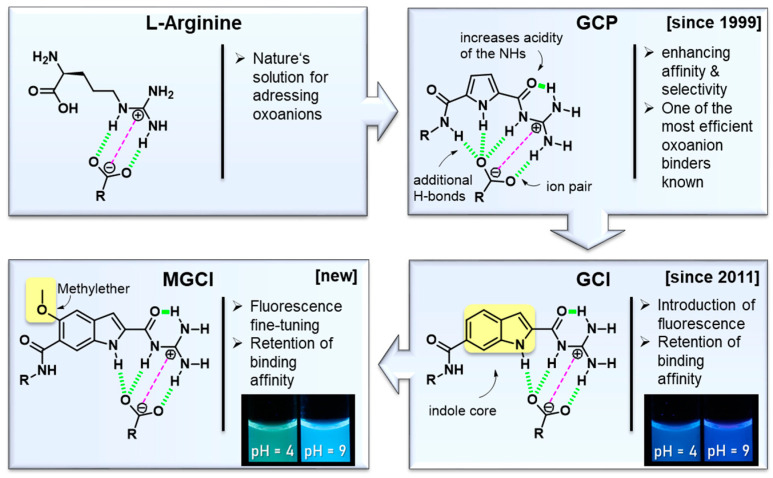
Schematic representation of the evolution of artificial arginine analogues from guanidinocarbonyl-pyrrole (GCP) to guanidinocarbonyl-indole (GCI) and methoxy-guanidinocarbonyl-indole (MGCI).

Therefore, it is prudent to consider how nature solves this problem, which is often by utilising the guanidinium-cation moiety. It remains protonated at physiological pH and is able to form not only electrostatic interactions but also two strong parallel H-bonds, which makes it particularly advantageous for the binding of oxo-anions such as carboxylates and phosphates [2,3] (Figure 1).

The guanidinium unit is naturally present in the proteogenic amino acid arginine, which plays as crucial role in the active site of many enzymes [4], such as lactate dehydroxygenase, by binding anionic substrates [5,6]. Based on its essential role in nature, the guanidinium group was widely used in the supramolecular community for decades for the development of anion receptors [7,8,9,10,11,12]. There are numerous examples of compounds that can bind to oxo-anions, such as carboxylates [10,13,14,15,16], but few that retain strong complex affinities in aqueous solvents [2,17,18,19,20,21], due to the difficulties in addressing oxo-anions in water, as mentioned above. One early example was discovered by Anslyn et al., who were able to bind phosphodiesters with a bisguanidin cleft in DMSO/water mixtures [17]. More recently, Winter et al. were able to bind carboxylates, with a focus on dicarboxylates, with ferrocenes, functionalised with acetlyguanidine, in pure water [21].

In 1999, Schmuck et al. introduced the guanidiniocarbonyl pyrrole cation (GCP) [22,23] as novel efficient oxo-anion-binder in polar, aqueous media. The guanidinium is connected to the pyrrole at the 2-position via a carbonyl linker, which leads, due to an additional intramolecular H-bond, to a conformational fixation as well as an increased acidity of the guanidinium group.

Additionally, GCP forms a hydrogen bond network consisting of four H-bonds, which, together with ionic interactions, leads to a strong binding, even in aqueous media [24] (Figure 1). Another feature of GCP is the possibility of modifying the binding unit at the amide terminus. These variations allow for new interactions, which enables the binding selectivity to be tuned for specific anionic substrates [24]. GCP revealed not only excellent association constants for carboxylates (K_a_ = 2800 M^−1^ for acetate in DMSO:water = 60:40 at neutral pH) [22] but also for phosphates (a tweezer receptor containing two GCP moieties binds different phosphates with binding constants between 10^4^ M^−1^ and 10^5^ M^−1^ in buffered water, pH = 7, but no single GCP was investigated regarding its phosphate binding) [25], which opens up various possible applications. Therefore, the GCP moiety was introduced in various structures, which facilitated a multitude of applications, ranging from sensing [25] to material science aspects [26]. In this context, its use in biomedical applications is especially noteworthy. Here, two strategies were predominantly explored. The first strategy was to address the carboxylates (Asp, Glu) of protein surfaces by non-covalent interactions, intending to modulate protein functions [27,28]. The second strategy involved using GCP-containing molecules as transfection vectors [29] for gene delivery, which is possible due to the remarkably strong interaction with the phosphate backbone of DNA [30]. A comprehensive summary of GCP studies can be found in recent reviews [24,31]. Structures including indole cores were described by Schmuck et al. more than 10 years ago [32]. The main focus of this investigation was the exploration of several different indole structures as oxo-anion binders. It was shown that indoles functionalised with acetylguanidinium cations (similar to GCP) can bind a carboxylate efficiently in organic solvent (K_a_ = 10^4^–10^5^ M^−1^ to *N*-acetylated aniline in DMSO). In the present contribution, we focus on the emissive behaviour of the GCI-based oxo-anion binders, since they enable tracking inside a biological environment, which will lead to numerous applications in the field of biomedical research.

## 2. Results and Discussion

**Figure 2:**
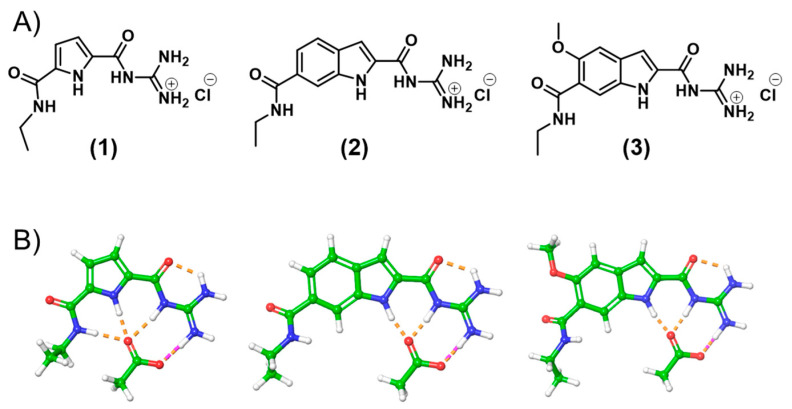
(**A**) Molecular structures of GCP ethyl amide (**1**), GCI ethyl amide (**2**), MGCI ethyl amide (**3**). (**B**) calculated lowest energy structure of complexes of (**1**), (**2**) and (**3**) with acetate anions, force field calculations (MacroModel V12.4, OPLS 2005 force field, conformational search, in water, mixed torsional/low-mode sampling). Ionic interactions are depicted in pink, H-bonds in orange.

The present investigation reports two emissive oxo-anion binders, namely guanidinocarbonyl indole (GCI), a structure previously described by us but not investigated concerning its fluorescence behaviour or its oxo-anion affinity in aqueous solutions [32] and the advanced methoxy-guanidiniocarbonyl-indole (MGCI), featuring the desired emission fine tuning properties, while retaining its oxo-anion binding affinity in aqueous media (Figure 2).

MGCI features a methoxy group at the C-5 position of the indole moiety, leading to a red shift of excitation and emission wavelength, which is known for indoles. This red shift is significant, as GCI’s excitation is in the UV range and is therefore troublesome for some applications, such as bioimaging, which leads to UV damage of the corresponding biological samples of interest. It will certainly be beneficial to combine emission properties with oxo-anion binding properties [33].

The synthesis of (**1**) and (**2**) were previously described by us [22,34]. Both (**2**) and (**3**) were synthesised in a four-step respective six-step synthesis route. Both syntheses include a Heck reaction [32], followed by the exchange of protective groups of the aromatic carboxylates from methyl to benzyl; a crucial step, as cleavage of the methyl ester leads to a decomposition of the guanidine-group. For the examination of the specific characteristics, the novel indole moieties as well as the known GCP moiety were functionalised as ethyl amides in two steps (**1**, **2** and **3**) (for detailed synthesis routes see Schemes S1 and S2, ESI). This is necessary because a free carboxylic acid in combination with a free guanidinium leads to strong self-assembly (K_dim_ > 10^2^ in water) [35]. The ethyl-amide was chosen as the model because the attachment of the additional functionalities typically occurs via amide formation at the free carboxylate. Additionally, this group should not have an influence on binding behaviour of the moieties itself. Even though the substitution of the pyrrole with an indole should clearly improve the photophysical properties of GCI (**2**) and MGCI (**3**), we were interested in the possible binding conformations to oxo-anions.

Therefore, we calculated the lowest energy structures for the binding motifs (**1**), (**2**) and (**3**) as complex with oxo-anions (carboxylates, phosphonates and sulfonates) in water as a solvent, to gain insight into the most likely binding mode, using a force-field based molecular modelling approach (MacroModel by Schrödinger, for details see chapter 8, ESI and Figure 2).

Hereby, we verified the expected and known binding conformation of (**1**) [23] with one ionic interaction and four H-bonds. Interestingly, for sulfonates, just three H-bonds were calculated. We expected a similar binding mode as for carboxylates and phosphonates. It seems that in this case the formation of a fourth H-bond to the sulfonate oxygen via the ethylamide NH is not favoured, as the weak energetic contribution of the additional H-bond in water is not enough to rotate the ethylamide group in the necessary direction. The novel binding moieties (**2**) and (**3**) form very similar complexes with the oxo-anions which are comparable with (**1**). The hydrogen bond network, therefore, is just formed by three H-bonds and not four due to the structural arrangement of the indole moiety. The lowest energy structure of the complexes with acetate are depicted in Figure 2B.

Encouraged by these findings, which indicate comparable binding affinities of the receptor moieties to anions, we proceeded with the development of the indole binding moieties. Both (**2**) and (**3**) were designed to be analogous to the GCP moiety with a free carboxylic acid enabling further functionalisation via common peptide coupling (for the syntheses routes, see the ESI).

**Figure 3:**
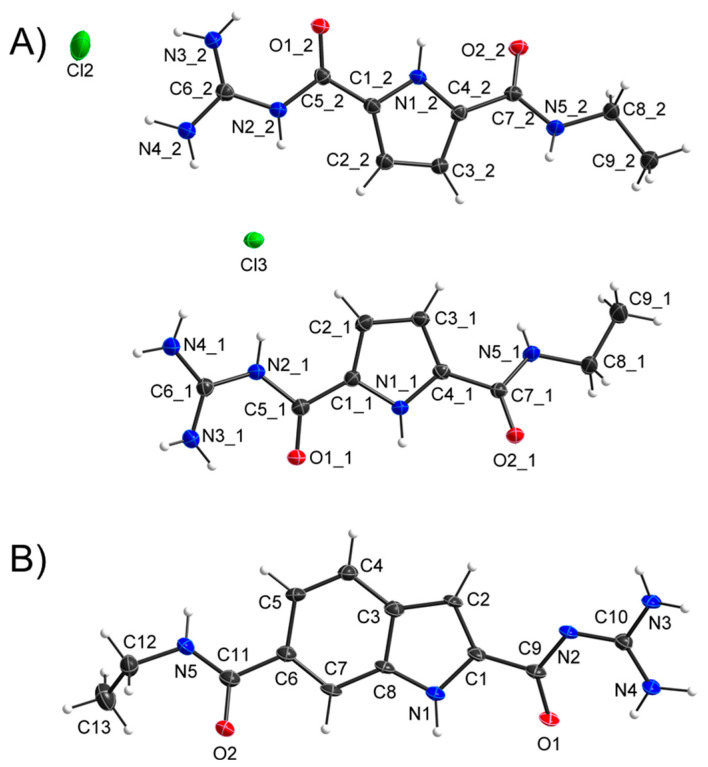
(**A**) X-ray diffraction analysis of GCP ethylamide (**1**) crystallised from methanol/water as cation. (**B**) X-ray diffraction analysis of GCI ethylamide (**2**) crystallised from water. Displacement ellipsoids are shown at 50% probability levels. Hydrogen atoms are displayed as spheres of arbitrary radius. Solvent molecules and counter-ions have been omitted for clarity (CCDC numbers: 2,150,514 and 2,150,515).

The structures of (**1**) and (**2**) were further confirmed by X-ray diffraction (Figure 3). Compound (**1**) was obtained in the cationic form (with Cl^−^ as counterion) and was found to crystallise in the triclinic space group *P*$\bar{1}$. The asymmetric unit comprises two independent ion pairs and two water molecules, of which one is disordered over two positions. The cations are flat with r.m.s. deviations from the best plane of the atoms of 0.18 Å and 0.21 Å, respectively, and O2 showing the largest offset from the plane (0.4830(18) Å, 0.5552(18) Å). The offset of O2 in both cations can easily be explained by the layered packing. The O2 atoms are the acceptors for hydrogen bonds that connect these layers and thus have to be oriented out of plane. Within the plane, centrosymmetric dimers can be found that are linked by R^2^_2_(10) motifs extended by an additional NH⋯O bond leading to a tricyclic system of hydrogen bonds. These dimers are connected by classical and non-classical hydrogen bonds to the anion and the water molecules.

Compound (**2**) was crystallised in its neutral form, featuring the monoclinic space group *C*2 with one independent molecule in the asymmetric unit accompanied by three water molecules. The delocalised electron systems of the side-arms are planar and slightly tilted towards the central indole moiety (angles between best planes: C(=O)N 19.2(7)°, C(=O)NC(NH_2_)_2_ 24.64(19)°). The ethyl-group is oriented approximately perpendicular to the molecular plane. Bond length and angles do not show any significant deviations from expected values; thus, we consider them realistic, despite their low reliability due to the weak scattering power of the crystal. Since the reliability of the donor hydrogen positions is rather low, we do not consider it appropriate to discuss hydrogen bonding in the packing. Unfortunately, no crystals of sufficient size and quality for the X-ray crystallography of (**3**) could be generated.

**Figure 4:**
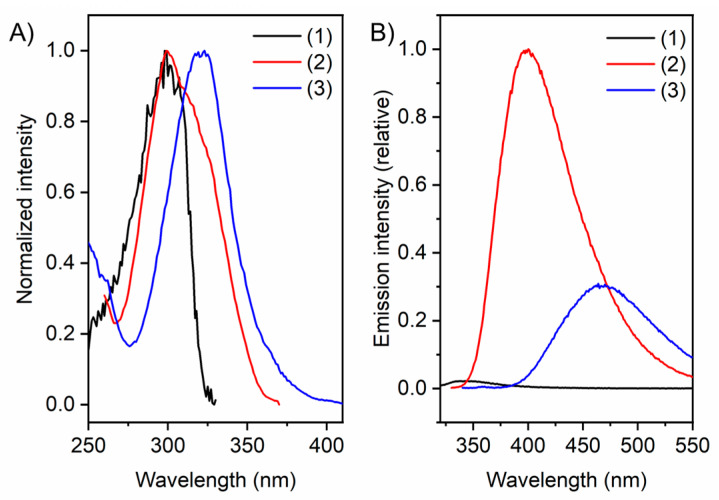
(**A**) Normalised excitation spectra of (**1**), (**2**) and (**3**). (**B**) Emission spectra of (**1**), (**2**) and (**3**) excited at corresponding excitation wavelength. Conditions: Concentration = 10 µM in bis-tris buffer 6 mM, pH = 6.

With these three compounds in hand, we proceeded to examine the fluorescence behaviour of (**2**) and (**3**). While the fluorescence of GCP (**1**) is negligible, (**2**) and (**3**) showed, as expected, a significant fluorescence emission. Comparing the samples in an aqueous 2-Bis(2-hydroxyethyl)amino2-(hydroxymethyl)-1,3-propandiole (bis-tris) buffer with a defined concentration and a pH of 6, the emission of (**3**) is significantly stronger than the emission of (**1**). However, the strongest fluorescence is observed for (**2**) and the emission maximum shifts bathochromically from 344 nm in the case of (**1**) to 400 nm for (**2**) and 468 nm for (**3**) (Figure 4B; Table 1). Compound (**3**) features a methoxy group at the C-5 position of the indole moiety, leading to a red shift of excitation and emission wavelength, which is known for indoles. This red shift is significant, as GCI’s excitation is in the UV range and therefore troublesome for some applications, such as bioimaging, leading to UV damage of the corresponding biological samples of interest.

To summarise, we observed a desired red shift of the excitation, from a narrow distribution with a peak at 299 nm for (**1**), going to a broader distribution with the same peak wavelength of 299 nm for (**2**), and finally going to a broad distribution with a peak at 321 nm for (**3**) (Figure 4A; Table 1), featuring a signal even in the range of 380–400.

**Figure 5:**
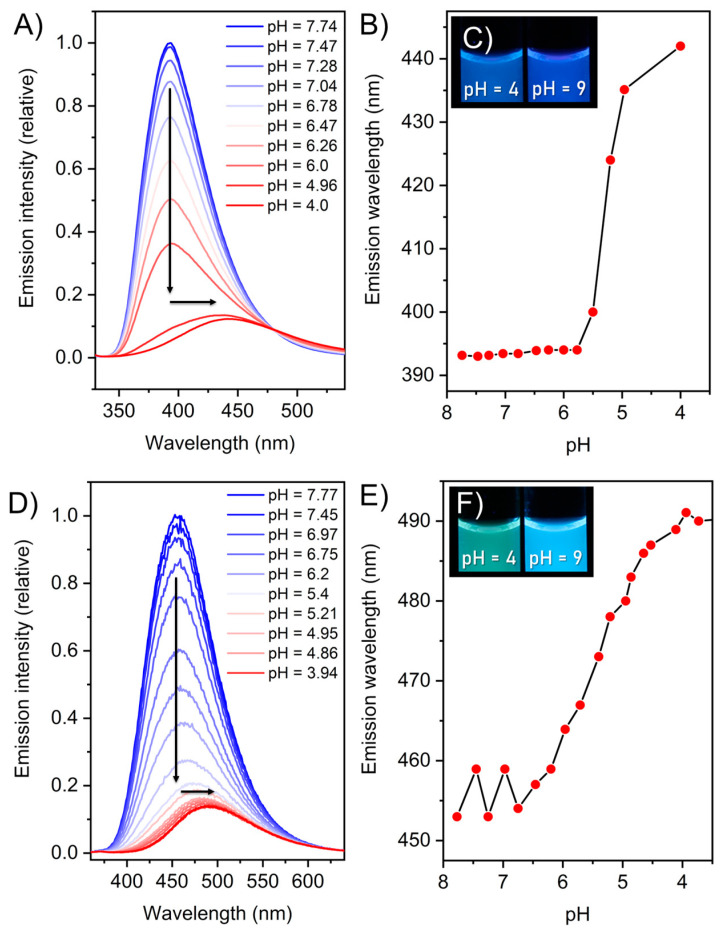
(**A**) Emission spectra of (**2**) at different pH values, blue = high pH, red = low pH. (**B**) Emission maximum of (**2**) plotted against the pH value. (**C**) Photograph of compound (**2**) at pH = 4 and pH = 9 under UV-irradiation (λ_ex_ = 365 nm) (100 µM, bis-tris buffer 6 mM). (**D**) Emission spectra of (**3**) at different pH values, blue = high pH, red = low pH. (**E**) Emission maximum of (**3**) plotted against the pH value. (**F**) Photograph of compound (**3**) at pH = 4 and pH = 9 under UV-irradiation (λ_ex_ = 365 nm) (100 µM, bis-tris buffer 6 mM).

The visible fluorescence of (**2**) and (**3**) is also apparent in the solid state (see ESI Figure S2B,D) and as crystals (see ESI Figure S2A,C). An interesting observation was made when the pH value was changed in aqueous media. Lowering the pH value leads to a significant bathochromic shift of both (**2**) and (**3**) going along with an emission decrease (Figure 5A,D). Compound (**2**) reveals a red shift from 394 nm, with a violet colour at pH = 9 to 442 nm at pH = 4, to a blue colour (Figure 5B,C). In addition, (**3**) shifted from 453 nm to 490 nm upon pH decrease (Figure 5D). This change is even visible to the naked eye, especially in the case of (**3**), where the fluorescence shifts from a blue colour at pH = 9 to a greenish colour at pH = 4 (Figure 5F). To further investigate this phenomenon, photoluminescence quantum yields (PLQY) of buffered solutions at different pH values were measured (pH = 4, 6, 8) (Table 1). For the reference compound (**1**) the PLQY is under the detection limit and, therefore, negligible at pH = 6; however, compound (**2**) demonstrates a quantum efficiency of nearly 15%.

For (**3**), this value was determined to be around 7%. The pH-dependence of the fluorescence becomes especially visible when comparing the PLQY at pH = 4 and pH = 8. For compound (**2**), it roughly doubles from 9% to 17%. For (**3**), this shift is even more apparent as the quantum efficiency is increased with a factor of six from 2.6% at pH = 4 to 15.7% at pH = 8. This observation could be explained by the degree of protonation of the guanidinium unit leading to a persistent positive charge at low pH and to a change in the electronic constitution of the molecule. The naked eye detectable fluorescence switching for compound (**3**) with reasonable quantum yield will provide a suitable platform for the numerous applications compared to compound (**2**). Intrigued by this observation, we proceeded with the determination of pK_a_ values of the corresponding guanidine groups. For (**1**), it was determined as pK_a_ = 6.9 ± 0.2 in a previous work [23] (note: here the butyl amide of (**1**) was used, which should not alter the results, when compared to the ethyl amides of (**2**) and (**3**)) and we determined the values of (**2**) to be pK_a_ = 6.1 ± 0.3 and (**3**) pK_a_ = 6.8 ± 0.5 (Calculated with a pH dependent UV-titration; see ESI Chapter 6.).

As expected, the structural evolution from (**1**) to (**3**) did not lead to a significant change in pK_a_, although the lower value of (**2**) in comparison to (**3**) should be noted.

The pK_a_ values of (**2**) and (**3**) being between 6 and 7 also offers an explanation for the pH-dependence of the emission properties, as it clearly indicates that the protonation of the guanidinium group is responsible for this phenomenon.

We assume, after protonation, that the guanidinium group forms a six-membered ring structure with the acetyl-oxygen, leading to the change in the electronic properties of the system and therefore shifting the emission. This interesting observation might open up further sensing applications for molecules with featuring GCI or MGCI moieties, as the shift occurs mainly between pH = 4 and pH = 7, which is in line for imaging of specific cell organelles known to have pH ranges between 4.5 and 8 in different organelles [36].

**Figure 6:**
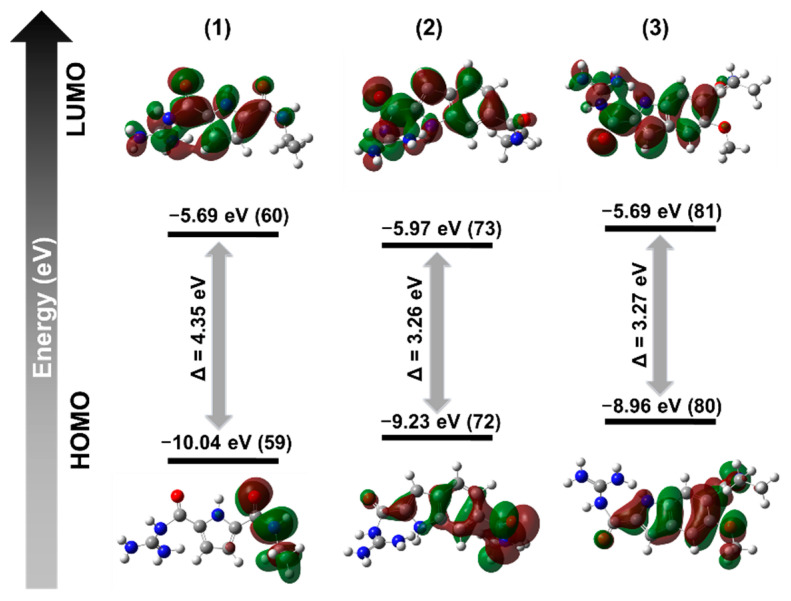
Calculated HOMO/LUMO orbitals and energies of (**1**), (**2**) and (**3**).

The interesting steady state optical properties of (**3**) inspired us to further investigate energy levels and molecular orbitals to their analogues (**1**) and (**2**). For that reason, we carried out density functional theory (DFT with Becke’s three-parameter hybrid exchange functional and the Lee-Yang-Parr correlation functional (B3LYP) and 6-311G (++) (d, p) basis set). The DFT calculations provided information regarding the highest occupied molecular orbital (HOMO) and the lowest unoccupied molecular orbital (LUMO) and their related energy differences (Figure 6). The HOMO and LUMO electron density for (**1**) clearly separates between the non-conjugated ethyl amide segment and indole segment, respectively, which results in higher energy gaps between them (4.35 eV). However, upon the introduction of the guanidiniocarbonyl-indole moiety into the molecule (**2**) and (**3**), the HOMO electron density spreads over the highly conjugated guanidiniocarbonyl-indole moieties in both cases along with tangible reduction of energy gaps. In between molecule (**2**) and molecule (**3**), upon induction of the +M effect of the methoxy group into the domain of the HOMO electron region, the energy level of the HOMO orbital pushes to the upper region along with the LUMO electron density while minimising the energy gaps. Thus, the +M effect of the methoxy group within (**3**) can significantly alter the effective conjugation with the chromophoric segments and, hence, it induced a red shift of the emission maximum.

A detailed investigation of the binding properties of the binding moieties (**2**) and (**3**) in comparison to the known moiety (**1**) was performed using various oxo-anions. We chose acetate and benzoic acid as carboxylates to investigate interactions to aliphatic and aromatic anions. Correspondingly, we chose methylphosphonic acid and phenylphosphonic acid as phosphonates, since GCP is known to bind phosphates [30,37]. Additionally, we investigated methylsulfonic acid and benzenesulfonic acid as sulfonates, due to the structural similarities and the biological relevance of sulphates [38]. In the measured conditions (bis-tris buffer solution at pH = 6), these compounds can be considered deprotonated with one negative charge due to the corresponding pK_a_ values. For the determination of the stoichiometry of the formed noncovalent complexes, we used the method of continuous variation, which is known as a Job plot [39,40]. Hereby, we also expected 1:1 stoichiometries for (**2**) and (**3**), as compound (**1**) is known to form 1:1 complexes with oxo-anions such as carboxylates.

**Figure 7:**
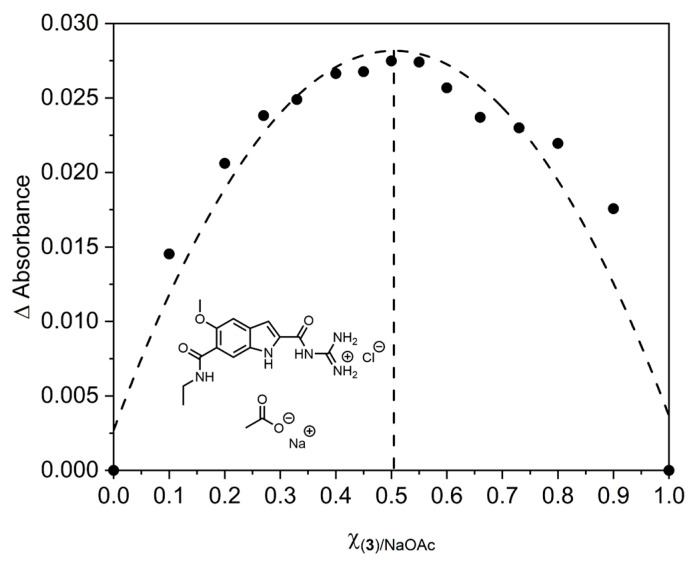
Job plot for the complex formation of (**3**) and sodium acetate which confirms a 1:1 complex stoichiometry (for more information see Supplementary Materials).

We performed Job plots for the three compounds with all six mentioned oxo-anions (see ESI Chapter 5). For all tested combinations, we determined the expected stoichiometry of 1:1, and therefore conclude that (**2**) and (**3**) analogous to (**1**), form 1:1 complexes with these oxo-anions. A representative Job plot of (**3**) with sodium acetate is shown in Figure 7.

**Figure 8:**
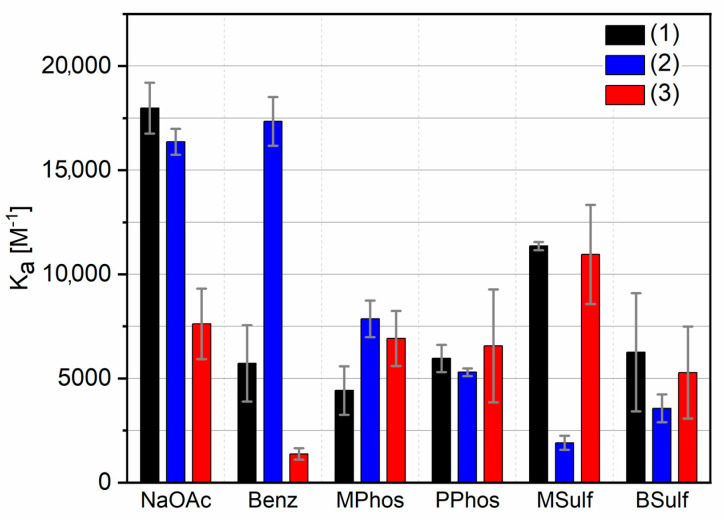
Binding constants of (**1**), (**2**) and (**3**) with different substrates with standard deviations, measured by UV/Vis-Titration. NaOAc: sodium acetate, Benz: benzoic acid, MPhos: methylphosphonic acid, PPhos: phenylphosphonic acid, MSulf: methanesulfonic acid, BSulf: benzenesulfonic acid. Measurements were conducted in bis-tris buffer 6 mM at pH = 6.

UV/Vis titrations were performed in bis-tris buffer solution at pH = 6, which, considering the pK_a_ values, leads to a widespread protonation of the compounds while staying within a neutral range. (For detailed experimental procedure see Chapter 4, ESI.) Only evaluation of 1:1 complexes with the Job’s method delivered suitable values corroborating the formation of complexes with this stoichiometry. Association constants and standard deviations are depicted in Table 2 and Figure 8 (calculation performed with www.supramolecular.org (accessed on 25 May 2021) [41,42]). In this regard, it is important to note that the range of all association constants is between K_ass_ ≈ 1000–18,000 M^−1^, which means that (**2**) and (**3**) show binding affinity to oxo-anions in a similar range to GCP, which is considered to be high in competitive media, such as water or even buffer [43]. The higher associations constants for (**1**) measured in this study compared to previous work (K_ass_ ≈ 18,000 M^−1^ for acetate vs. K_ass_ ≈ 2800 M^−1^) [22] can be easily explained with the measurement conditions. We used a buffer with a defined pH value of 6 in this work, compared to a DMSO/water mixture in the previous work, which should have a pH above 7, due to the low acidity of DMSO. The lower pH value leads to a higher protonation of the guanidinium group and therefore to higher association constants. A certain pH-dependence in oxo-anion recognition of ammonium containing receptors was observed before [44]. Additionally, the addition of a buffer agent can have an influence on the association constant [45]. The comparable values for (**2**) and (**3**) illustrate that these moieties can be used as substitutes in applications in which (**1**) was used previously, even in challenging biological applications, where buffered solutions are needed. Nevertheless, there are some trends which can be observed, which will be discussed vide infra.

Compound (**1**) shows the highest determined affinity towards the acetate anion followed by (**2**) and (**3**). Interestingly, this is not true for the tested aromatic carboxylate—benzoic acid. Here, (**2**) shows the highest association constant, followed by (**1**) and finally (**3**). Remarkably, the binding affinities of (**1**) and (**3**) to benzoic acid are significantly lower than their corresponding values for acetate. An explanation for this behaviour would be mostly sterical reasons, as the aromatic ring of benzoic acid does not fit properly in the binding site of (**1**), shown by the lowest energy structure (see Chapter 8, ESI). In the case of (**2**), additional π-π-stacking with the aromatic substrate is the most plausible explanation for the comparably strong association. In the case of (**3**), with an additional methoxy group attached, as the only structural change, alterations in binding affinity are surprising. We speculate that this group may manipulate π-π-stacking due to sterical reasons.

In the case of the phosphonates (methylphosphonic acid, phenylphosphonic acid), we observe association constants between 4400 and 7900 M^−1^. The differences here are within the range of the standard deviation. Notably, the association constants with phosphonates are generally lower than the values for carboxylates [12], with the exception of (**3**) with the aromatic oxo-anion, where the association constant for phenylphosphonic acid is higher than for benzoic acid. This is not surprising, as such oxo-anions are known to be difficult to recognise, due to their weak basicity, whereas carboxylates normally bind stronger than phosphates and phosphates slightly stronger than sulphates [12]. The high similarity of phosphonates to phosphates and sulphonates to sulphates should be mentioned, especially in their basicity, which allows us to compare these structural elements [37]. Additionally, the hydrogen bond network of GCP was particularly designed for planar oxo-anions such as carboxylates where it fits best to the binding site. For the sulfonates, we generally observed comparable association constants as for the phosphonates, as expected due to the structural similarity and the low basicity. We also observed low association constants for (**2**). Compounds (**1**) and (**3**) show similar values, but the aliphatic sulfonate (methylsulfonic acid) shows higher constants than the aromatic benzene sulfonic acid. This is explainable with sterical interference of the aromatic benzol with the receptor. The strong binding of the sulfonates to (**1**) is a little surprising, as we calculated just three H-bonds in these complexes compared to four in the case of carboxylates and phosphonates, as seen above. It seems that the additional binding strength of the fourth H-bond is negligible, which can be expected, as H-bonds are particularly weak in aqueous solvents [46]. The weak interaction of (**2**) with methylsulfonic acid and benzene sulfonic is unexpected but might be due to the lower pK_a_-value of (**2**) compared to (**1**) or (**3**) which leads to a slightly lower protonation. It seems this effect is overcompensated with the other counterions, and therefore demands further studies.

These observations reveal that the selection of guanidinocarbonyl indoles in further applications should not only be judged by the wanted photophysical properties, but also by the targeted oxo-anions. In an application targeting carboxylates (**2**) is preferable over (**3**), whereas for sulfonates (**3**) is favoured.

## 3. Conclusions

In this work, we investigated the evolution of oxo-anion binders featuring emission properties such as (**2**) and (**3**) in comparison to the known and widely used (**1**).

It was shown that (**2**) and (**3**) bind strongly to carboxylates, phosphonates and sulfonates in aqueous buffered environment and in the same order of magnitude as (**1**) and are therefore adequate emissive alternatives for it. Additionally, they introduce useful optical properties, such as a visible fluorescence with a sufficient PLQY. Therefore, following projects should integrate these fluorescent anion binders in functional structures, as already described for (**1**). Compound (**3**) can potentially be used in biological applications as the excitation is combinable with fluorescence microscopy and will be part of a future investigation. A strong pH-dependence of the optical properties of both moieties, (**2**) and (**3**), allows for sensing applications. An example would be using the emission shift of (**3**) to distinguish between different pH-regions in a cellular environment. However, as in nature, the evolution of artificial arginine analogues is never finished, further studies should focus on shifting the excitation and emission wavelength bathochromically, as this would broaden possible applications, especially in biological fields. For this purpose, the substitution of the methyl-ether group at the C-5 position of the indole in (**3**) comes to mind, using a functional group which has an even stronger +M-effect then the methyl ether, which we assume to be responsible for the mentioned red shift. Amines and their derivatives, such as dimethylamine, are possible candidates [47], which will be investigated in a future contribution.

## Figures and Tables

**Table 1:** Overview over spectroscopic parameters obtained from compounds (**1**)–(**3**).

	λ_abs_^[a]^ ± 5 [nm]	λ_em_ ^[a]^ [nm]	λ_ex_^[a]^ [nm]	Stokes Shift [cm^−1^]	Φ_F_^[d]^ ± 1 [%]	Φ_F_^[b]^ ± 1 [%]	Φ_F_^[e]^ ± 1 [%]
**1**	296	344	299	4714	- ^[c]^	- ^[c]^	- ^[c]^
**2**	314	400	299	6847	8.9	14.6	17.1
**3**	320	468	321	9882	2.6	7.1	15.7

^[a]^ measured in bis-tris buffer 6 mM, pH = 6, concentration 10 µM. ^[b]^ measured in bis-tris buffer 6 mM, pH = 6, concentration 10 µM, average of three independent measurements. ^[c]^ under the detection limit in the experiment. ^[d]^ measured in bis-tris buffer 6 mM, pH = 4, concentration 10 µM, average of three independent measurements. ^[e]^ measured in bis-tris buffer 6 mM, pH = 8, concentration 10 µM, average of three independent measurements. Φ_F_ was determined using an integrated sphere.

**Table 2:** Association constants (K_ass_ and log K) and standard deviations (σ) as calculated with http://supramolecular.org (accessed on 4 April 2022) for the 1:1 complexes formed between (**1**), (**2**) and (**3**) and various oxo-anions in bis-tris buffer 6 mM, pH = 6, average of two independent measurements [41,42].

	(1)			(2)			(3)		
	K_ass_ [M^−1^]	σ [M^−1^]	log K	K_ass_ [M^−1^]	σ [M^−1^]	log K	K_ass_ [M^−1^]	σ [M^−1^]	log K
NaOAc	17,981	1220	4.25	16364	626	4.21	7617	1690	3.88
Benz	5727	1831	3.76	17,339	1173	4.24	1376	273	3.14
Mphos	4421	1164	3.65	7861	881	3.90	6919	1320	3.84
PPhos	5963	653	3.78	5298	181	3.72	6562	2712	3.82
MSulf	11,360	196	4.06	1907	338	3.28	10,957	2378	4.04
BSulf	6258	2841	3.80	3565	665	3.55	5284	2202	3.72

NaOAc: sodium acetate, Benz: benzoic acid, MPhos: methylphosphonic acid, PPhos: phenylphosphonic acid, MSulf: methanesulfonic acid, BSulf: benzenesulfonic acid.

## Data Availability

The data presented in this study are available on request from the corresponding authors.

## Supplementary Materials

The following supporting information can be downloaded at: https://www.mdpi.com/article/10.3390/molecules27093005/s1. References [48,49,50,51,52,53,54,55,56,57,58,59,60] are cited in Supplementary Materials.

## Sample Availability

Not available.
