# Supplementary material for: Evolution of Artificial Arginine Analogues—Fluorescent Guanidiniocarbonyl-Indoles as Efficient Oxo-Anion Binders
Source: Molecules. 2022 May 7;27(9):3005. doi: 10.3390/molecules27093005 (PMC9104999; doi:10.3390/molecules27093005)
Supplement: Supplementary file 1 [file molecules-27-03005-s001.zip › Molecules_ESI__Voskuhl_Giese_2022.pdf]

# Supporting Information

## Evolution of Artificial Arginine Analogues – Fluorescent Guanidiniocarbonyl-Indoles as Efficient Oxo-anion Binders

Daniel Sebena,[a] Kevin Rudolph,[a] Bibhisan Roy,[a] Christoph Wölper,[b] Till Nitschke,[a] Sarah Lampe,[a] Michael Giese[a]* and Jens Voskuhl[a]*

[a] D. Sebena, K. Rudolph, T. Nitschke, S. Lampe, Dr. B. Roy, Jun. Prof. Dr. Michael Giese, Jun.-Prof. Dr. J. Voskuhl, Faculty of chemistry (Organic chemistry) University of Duisburg-Essen, 45117 Essen (Germany), E-mail: michael.giese@uni-due.de. jens.voskuhl@uni-due.de

[b] Dr. C. Wölper, Faculty of chemistry (Inorganic chemistry) Chemistry, University of Duisburg-Essen, 45117 Essen (Germany)

**Table of Content**

**1.** General Information S2

**2.** Synthesis S3

**3.** Photophysical properties S10

**4.** UV-Vis Titrations S12

**5.** Job plots S51

**6.** PK$_a$ determination S55

**7.** X-Ray crystallography S59

**8.** Lowest energy conformations S71

**9.** DFT Calculations S73

**10.** NMR Spectra S84

**11.** Mass Spectra S92

**12.** Analytical HPLC S96

**13.** References S97

## 1. General information

The used solvents were distilled before usage. A TKA MicroPure ultrapure water system was used to obtain Millipore water. Other commercially available reagents were used as obtained unless it was differently stated. For monitoring reactions, TLC on silica gel plates (Macherey-Nagel POLYGRAM SIL G/UV254) was used, at which spot visualization was carried out by UV light (254 nm and 366 nm). For reversed phase column chromatography an Armen Instrument Spot Flash Liquid Chromatography MPLC apparatus with RediSep C-18 Reversed Phase columns was used. Lyophilisation was performed with a Christ Alpha 1-4 LDplus freeze dryer. pH determination experiments were done with a pH-meter 766 Calimatic from Knick. For melting point determination a Büchi Melting-Point B-540 apparatus with open end glass capillary tubes was used, at which the melting points were not corrected. IR spectra were measured using a Varian 3100 FT-IR Excalibur Series. For low resolution ESI mass spectra a Bruker amaZon SL and for high resolution ESI mass spectra a Bruker maXis 4G UHR-TOF was used. Analytical HPLC was carried out on a Dionex HPLC apparatus that consisted of a P680 pump, an ASI-100 automated sample injector and an UVD 340U photodiode array detector with a YMC ODS-AQ column (column size: 150 x 3.0 mm, particle size: 5 µm, pore size: 12 nm). For UV measurements a Jasco *V-550* spectrometer was used. For UV-titrations a DOSTAL DOSY was used for mixing and dosage. For fluorescence measurements a Varian Fluorescence Cary Eclipse and Shimadzu RF-6000 Sprectrofluorophotometer was used. Quantum yields were determined with a Shimadzu RF-6000 Sprectrofluorophotometer with an ISR-100 Fluorescence Integrating Sphere (100 mm) (absolute method). Hereby the emitted light is quantified and set in relation to the irradiated light according to the manufacturer's instructions. NMR spectra were measured using a Bruker DMX 300, AV NEO 400 or AVHD 600 spectrometer. Measurements were recorded at room temperature using DMSO-$d_6$, $CDCl_3$ or $CD_3OD$ as solvent. Chemical shifts are relative to the signals of the corresponding solvent. Coupling constants are given in Hertz (Hz) while the description of the fine structure means: s = singlet, br. s = broad singlet, d = doublet, t = triplet, m = multiplet.

## 2. Synthesis

The synthesis of the previously described GCP **1** [22] and GCI **2** is described in literature[23].

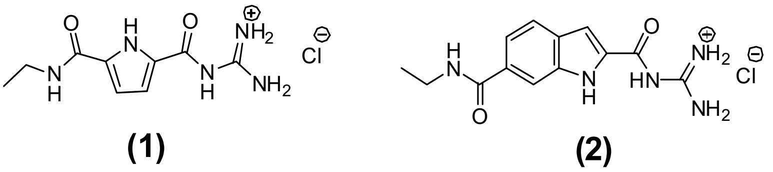


**Scheme S1:** GCP ethylamide **1** and GCI ethylamide **2**.

Methoxy-GCI **3** was synthesized starting from the commercially available methyl 4-iodo-2-methoxybenzoate **A**. The synthesis up to **C** was performed according to a literature procedure [48]. Building block **H** was further functionalized with ethylamine to achieve the MGCI test substance **3**. *N*-[t]Boc-guanidine **(F)** was synthesized as described beforehand [49].

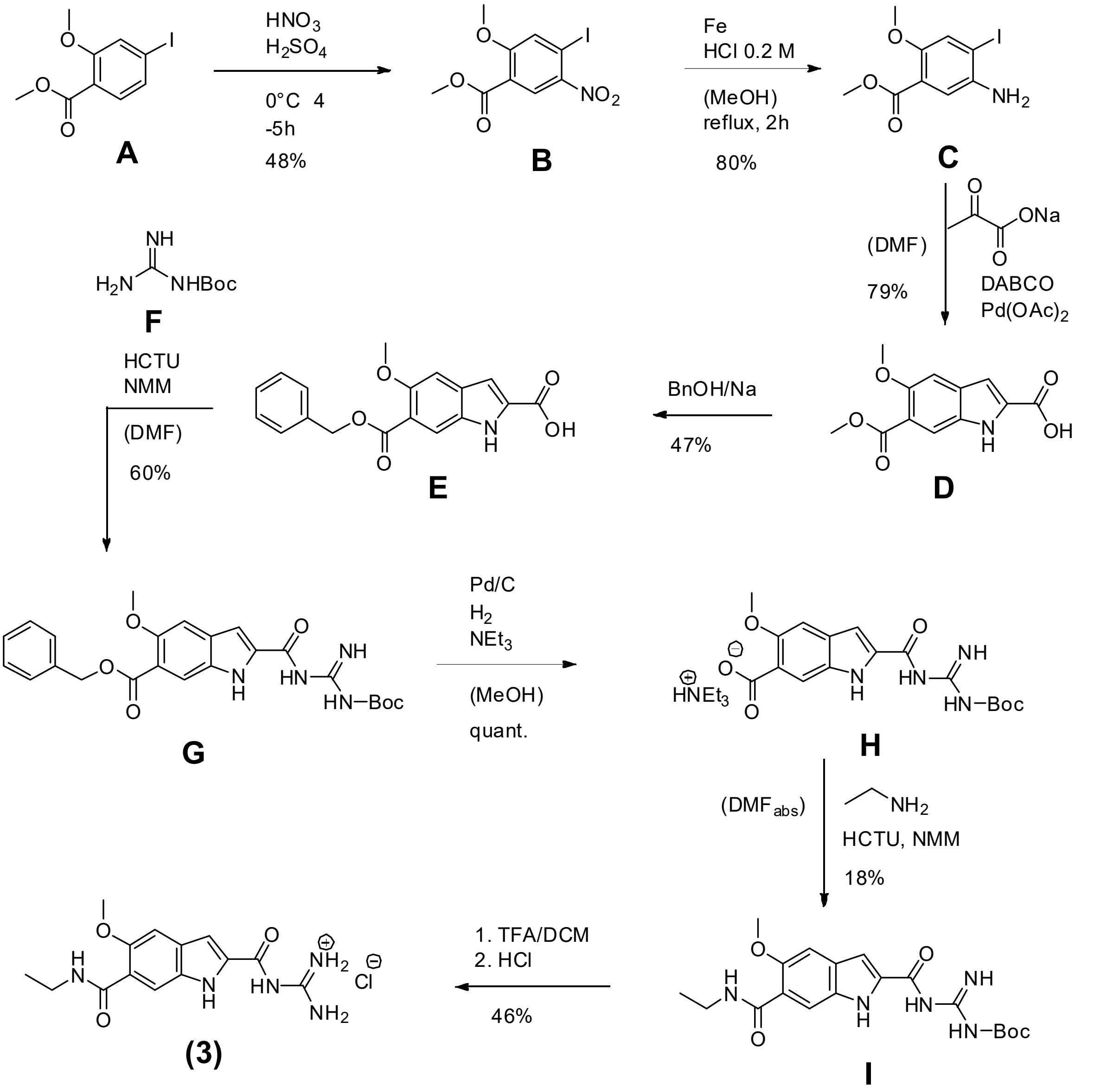


**Scheme S2:** Synthesis of Methoxy-GCI **3**.

**Methyl 4-iodo-2-methoxy-5-nitrobenzoate B**

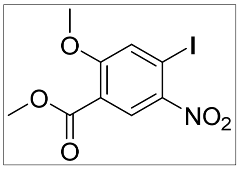


The reaction was performed as described in literature [3]. A cold mixture of $HNO_3$ (2.7 mL, 100%) and $H_2SO_4$ (4.5 mL, 98%) was added slowly to a solution of 4-iodo-2-methoxybenzoate **A** (4.526 g, 15.50 mmol, 1 eq.) in $H_2SO_4$ (7 mL, 98%) with vigorous stirring at 0 °C. The solution was allowed to warm up to room temperature, stirred for 5 h and poured onto crushed ice and $H_2O$ (500 mL). The aquatious layer was extracted with EtOAc (3 x 200 mL), dried over $Na_2SO_4$ and dried *in vacuuo*. The crude product was purified by column chromatography ($SiO_2$, ethyl acetate/cyclohexane = 2/1) to give **B** (2.516 g, 7.46 mmol, 48%) as a yellow solid.

**Molecular Formula:**$C_9H_8INO_5$.

**Molecular Mass:** 337.068 g/mol.

**$^1$H-NMR** (300 MHz, DMSO-d$_6$): δ [ppm] = 8.28 (s, 1H, C*H*), 7.81 (s, 1H, C*H*), 3.96 (s, 3H, C*H₃*), 3.82 (s, 3H,C*H₃*).

**$^{13}$C-NMR** (75 MHz, DMSO-d$_6$): δ [ppm] =163.81, 160.23, 144.84, 127.79, 125.41, 119.69, 95.76, 57.23, 52.46.

**HR-MS:** (pos. ESI, MeOH) m/z = 337.9530 ($[M+H]^+$, calc.: 337.9520).

**FT-IR:** (ATR) ṽ [cm$^{-1}$] = 2952.48 (w), 2919.7 (w), 2848.35 (w), 1697.05 (s), 1589.06 (m), 1540.85 (s), 1508.06 (m), 1475.28 (m), 1434.78 (m), 1376.93 (w), 1336.43 (s), 1247.72 (s), 1130.08 (m), 1103.08 (m), 1012.45 (m), 904.451 (m), 856.239 (m), 819.598 (m), 784.886 (m), 746.317 (m), 682.677 (m), 640.251 (m).

**mp:** 157 °C.

**Methyl 5-amino-4-iodo-2-methoxybenzoate C**

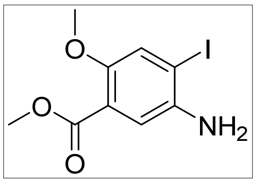


The reaction was performed as described the in literature [3]. Methyl 4-iodo-2-methoxy-5-nitrobenzoate **B** (2.406 g, 7.14 mmol, 1 eq.) was dissolved in MeOH (180 mL). HCl (0.2 M, 71 mL, 14.28 mmol, 2 eq.) and iron powder (3.2 g, 57.34 mmol, 8 eq.) was added at room temperature and the mixture was heated under reflux for 2 h. After filtration through Celite® the solvent was removed *in vacuo*. The crude product was taken up in EtOAc (300 mL) and washed with a saturated $NaHCO_3$-solution (300 mL). The organic layer was dried over $Na_2SO_4$, concentrated *in vacuuo* and purified by column chromatography ($SiO_2$, ethyl acetate/cyclohexane = 1/2) to give **C** (1.751 g, 5.7 mmol, 80%) as a colourless solid.

**Molecular Formula:** $C_9H_{10}INO_3$.

**Molecular Mass:** 307.085g/mol.

**$^1$H-NMR** (400 MHz, $CDCl_3$): δ [ppm] = 7.27 (s, 1H, C*H*), 7.20 (s, 1H, C*H*), 3.86 (s, 3H, C*H₃*), 3.81 (s, 3H, C*H₃*).

**$^{13}$C-NMR** (101 MHz, $CDCl_3$): δ [ppm] = 166.31, 151.85, 140.85, 123.60, 121.21, 116.95, 89.67, 57.14, 52.31.

**HR-MS:** (pos. ESI, MeOH) m/z = 307.9798 ($[M+H]^+$, calc.: 307.9778).

**FT-IR:** (ATR) ṽ [$cm^{-1}$] = 3392.17 (w), 3318.89 (w), 2944.77 (w), 1685.48 (s), 1594.84 (m), 1492.63 (s), 1461.78 (m), 1430.92 (s), 1396.21 (s), 1251.58 (s), 1220.72 (s), 1097.3 (s), 1022.09 (s), 904.451 (m), 885.166 (m), 856.239 (m), 833.098 (m), 782.958 (s), 703.89 (s), 667.25 (s), 622.895 (s), 607.467 (s).

**mp:** 92 °C.

**5-Methoxy-6-(methoxycarbonyl)-1H-indole-2-carboxylic acid D**

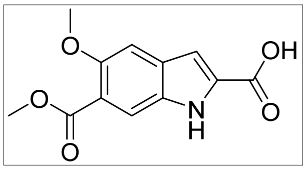


To freshly sublimed DABCO (0.42 g, 3.75 mmol, 3 eq.), sodium pyruvate (0.687 g, 6.25 mmol, 3 eq.) and methyl 3-amino-4-iodobenzoate **C** (0.385 g, 1.25 mmol, 1 eq.) 25 mL degassed dry DMF was added under argon atmosphere. Palladium (II) acetate (0.022 g, 0.1 mmol, 0.08 eq.) was added and the mixture was heated to 105 °C for 19 h. After cooling to room temperature 50 mL water was added, the solution was acidified with 1 M HCl to pH = 2 and extracted with ethyl acetate (5x30 mL). The combined organic layers were washed with brine (2x30 mL) and water (2x30 mL) and the solvent was evaporated *in vacuo* to obtain a brown solid. The crude product was purified by column chromatography (SiO2, ethyl acetate/cyclohexane = 2/1 + 1% acetic acid) to give **D** (3.321 g, 15.15 mmol, 79%) as a yellow solid.

**Molecular Formula:** $C_{12}H_{11}NO_5$.

**Molecular Mass:** 249.219 g/mol.

**$^1$H-NMR** (400 MHz, DMSO-$d_6$): δ [ppm] = 13.15 (s, 1H, COO*H*), 11.90 (s, 1H, N*H*), 7.75 (s, 1H, C*H*), 7.26 (s, 1H, C*H*), 7.03 (dd, *J* = 2.1, 0.8 Hz, 1H, C*H*), 3.80 (s, 6H, 2C$H_3$).

**$^{13}$C-NMR** (101 MHz, DMSO-$d_6$): δ [ppm] = 166.53, 162.45, 152.37, 131.42, 131.13, 129.82, 118.79, 115.35, 106.57, 103.10, 55.97, 51.89.

**HR-MS:** (pos. ESI, MeOH) m/z = 250.0698 ($[M+H]^+$, calc.: 250.0710).

**FT-IR** (ATR) ṽ [$cm^{-1}$] = 3340.1 (w), 1689.34 (m), 1629.55 (w), 1536.99 (w), 1494.56 (w), 1425.14 (w), 1334.5 (w), 1257.36 (m), 1234.22 (m), 1209.15 (m), 1166.72 (w), 1025.94 (w), 823.455 (m), 759.816 (m), 723.175 (m), 640.251 (w), 611.324 (w).

**mp**: 270 °C.

**6-((benzyloxy)carbonyl)-5-methoxy-1H-indole-2-carboxylic acid E**

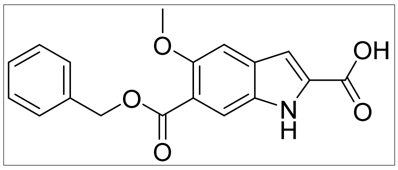


A soldium benzylate solution, prepared from Na (0.103 g, 4.46 mmol, 2.4 eq.) in dry benzyl alcohol (35 mL) was added to **D** (0.463 g, 1.86 mmol, 1 eq.) under Ar. The resulting dark brown solution was stirred at 95 °C for 2h. Thereafter vacuum was introduced and the solvent was partially removed with a destillation bridge under Ar for further 2h. The solution was cooled to room temperature, treated with 1 M hydrochlorid acid to pH = 1 and the solvent was removed *in vacuo*. The crude product was purifed by MPLC chromatography (RP-18 MeOH/H2O + 0.1% TFA, 50% MeOH to 100% MeOH, gradient) to give **E** (0.286 mg, 88 mmol, 47%) as a yellow solid.

**Molecular Formula:** $C_{18}H_{15}NO_5$.

**Molecular Mass:** 325.315g/mol.

**$^1$H-NMR** (400 MHz, DMSO-$d_6$): δ [ppm] = 13.13 (s, 1H, COO*H*), 11.86 (s, 1H, N*H*), 7.81 (s, 1H, C*H*), 7.51 – 7.31 (m, 5H, Ar-*H*), 7.28 (s, 1H, C*H*), 7.04 (d, *J* = 1.2 Hz, 1H, C*H*), 5.31 (s, 2H, C$H_2$), 3.81 (s, 3H, C$H_3$).

**$^{13}$C-NMR** (101 MHz, DMSO-$d_6$): δ [ppm] =165.81, 162.43, 152.55, 136.46, 131.55, 131.16, 129.99, 128.49, 127.98, 127.85, 118.59, 115.54, 106.58, 103.24, 65.90, 56.04.

**HR-MS:** (pos. ESI, MeOH) m/z = 348.0867 ([M+Na]$^+$, calc.: 348.0842).

**FT-IR** (ATR) ṽ [cm$^{-1}$] = 3328.53 (w), 2931.27 (w), 1683.55 (s), 1625.7 (m), 1538.92 (m), 1430.92 (m), 1249.65 (s), 1205.29 (s), 1166.72 (m), 829.241 (m), 763.673 (m), 723.175 (s), 628.68 (m).

**mp**: 232 °C.

**Benzyl2-((N-(tert-butoxycarbonyl)carbamidoyl)carbamoyl)-5-methoxy-1H-indole-6-carboxylate G**

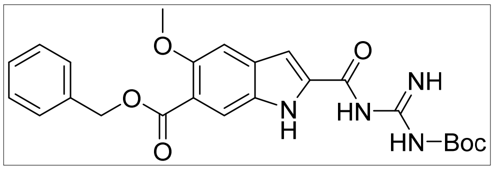


A mixture of the benzyl ester **E** (0.335 g, 1.03 mmol, 1 eq.), HCTU (0.852 g, 2.06 mmol, 2 eq.) and NMM (0.343 mL, 3.09 mmol, 3 eq.) was stirred in DMF abs. (20 mL) at room temperature for 15 min. $^t$Boc-guanidine **F** [49] (0.246 g, 1.54 mmol, 1.5 eq.) was added and the resulting solution stirred at room temperature. After 19 h the solvent was removed *in vacuo*. The residue was solved in EtOAc (150 mL) and washed with $NaHCO_3$ solution (2 x 50 mL). The solvent was removed *in vacuo* and the crude product was purifed by MPLC chromatography (RP-18 MeOH/$H_2O$, 10% MeOH to 100% MeOH, gradient) to give **G** (0.289 g, 0.620 mmol, 60%) as a slight yellow solid.

**Molecular Formula:** $C_{24}H_{26}N_4O_6$.

**Molecular Mass:** 466.486 g/mol.

**$^1$H-NMR** (600 MHz, DMSO-$d_6$): δ [ppm] = 11.54 (s, 1H, indole-N*H*), 10.98 (s, 1H, N*H*), 9.45 (s, 1H, N*H*), 8.61 (s, 1H, N*H*), 7.84 (s, 1H, C*H*), 7.47 (d, *J* = 7.4 Hz, 2H, Ar-*H*), 7.41 (t, *J* = 7.6 Hz, 2H, Ar-*H*), 7.35 (t, *J* = 7.3 Hz, 1H, Ar-*H*), 7.26 (s, 1H, C*H*), 7.08 (s, 1H, C*H*), 5.31 (s, 2H, C$H_2$), 3.81 (s, 3H, C$H_3$), 1.48 (s, 9H, Boc-C$H_3$).

**$^{13}$C-NMR** (101 MHz, DMSO-d$_6$): δ [ppm] = 165.87, 158.63, 152.54, 136.54, 131.07, 130.39, 128.48, 127.95, 127.83, 117.79, 115.53, 105.25, 103.31, 81.70*, 65.79, 56.09, 27.75.**

*Broad signal only visible via 2D-HMBC.

**Only 17 of 20 carbons detected. Carbonyl- and Guanidino-carbon signals may broaden because of dynamic hydrogen bonds and were not traceable by 2D-NMR experiments.

**HR-MS:** (pos. ESI, MeOH) m/z = 467.1926 ([M+H]$^+$ , calc.: 467.1925).

**FT-IR** (ATR) ṽ [cm$^{-1}$] = 2973.7 (w), 2935.13 (w), 2869.56 (w), 1716.34 (w), 1621.84 (m), 1542.77 (m), 1508.06 (m), 1455.99 (m), 1417.42 (w), 1369.21 (m), 1232.29 (s), 1143.58 (s), 1056.8 (m), 836.955 (m), 779.101 (m), 730.889 (m), 696.177 (m), 642.18 (m).

**mp**: 129 °C (decomposition).

**Methoxy-GCI building block H**

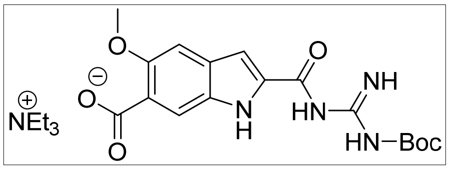


A mixture of the benzyl ester **G** (0.252 g, 0.54 mmol, 1 eq.) and 10 % Pd/C (10 mg) in 100 mL Methanol and 5 mL triethylamine was vigorously stirred under hydrogen atmosphere for 16 h. The resulting solution was filtered through a folded filter and washed with methanol/triethylamine. The solvent was removed under reduced pressure yielding the Methoxy-GCI building block **H** as an off-white solid (259 mg, 0.54 mmol, quant.).

**Molecular Formula:** $C_{23}H_{35}N_5O_6$.

**Molecular Mass:** 477.554g/mol.

**$^1$H-NMR** (600 MHz, DMSO-d$_6$): δ [ppm] = 11.43 (s, 1H, indole-N*H*), 9.38 (s, 1H, N*H*), 8.60 (s, 1H, N*H*), 7.52 (s, 1H, C*H*), 7.10 (s, 2H, *2CH*), 3.75 (s, 3H, C*H*$_3$), 3.39 (q, *J* = 7.1 Hz, 6H, NEt$_3$-C*H*$_2$), 1.47 (s, 9H, Boc-C*H*$_3$), 1.20 (t, *J* = 7.2 Hz, 9H, NEt$_3$-C*H*$_3$).

**$^{13}$C-NMR** (151 MHz, DMSO-d$_6$): δ [ppm] = 169.98, 158.54, 155.83, 151.82, 135.71, 131.69, 127.91, 113.24, 105.60, 102.52, 80.98, 57.55, 55.84, 27.79, 7.79.*

*Only 15 of 17 carbons detected. Carbonyl- and Guanidino-carbon signals may broaden because of dynamic hydrogen bonds and were not traceable by 2D-NMR experiments.

**HR-MS:** (pos. ESI, MeOH) m/z = 377.1473 ([M+H]$^+$, calc.: 377.1456).

**FT-IR** (ATR) ṽ [cm$^{-1}$] = 3378.67 (w), 2977.55 (w), 2937.06 (w), 1621.84 (m), 1548.56 (m), 1500.35 (m), 1450.21 (m), 1367.28 (m), 1234.22 (s), 1145.51 (s), 836.955 (m), 779.101 (m), 619.038 (m), 605.539 (m).

**mp**: 101 °C (decomposition).

**Boc-Methoxy-GCI-ethylamide I**

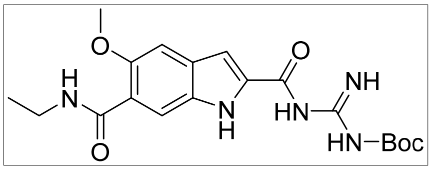

The Methoxy-GCI building block **H** (0.23 g, 0.482 mmol, 1 eq.) and HCTU (0.399 g, 0.964 mmol, 2 eq.) was solved in DMF abs. (20 mL) and NMM (0.16 mL, 1.446 mmol, 3 eq.) was added. After stirring the solution at room temperature ethylamine (0.36 mL, 0.723 mmol, 1.5 eq., 2M solution in THF) was added and the resulting solution was stirred at room temperature for 17 h. The solvent was removed *in vacuo* and the crude product was purified by MPLC chromatography (RP-18 MeOH/$H_2O$, 50% MeOH to 100% MeOH, gradient) to give **I** (40 mg, 0.086 mmol, 18 %) as a off-white solid.

**Molecular Formula:** $C_{19}H_{25}N_5O_5$.

**Molecular Mass:** 403.432g/mol.

**$^1$H-NMR** (600 MHz, DMSO-$d_6$): δ [ppm] = 11.48 (s, 1H, indole-N*H*), 10.88 (s, 1H, N*H*), 9.46 (s, 1H, N*H*), 8.57 (s, 1H, N*H*), 8.25 (t, *J* = 5.5 Hz, 1H, ethyl-N*H*), 7.93 (s, 1H, C*H*), 7.23 (s, 1H, *H*-4), 7.03 (s, 1H, C*H*), 3.89 (s, 3H, O-C$H_3$), 3.34 – 3.28* (m, 2H, C$H_2$), 1.48 (s, 9H, Boc-C$H_3$), 1.13 (t, *J* = 7.2 Hz, 3H, ethyl-C$H_3$).

*****Signal is partially covered by the water signal of DMSO and appears as shoulder.

**$^{13}$C-NMR** (151 MHz, DMSO-$d_6$): δ [ppm] = 165.16, 158.56, 151.56, 131.78, 129.15, 121.16, 114.98, 105.27, 102.39, 82.18*, 55.99, 33.98, 27.77, 14.92.**

*Broad signal only visible via 2D-HMBC.

**Only 14 of 17 carbons detected. Carbonyl- and Guanidino-carbon signals may broaden because of dynamic hydrogen bonds and were not traceable by 2D-NMR experiments.

**HR-MS:** (pos. ESI, MeOH) m/z = 404.1955 ($[M+H]^+$, calc.: 404.1928).

**FT-IR** (ATR) ṽ [$cm^{-1}$] = 3365.17 (w), 2933.2 (w), 1621.84 (m), 1508.06 (m), 1455.99 (m), 1238.08 (s), 1147.44 (s), 1122.37 (s), 1091.51 (s), 1041.37 (m), 844.669 (m).

**mp**: 188 °C.

**Methoxy-GCI-ethylamide (3)**

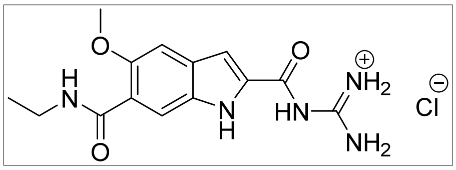

**I** (36 mg, 0.077 mmol, 1 eq.) was solved in Dichlormethane (3 mL). TFA (3 mL) was added and the reaction mixture was stirred at room temperature for 3 h. The solvent was removed with reduced pressure and the crude product was purified by MPLC chromatography (RP-18 MeOH/$H_2O$ + 0.1% TFA, 30% MeOH to 100% MeOH, gradient). HCl (5 mL, 0.1 M) was added and removed under reduced pressure 3 times to the purified product to give **3** (12 mg, 0.035 mmol, 46%) as a yellow solid with a purity of 96% (HPLC).

**Molecular Formula:** $C_{14}H_{18}ClN_5O_3$.

**Molecular Mass:** 339.777g/mol.

**$^{1}$H-NMR** (600 MHz, DMSO-$d_6$): δ [ppm] = 12.22 (s, 1H, N*H*), 12.13 (s, 1H, N*H*), 8.72 (s, 2H, N$H_2$), 8.48 (s, 2H, N$H_2$), 8.29 (t, *J* = 5.2 Hz, 1H, ethyl-N*H*), 7.91 (s, 1H, C*H*), 7.82 (s, 1H, C*H*), 7.31 (s, 1H, C*H*), 3.89 (s, 3H, O-C$H_3$), 3.30-3.50 (m, 2H, C$H_2$)*, 1.12 (t, *J* = 7.0 Hz, 3H, ethyl-C$H_3$).

***Signal is partially covered by the water signal of DMSO and appears as shoulder.

**$^{13}$C-NMR** (101 MHz, DMSO-$d_6$): δ [ppm] = 164.85, 160.90, 155.43, 151.82, 132.52, 130.18, 128.22, 123.71, 114.87, 107.34, 102.58, 56.00, 33.94, 14.77.

**HR-MS:** (pos. ESI, MeOH) m/z = 304.1412 ($[M+H]^+$, calc.: 304.1404).

**FT-IR** (ATR) ṽ [$cm^{-1}$] = 2894.63 (w), 2836.77 (w), 2715.28 (w), 2647.79 (w), 2557.15 (m), 2063.46 (w), 1947.75 (w), 1814.69 (w), 1685.48 (m), 1635.34 (m), 1542.77 (m), 1481.06 (m), 1224.58 (m), 1087.66 (s), 674.963 (m).

**mp**: 177 °C.

## 3. Photophysical properties

### Absorption, emission and excitation spectra

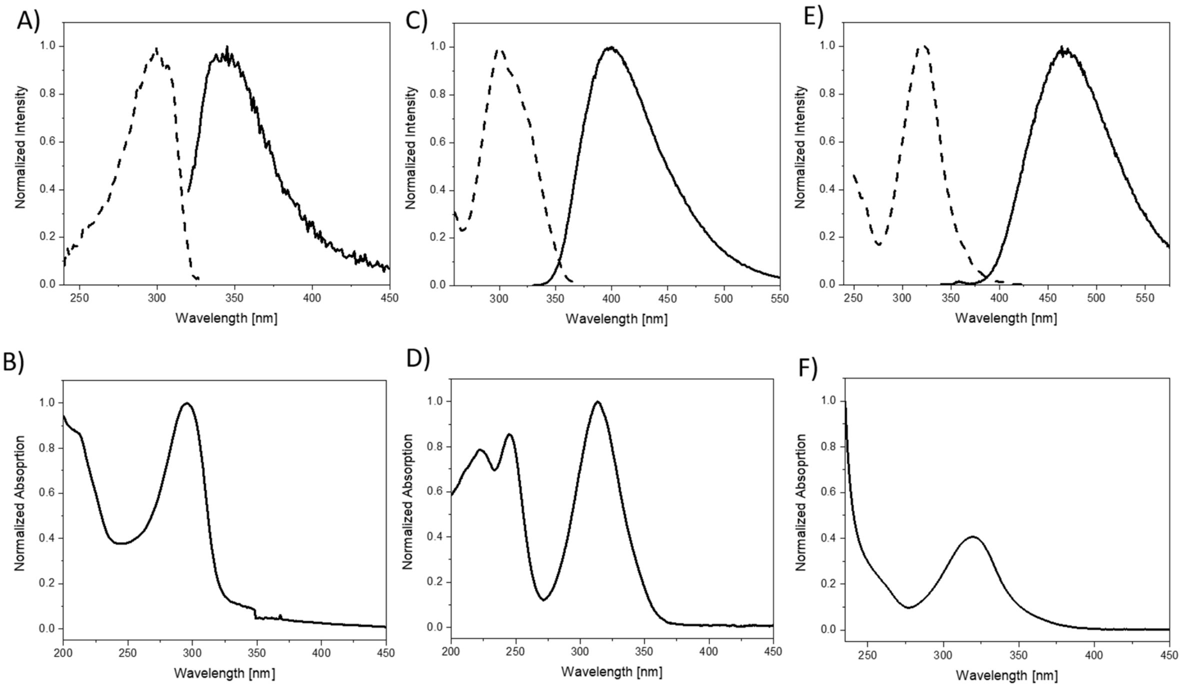


**Figure S1.** A) Excitation and emission spectra of **1**, B) UV/Vis-absorption spectrum of **1**, C) Excitation and emission spectra of **2**, D) UV/Vis-absorption spectrum of **2**, E) Excitation and emission spectra of **3**, F) UV/Vis-absorption spectrum of **3**. (dashed line: excitation, solid line: emission). Spectra were recorded in bis-tris buffer 6mM, pH = 6, concentration 10 µM.

| Compound | $\lambda_{abs}^{(a)}$ ± 5 [nm] | $\lambda_{em}^{(a)}$ [nm] | $\lambda_{ex}^{(a)}$ [nm] |
|---|---|---|---|
| **1** | 296 | 344 | 299 |
| **2** | 314 | 400 | 299 |
| **3** | 320 | 468 | 321 |

**Table S1.** Overview about the excitation (most bathochromic), emission (maximum) and absorption (maximum) wavelengths obtained from all samples in bis-tris buffer 6mM, pH = 6, concentration 10 µM.

## Quantum Efficiency

Quantum Efficiency was measured with a Shimadzu RF-6000 Sprectrofluorophotometer with integration sphere. Hereby solutions of **1, 2** and **3** (10 µM, bis-tris buffer 6mM, pH = 4 or pH = 8) were measured in triplicate.

| pH | compound | Measurement 1 $\phi_F$ [%] | Measurement 2 $\phi_F$ [%] | Measurement 3 $\phi_F$ [%] |
|---|---|---|---|---|
| 4 | **1** | -[a] | -[a] | -[a] |
| | **2** | 8.83 | 8.73 | 9.09 |
| | **3** | 2.58 | 2.55 | 2.57 |
| 8 | **1** | -[a] | -[a] | -[a] |
| | **2** | 16.74 | 17.07 | 17.42 |
| | **3** | 15.58 | 15.81 | 15.80 |
| (a) under detection limit in the experiment. | | | | |

**Table S2.** Measurements of Quantum Efficiency, 10 µM, bis-tris buffer 6 mM, pH = 4 or pH = 8.

## Photographs

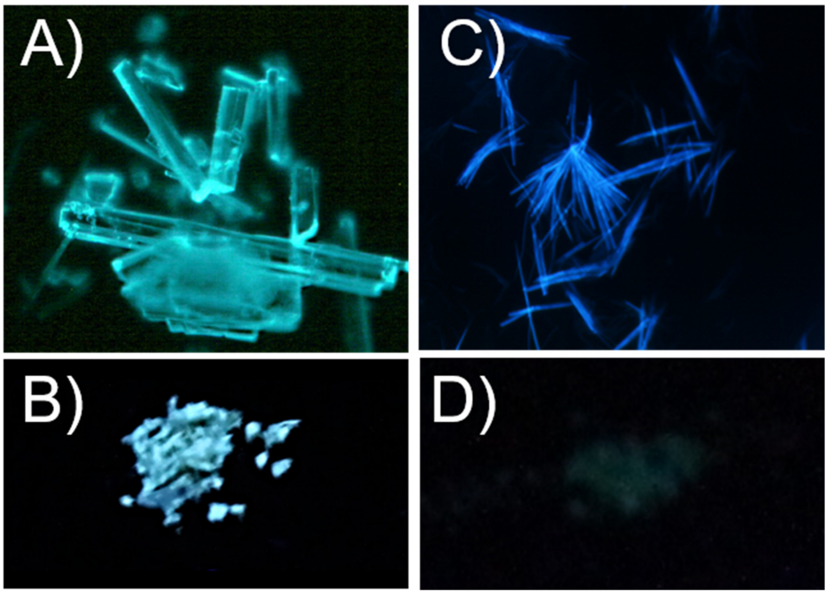


**Figure S2.** (A) crystallized (**2**) as hydrochloride salt under 365 nm irradiation. (B) Solid (**2**) irradiated at 365 nm. (C) Crystallized (**3**) as TFA salt irradiated at 365 nm (not suitable for x-ray diffraction). (D) Solid (**3**) irradiated at 365 nm.

## 4. UV/Vis-Titrations

All spectra were carried out in aqueous 2-Bis(2-hydroxyethyl)amino-2-(hydroxymethyl)-1,3-propanediole (bis-tris) buffer solution (0.006 M, pH = 6.00 ± 0.01) in quartz UV cuvettes (1 cm). The pH was adjusted with aqueous HCl or NaOH. A solution of the substrate (sodiumacetate, benzoic acid, methylphosphonic acid, phenylphosphonic acid, methanesulfonic acid, benzenesulfonic acid, 10 µL, 8.0 x $10^{-3}$ M) was loaded into a DOSTAL DOSY© titration device. A solution of the receptor (**1**, **2**, **3**) (400 µL, 2.0 x $10^{-5}$ M) was placed in a microcuvette. The capillaries of the DOSY were inserted into the microcuvette and secured with Parafilm©. 3 spectra were measured at controlled temperature (T = 25 °C) between λ =530-230 nm. Using the DOSTAL DOSY© 0.25 µL of the substrate solution was inserted into the receptor solution and mixed (3 x 300 µL) with the same device. Afterwards 3 spectra of this mix ratio were measured. This cycle was repeated 40 times. The average of the 3 spectra with the same mix ratio was used for further assessment. Hereby the spectra were adjusted to zero at 530 nm, as the components do not absorb at this wavelength. To determine the binding constants K the data was analysed over a spectral range characteristic for the receptor (**1**: λ = 280-310 nm, **2**: λ = 290-330 nm, **3**: λ = 290-330 nm) with the aid of the website: http://supramolecular.org. Herby the fitter UV 1:1 with the fitting method Nelder-Mead with dilution correction was used. For a deeper understanding concerning the corresponding concepts, one may consult the review article by P. Thordarson [50]. All measurements were performed in duplicate.

We performed a blank UV-titration in which we added buffer solution to one of the receptors (**3**). We expected a linear reduction of absorbance due to dilution. Instead we observed a non-linear behaviour at the first two data points, whereas after these two data points we observe the linear decrease we expected (Figure S4). We associated this with the experimental setup with the DOSTAL DOSY©, as other works with similar host-guest systems but without the use of the titration device did not lead to such a phenomenon[25]. Our explanation is, that the capillaries of the DOSTAL DOSY© interfere with the absorption at the first two data points due to movement during mixing. After two additions this seems to be negligible. Due to this fact we decided to plot all measurements without the first two data points as we consider these to be flawed.

**Blank measurement (Titration of buffer to (3))**

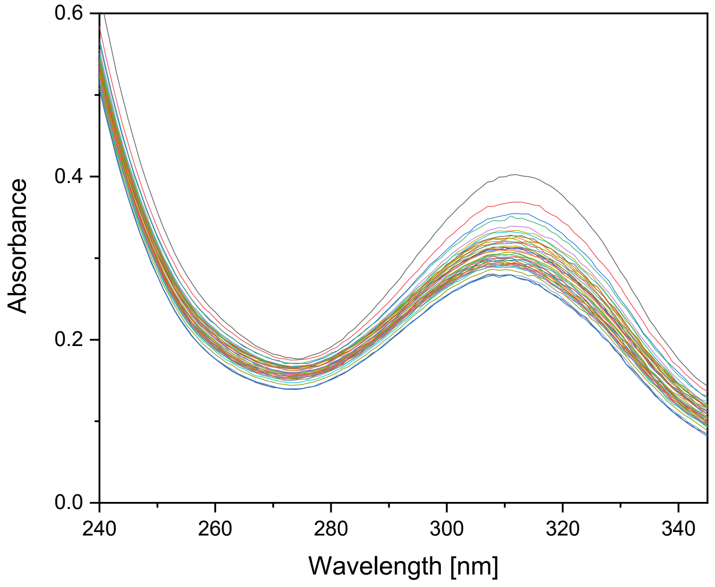


**Figure S3.** Change of UV/Vis spectrum of **(3)** upon incremental addition of bis-tris buffer in aqueous bis-tris buffer at pH = 6.

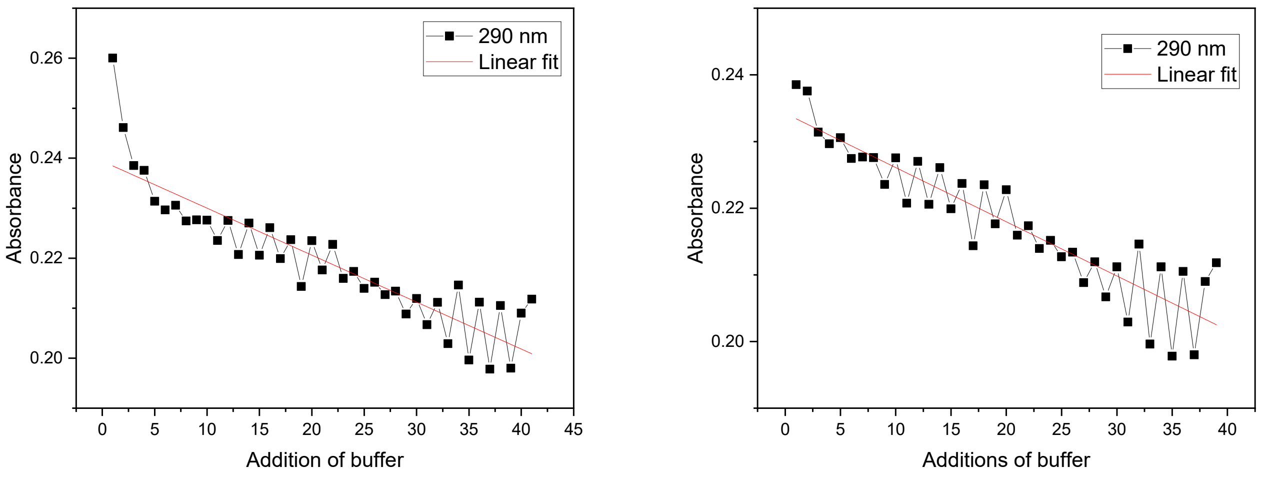


**Figure S4.** left: absorbance at 290 nm with all data points, right: absorbance at 290 nm without the first 2 data points vs. buffer injections.

**NaOAc to (1) measurement 1**

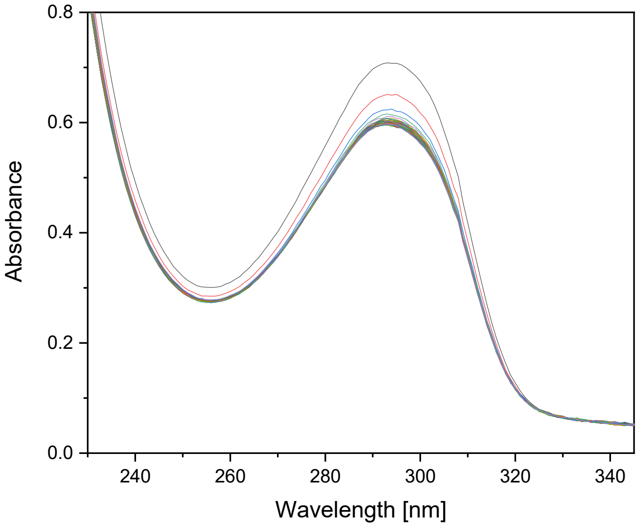


**Figure S5.** Change of UV/Vis spectrum of **(1)** upon incremental addition of NaOAc in aqueous bis-tris buffer at pH = 6.

Fitting parameters of bindfit fitter UV 1:1 of the range 280-310nm without the first 2 values:

| **Parameter (bounds)** | **Optimised** | **Error** | **Initial** |
|---|---|---|---|
| **K** ( 0 → ∞ ) | 16760.58 $M^{-1}$ | ± 1.0400 % | 100.00 $M^{-1}$ |

**Link to the calculation**: http://app.supramolecular.org/bindfit/view/8ad42125-33b2-4188-a734-15d363c5e767

**NaOAc to (1) measurement 2**

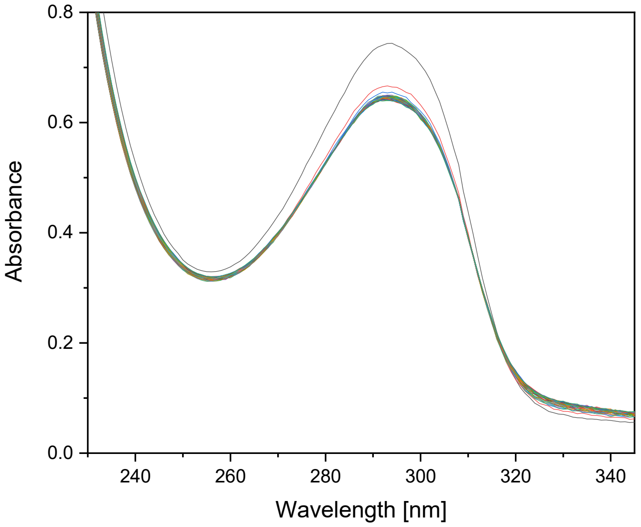


**Figure S6.** Change of UV/Vis spectrum of **(1)** upon incremental addition of NaOAc in aqueous bis-tris buffer at pH = 6.

Fitting parameters of bindfit fitter UV 1:1 of the range 280-310nmwithout the first 2 values:

| Parameter (bounds) | Optimised | Error | Initial |
|---|---|---|---|
| **K** ( 0 → ∞ ) | 19201.36 $M^{-1}$ | ± 1.7059 % | 100.00 $M^{-1}$ |

**Link to the calculation**: http://app.supramolecular.org/bindfit/view/5f5a1f60-f40f-4b41-be1f-c9c568ded4a6

**Benzoic acid to (1) measurement 1**

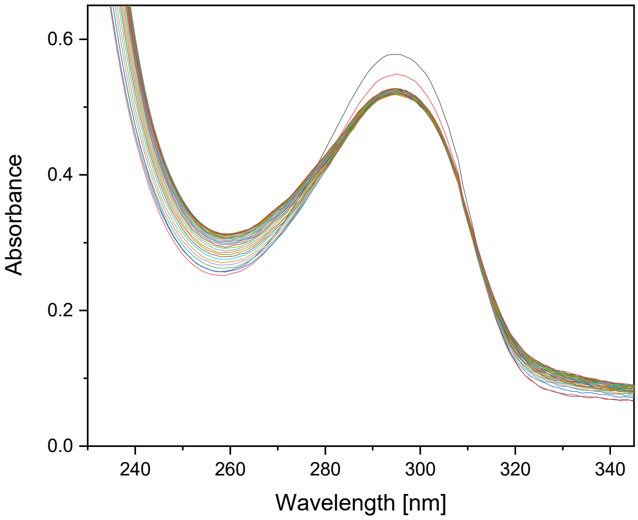


**Figure S7.** Change of UV/Vis spectrum of **(1)** upon incremental addition of benzoic acid in aqueous bis-tris buffer at pH = 6.

Fitting parameters of bindfit fitter UV 1:1 of the range 280-310nm without the first 2 values:

| Parameter (bounds) | Optimised | Error | Initial |
|---|---|---|---|
| **K** ( 0 → ∞ ) | 7022.15 $M^{-1}$ | ± 3.6943 % | 100.00 $M^{-1}$ |

**Link to the calculation**: http://app.supramolecular.org/bindfit/view/61d1e506-2e13-43b6-be86-3a8a917aec0c

**Benzoic acid to (1) measurement 2**

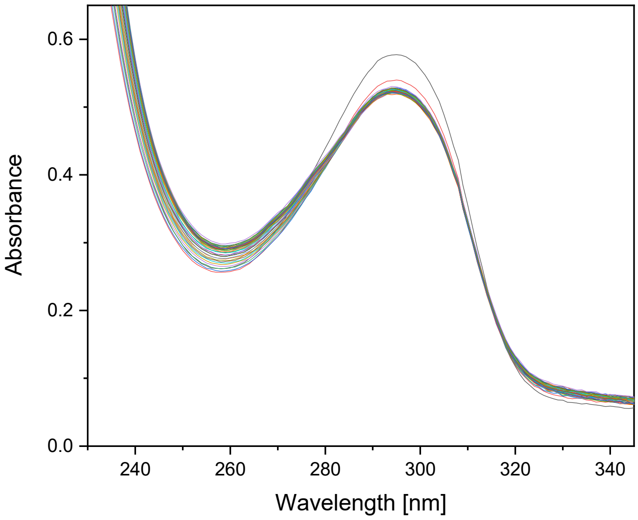


**Figure S8.** Change of UV/Vis spectrum of **(1)** upon incremental addition of benzoic acid in aqueous bis-tris buffer at pH = 6.

Fitting parameters of bindfit fitter UV 1:1 of the range 280-310nm without the first 2 values:

| Parameter (bounds) | Optimised | Error | Initial |
|---|---|---|---|
| **K** ( 0 → ∞ ) | 4432.12 $M^{-1}$ | ± 2.8359 % | 100.00 $M^{-1}$ |

**Link to the calculation**: http://app.supramolecular.org/bindfit/view/6c4ffaa9-cc15-433b-995e-34db9c4b8a97

**Methylphosphonic acid to (1) measurement 1**

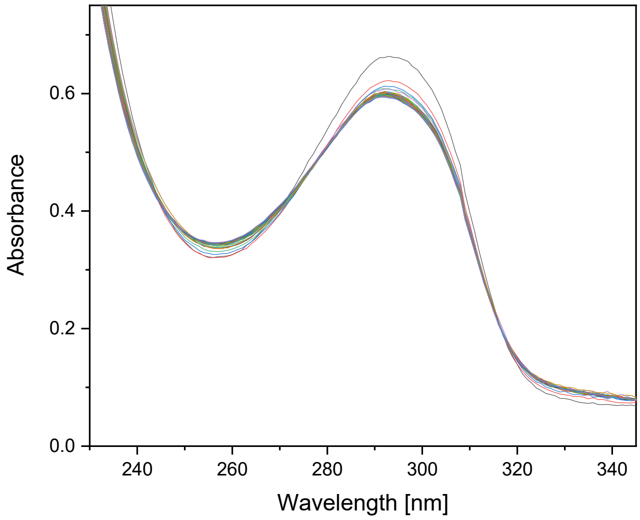


**Figure S9.** Change of UV/Vis spectrum of **(1)** upon incremental addition of methylphosphonic acid in aqueous bis-tris buffer at pH = 6.

Fitting parameters of bindfit fitter UV 1:1 of the range 280-310nm without the first 2 values:

| Parameter (bounds) | Optimised | Error | Initial |
|---|---|---|---|
| **K** ( 0 → ∞ ) | 5243.62 $M^{-1}$ | ± 0.9028 % | 100.00 $M^{-1}$ |

**Link to the calculation**: http://app.supramolecular.org/bindfit/view/67af73e9-2a41-4c9c-8c39-120f3f5f97d2

**Methylphosphonic acid to (1) measurement 2**

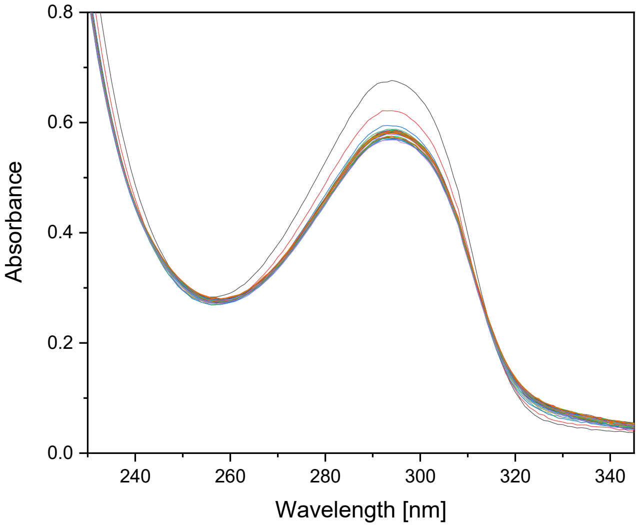


**Figure S10.** Change of UV/Vis spectrum of **(1)** upon incremental addition of methylphosphonic acid in aqueous bis-tris buffer at pH = 6.

Fitting parameters of bindfit fitter UV 1:1 of the range 280-310nm without the first 2 values:

| Parameter (bounds) | Optimised | Error | Initial |
|---|---|---|---|
| **K** ( 0 → ∞ ) | 3597.68 $M^{-1}$ | ± 0.6612 % | 100.00 $M^{-1}$ |

**Link to the calculation**: http://app.supramolecular.org/bindfit/view/34e4c5dc-b79c-4609-92f0-ecd7fefdcbf2

**Phenylphosphonic acid to (1) measurement 1**

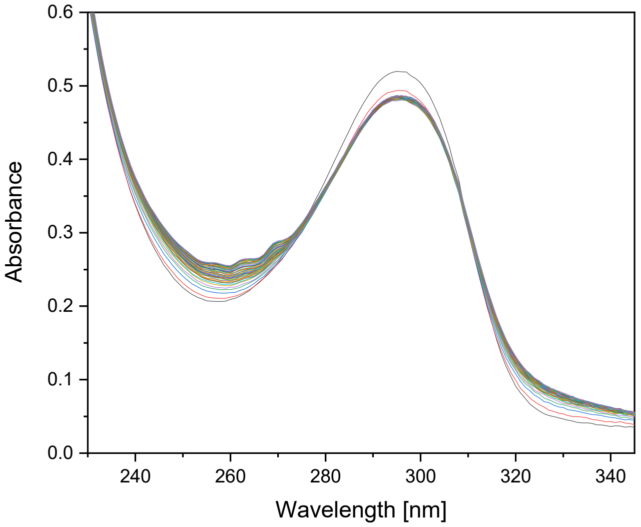


**Figure S11.** Change of UV/Vis spectrum of **(1)** upon incremental addition of phenylphosphonic acid in aqueous bis-tris buffer at pH = 6.

Fitting parameters of bindfit fitter UV 1:1 of the range 280-310nm without the first 2 values:

| Parameter (bounds) | Optimised | Error | Initial |
|---|---|---|---|
| **K** ( 0 → ∞ ) | 6424.26 $M^{-1}$ | ± 0.6855 % | 100.00 $M^{-1}$ |

**Link to the calculation**: http://app.supramolecular.org/bindfit/view/220eb341-0f09-4320-a548-518d15a57eab

**Phenylphosphonic acid to (1) measurement 2**

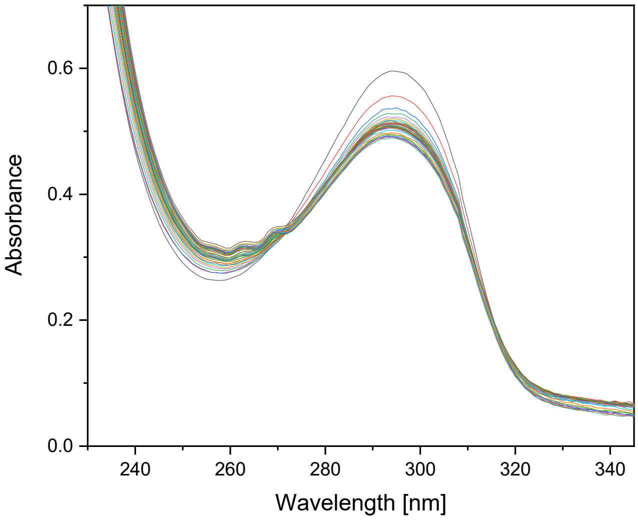


**Figure S12.** Change of UV/Vis spectrum of **(1)** upon incremental addition of phenylphosphonic acid in aqueous bis-tris buffer at pH = 6.

Fitting parameters of bindfit fitter UV 1:1 of the range 280-310nm without the first 2 values:

| Parameter (bounds) | Optimised | Error | Initial |
|---|---|---|---|
| **K** ( 0 → ∞ ) | 5500.85 $M^{-1}$ | ± 1.2636 % | 100.00 $M^{-1}$ |

**Link to the calculation**: http://app.supramolecular.org/bindfit/view/ed1bdf0d-fac7-452b-9142-f50f0f254f0a

**Methanesulfonic acid to (1) measurement 1**

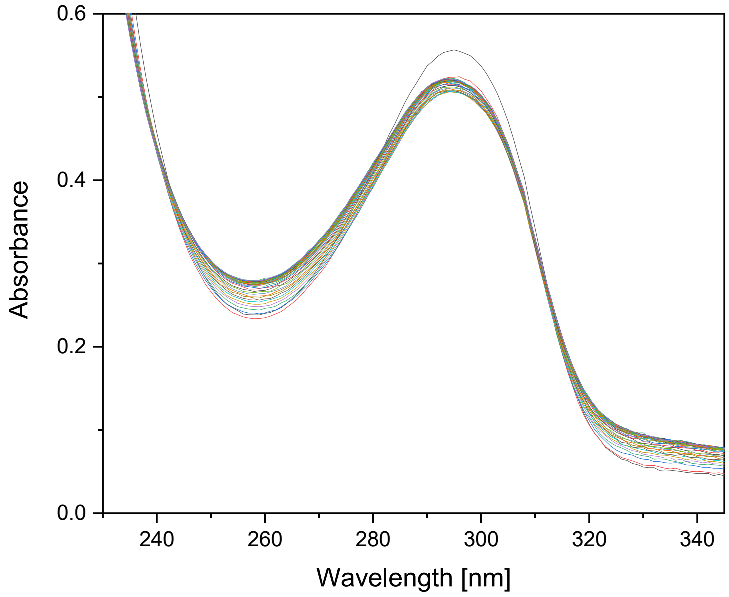


**Figure S13.** Change of UV/Vis spectrum of **(1)** upon incremental addition of methanesulfonic acid in aqueous bis-tris buffer at pH = 6.

Fitting parameters of bindfit fitter UV 1:1 of the range 280-310nm without the first 2 values:

| Parameter (bounds) | Optimised | Error | Initial |
|---|---|---|---|
| **K** ( 0 → ∞ ) | 11221.38 $M^{-1}$ | ± 6.4400 % | 100.00 $M^{-1}$ |

**Link to the calculation**: http://app.supramolecular.org/bindfit/view/3f01b7ab-a0da-4176-a61d-86afee3c344e

**Methanesulfonic acid to (1) measurement 2**

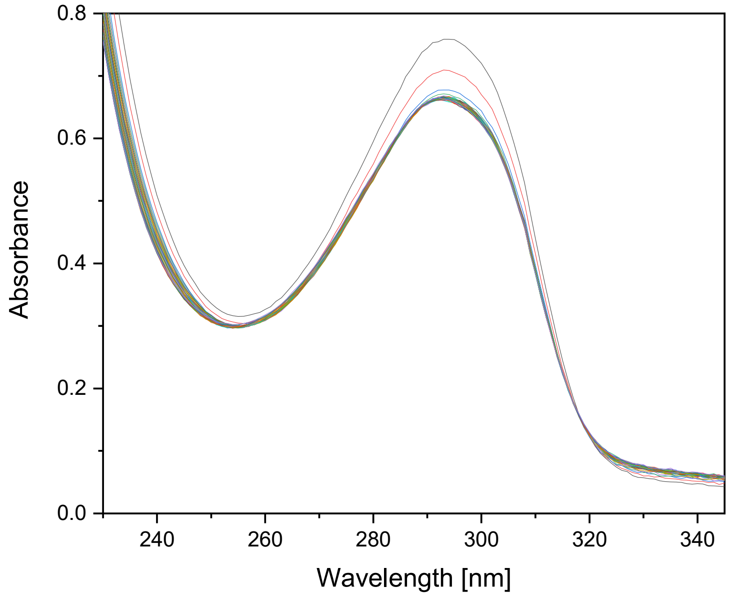


**Figure S14.** Change of UV/Vis spectrum of **(1)** upon incremental addition of methanesulfonic acid in aqueous bis-tris buffer at pH = 6.

Fitting parameters of bindfit fitter UV 1:1 of the range 280-310nmwithout the first 2 values:

| **Parameter (bounds)** | **Optimised** | **Error** | **Initial** |
|---|---|---|---|
| **K** ( 0 → ∞ ) | 11498.69 $M^{-1}$ | ± 1.2155 % | 100.00 $M^{-1}$ |

**Link to the calculation**: http://app.supramolecular.org/bindfit/view/0e04f9ae-41bd-44c4-b71d-ed65ff42dd2a

**Benzenesulfonic acid to (1) measurement 1**

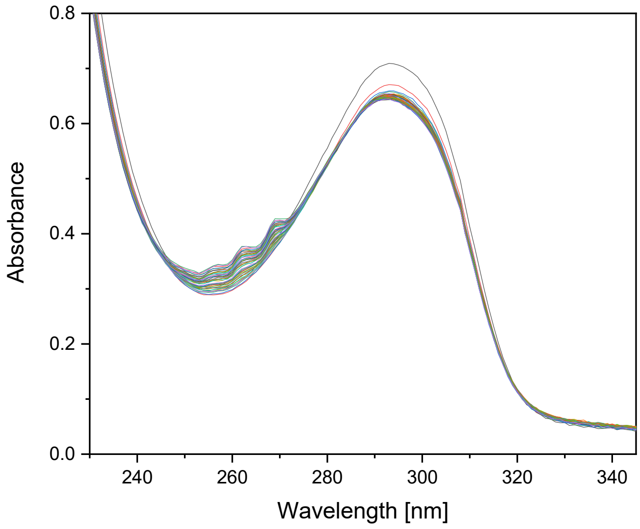


**Figure S15.** Change of UV/Vis spectrum of **(1)** upon incremental addition of benzenesulfonic acid in aqueous bis-tris buffer at pH = 6.

Fitting parameters of bindfit fitter UV 1:1 of the range 280-310nm without the first 2 values:

| Parameter (bounds) | Optimised | Error | Initial |
|---|---|---|---|
| **K** ( 0 → ∞ ) | 4249.24 $M^{-1}$ | ± 0.7679 % | 100.00 $M^{-1}$ |

**Link to the calculation**: http://app.supramolecular.org/bindfit/view/96782096-b01a-44fc-bd12-093aedfb4eaf

**Benzenesulfonic acid to (1) measurement 2**

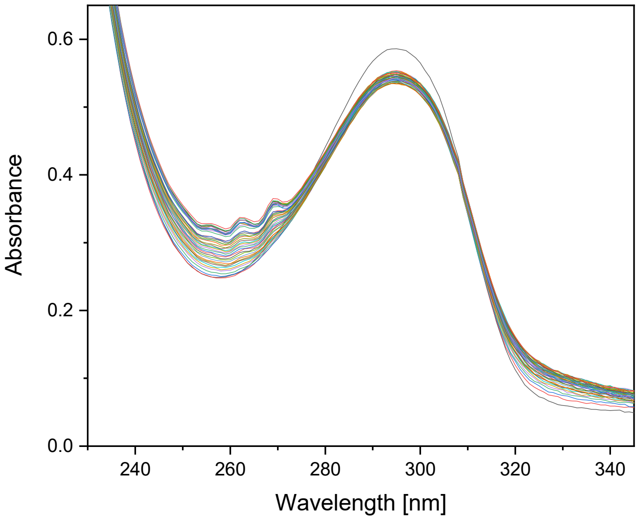


**Figure S16.** Change of UV/Vis spectrum of **(1)** upon incremental addition of benzenesulfonic acid in aqueous bis-tris buffer at pH = 6.

Fitting parameters of bindfit fitter UV 1:1 of the range 280-310nm without the first 2 values:

| Parameter (bounds) | Optimised | Error | Initial |
|---|---|---|---|
| **K** ( 0 → ∞ ) | 8267.55 $M^{-1}$ | ± 8.1898 % | 100.00 $M^{-1}$ |

**Link to the calculation**: http://app.supramolecular.org/bindfit/view/0c5dd66e-d4d8-4a36-b0c5-ec3ffb12916f

**NaOAc to (2) measurement 1**

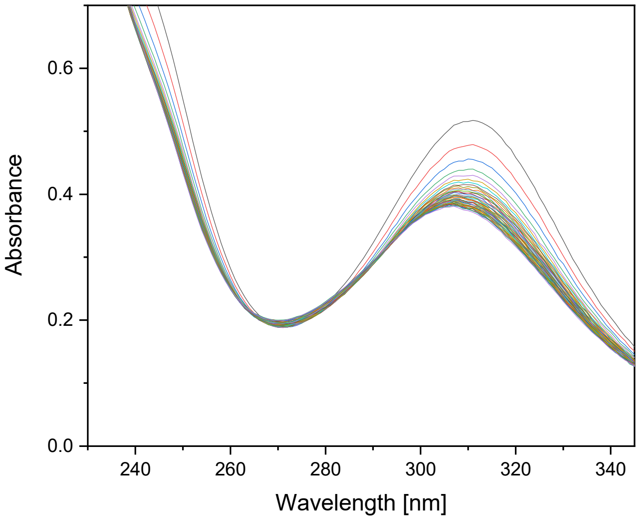


**Figure S17.** Change of UV/Vis spectrum of **(2)** upon incremental addition of sodium acetate in aqueous bis-tris buffer at pH = 6.

Fitting parameters of bindfit fitter UV 1:1 of the range 290-330nm without the first 2 values:

| Parameter (bounds) | Optimised | Error | Initial |
|---|---|---|---|
| **K** ( 0 → ∞ ) | 16806.93 $M^{-1}$ | ± 0.7256 % | 100.00 $M^{-1}$ |

**Link to the calculation**: http://app.supramolecular.org/bindfit/view/8aac78ab-b933-4df8-8fe2-b2b9120acaf0

**NaOAc to (2) measurement 2**

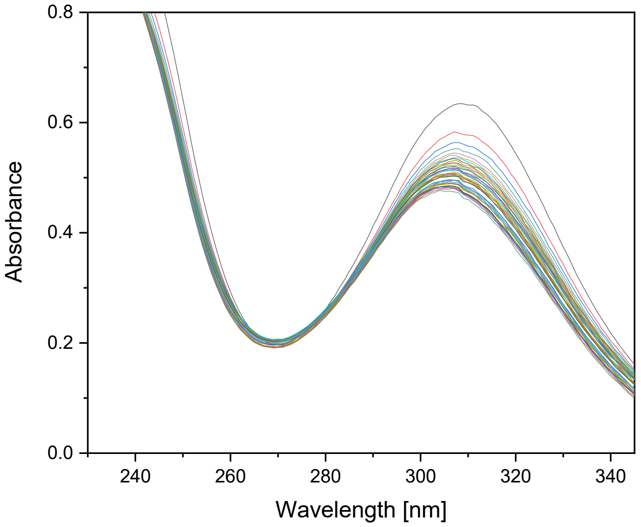


**Figure S18.** Change of UV/Vis spectrum of **(2)** upon incremental addition of sodium acetate in aqueous bis-tris buffer at pH = 6.

Fitting parameters of bindfit fitter UV 1:1 of the range 290-330nm without the first 2 values:

| Parameter (bounds) | Optimised | Error | Initial |
|---|---|---|---|
| **K** ( 0 → ∞ ) | 15921.06 $M^{-1}$ | ± 1.2303 % | 100.00 $M^{-1}$ |

**Link to the calculation**: http://app.supramolecular.org/bindfit/view/b467372c-8665-42a7-a6bb-c86b618871e8

**Benzoic acid to (2) measurement 1**

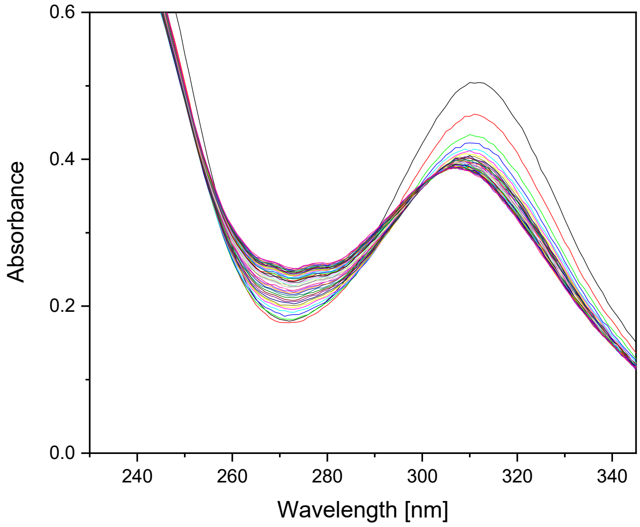


**Figure S19.** Change of UV/Vis spectrum of **(2)** upon incremental addition of benzoic acid in aqueous bis-tris buffer at pH = 6.

Fitting parameters of bindfit fitter UV 1:1 of the range 290-330nm without the first 2 values:

| Parameter (bounds) | Optimised | Error | Initial |
|---|---|---|---|
| **K** ( 0 → ∞ ) | 18167.90 $M^{-1}$ | ± 1.0290 % | 100.00 $M^{-1}$ |

**Link to the calculation**: http://app.supramolecular.org/bindfit/view/09974cf9-1d2a-4dde-b0f0-601ff71f8b67

**Benzoic acid to (2) measurement 2**

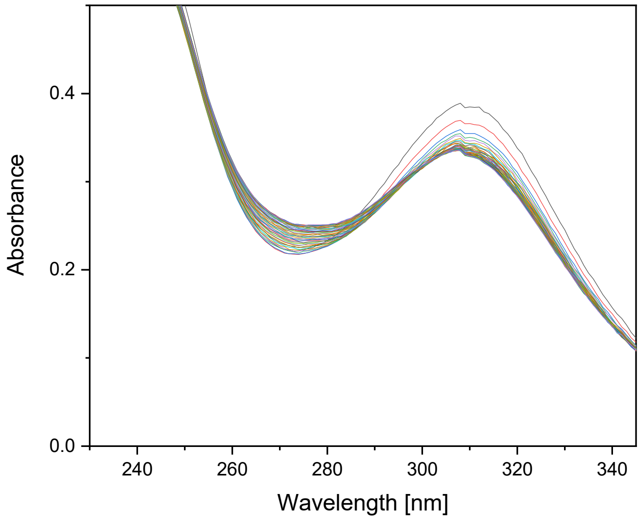


**Figure S20.** Change of UV/Vis spectrum of **(2)** upon incremental addition of benzoic acid in aqueous bis-tris buffer at pH = 6.

Fitting parameters of bindfit fitter UV 1:1 of the range 290-330nm without the first 2 values:

| Parameter (bounds) | Optimised | Error | Initial |
|---|---|---|---|
| **K** ( 0 → ∞ ) | 16509.29 $M^{-1}$ | ± 1.0505 % | 100.00 $M^{-1}$ |

**Link to the calculation**: http://app.supramolecular.org/bindfit/view/ac8729f4-2585-44b7-8638-339c66cd842d

**Methylphosphonic acid to (2) measurement 1**

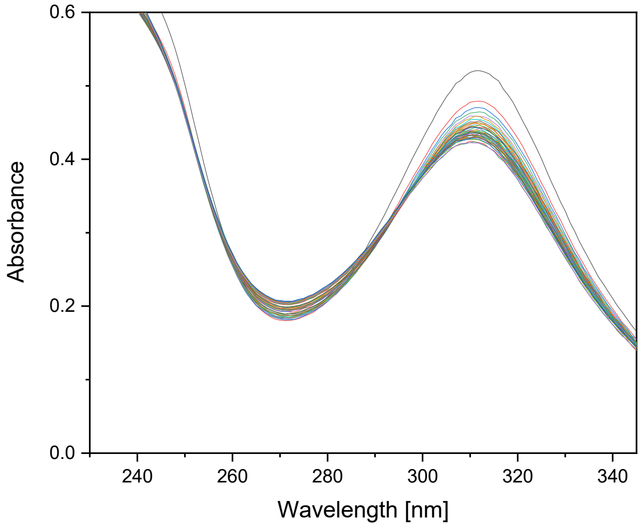


**Figure S21.** Change of UV/Vis spectrum of **(2)** upon incremental addition of methylphosphonic acid in aqueous bis-tris buffer at pH = 6.

Fitting parameters of bindfit fitter UV 1:1 of the range 290-330nm without the first 2 values:

| Parameter (bounds) | Optimised | Error | Initial |
|---|---|---|---|
| **K** ( 0 → ∞ ) | 7237.86 $M^{-1}$ | ± 0.7322 % | 100.00 $M^{-1}$ |

**Link to the calculation**: http://app.supramolecular.org/bindfit/view/6f2744d8-401a-4d36-b71b-76523b06e952

**Methylphosphonic acid to (2) measurement 2**

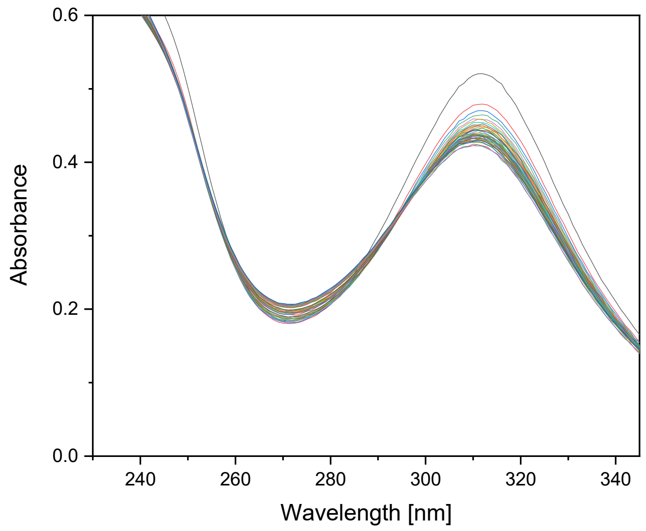


**Figure S22.** Change of UV/Vis spectrum of **(2)** upon incremental addition of methylphosphonic acid in aqueous bis-tris buffer at pH = 6.

Fitting parameters of bindfit fitter UV 1:1 of the range 290-330nm without the first 2 values:

| **Parameter (bounds)** | **Optimised** | **Error** | **Initial** |
|---|---|---|---|
| **K** ( 0 → ∞ ) | 8483.17 $M^{-1}$ | ± 1.4333 % | 100.00 $M^{-1}$ |

**Link to the calculation:** http://app.supramolecular.org/bindfit/view/108c802a-d560-4857-9957-c64872062e40

**Phenylphosphonic acid to (2) measurement 1**

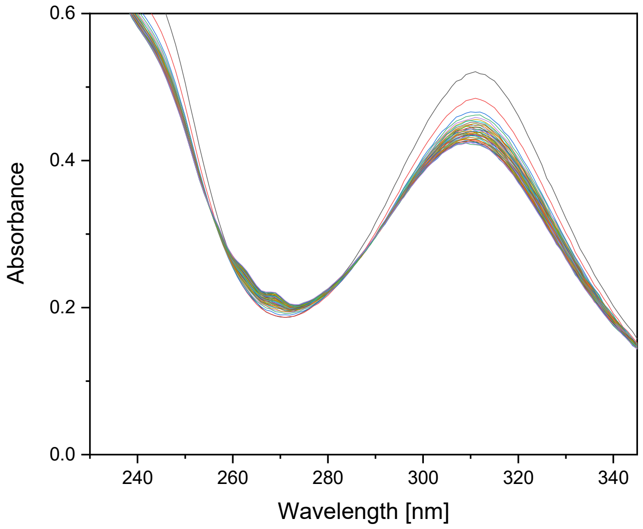


**Figure S23.** Change of UV/Vis spectrum of **(2)** upon incremental addition of phenylphosphonic acid in aqueous bis-tris buffer at pH = 6.

Fitting parameters of bindfit fitter UV 1:1 of the range 290-330nm without the first 2 values:

| Parameter (bounds) | Optimised | Error | Initial |
|---|---|---|---|
| **K** ( 0 → ∞ ) | 5170.38 $M^{-1}$ | ± 0.4694 % | 100.00 $M^{-1}$ |

**Link to the calculation**: http://app.supramolecular.org/bindfit/view/25343535-44c9-415c-9a5d-c1b7240bf11c

**Phenylphosphonic acid to (2) measurement 2**

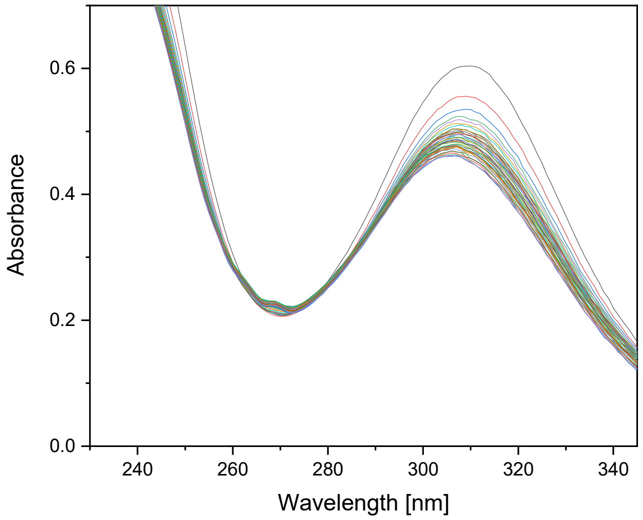


**Figure S24.** Change of UV/Vis spectrum of **(2)** upon incremental addition of phenylphosphonic acid in aqueous bis-tris buffer at pH = 6.

Fitting parameters of bindfit fitter UV 1:1 of the range 290-330nm without the first 2 values:

| Parameter (bounds) | Optimised | Error | Initial |
|---|---|---|---|
| **K** ( 0 → ∞ ) | 5425.76 $M^{-1}$ | ± 0.5890 % | 100.00 $M^{-1}$ |

**Link to the calculation**: http://app.supramolecular.org/bindfit/view/6e027349-81ce-4cf9-866d-ef6826681ab0

**Methanesulfonic acid to (2) measurement 1**

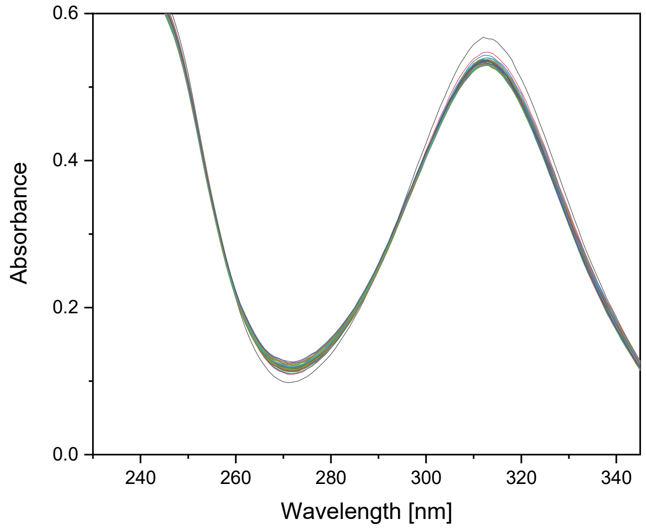


**Figure S25.** Change of UV/Vis spectrum of (5) upon incremental addition of methanesulfonic acid in aqueous bis-tris buffer at pH = 6.

Fitting parameters of bindfit fitter UV 1:1 of the range 290-330nm without the first 2 values:

| Parameter (bounds) | Optimised | Error | Initial |
|---|---|---|---|
| **K** ( 0 → ∞ ) | 2145.35 $M^{-1}$ | ± 0.7685 % | 100.00 $M^{-1}$ |

**Link to the calculation**: http://app.supramolecular.org/bindfit/view/b39a7430-fc4d-4c57-b2e8-48513ff0f6fe

**Methanesulfonic acid to (2) measurement 2**

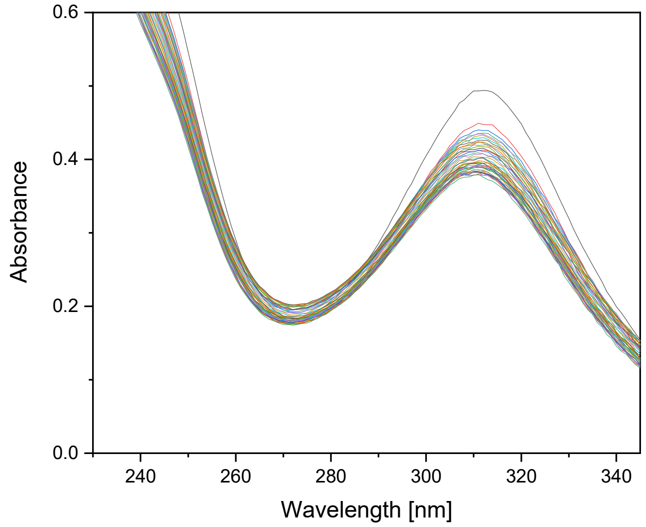


**Figure S26.** Change of UV/Vis spectrum of **(2)** upon incremental addition of methanesulfonic acid in aqueous bis-tris buffer at pH = 6.

Fitting parameters of bindfit fitter UV 1:1 of the range 290-330nm without the first 2 values:

| Parameter (bounds) | Optimised | Error | Initial |
|---|---|---|---|
| **K** ( 0 → ∞ ) | 1667.95 $M^{-1}$ | ± 0.5516 % | 100.00 $M^{-1}$ |

**Link to the calculation**: http://app.supramolecular.org/bindfit/view/f4a96af5-ad49-4f7b-9ad4-4d4944ba9702

**Benzenesulfonic acid to (2) measurement 1**

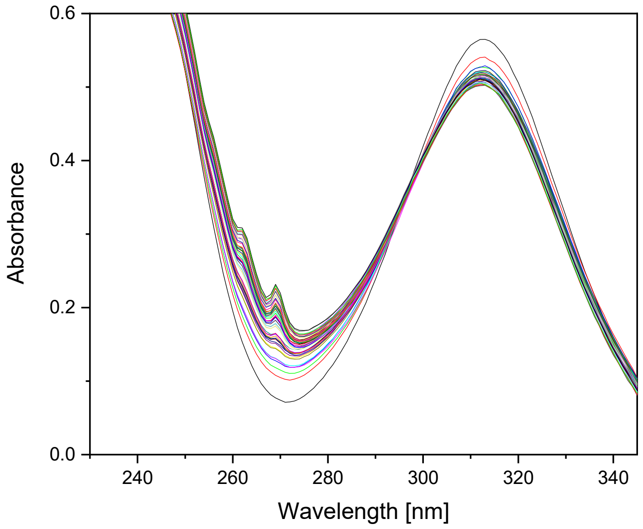


**Figure S27.** Change of UV/Vis spectrum of **(2)** upon incremental addition of benzenesulfonic acid in aqueous bis-tris buffer at pH = 6.

Fitting parameters of bindfit fitter UV 1:1 of the range 290-330nm without the first 2 values:

| Parameter (bounds) | Optimised | Error | Initial |
|---|---|---|---|
| **K** ( 0 → ∞ ) | 4034.63 $M^{-1}$ | ± 0.6311 % | 100.00 $M^{-1}$ |

**Link to the calculation**: http://app.supramolecular.org/bindfit/view/8658d4dd-0be1-492a-acc1-5c1a9030f428

**Benzenesulfonic acid to (2) measurement 2**

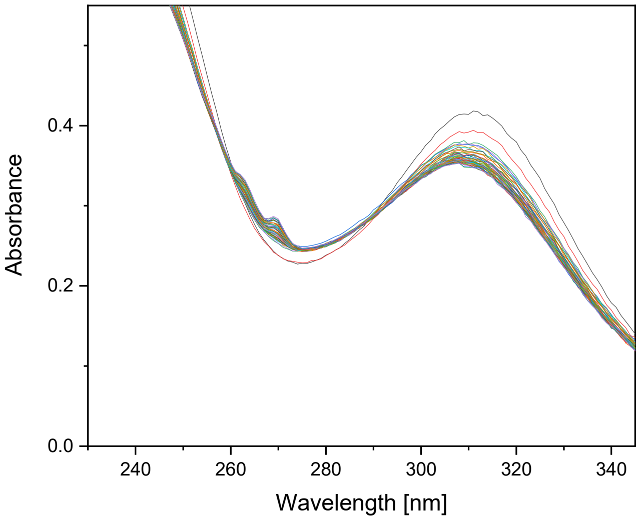


**Figure S28.** Change of UV/Vis spectrum of **(2)** upon incremental addition of benzenesulfonic acid in aqueous bis-tris buffer at pH = 6.

Fitting parameters of bindfit fitter UV 1:1 of the range 290-330nm without the first 2 values:

| Parameter (bounds) | Optimised | Error | Initial |
|---|---|---|---|
| **K** ( 0 → ∞ ) | 3094.79 $M^{-1}$ | ± 0.6466 % | 100.00 $M^{-1}$ |

**Link to the calculation**: http://app.supramolecular.org/bindfit/view/7b0ea17f-d069-4f43-8984-a40e8d647c5f

**NaOAc to (3) measurement 1**

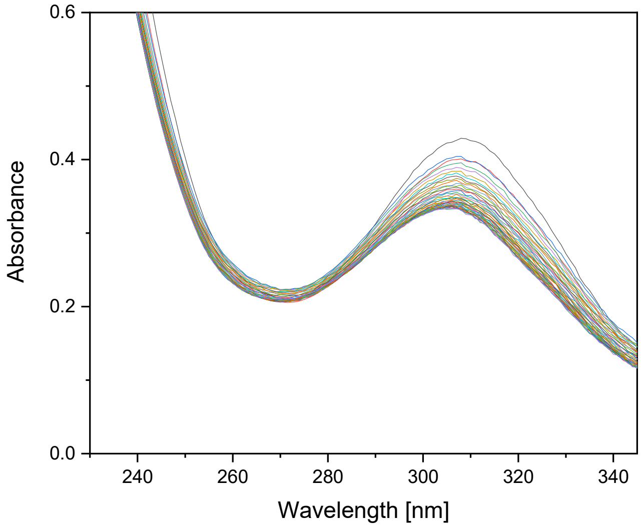


**Figure S29.** Change of UV/Vis spectrum of **(3)** upon incremental addition of NaOAc in aqueous bis-tris buffer at pH = 6.

Fitting parameters of bindfit fitter UV 1:1 of the range 290-330nm without the first 2 values:

| Parameter (bounds) | Optimised | Error | Initial |
|---|---|---|---|
| **K** ( 0 → ∞ ) | 9306.29 $M^{-1}$ | ± 0.4696 % | 100.00 $M^{-1}$ |

**Link to the calculation**: http://app.supramolecular.org/bindfit/view/8f55c5ee-cefe-4f15-9a32-f745ee0c0d30

**NaOAc to (3) measurement 2**

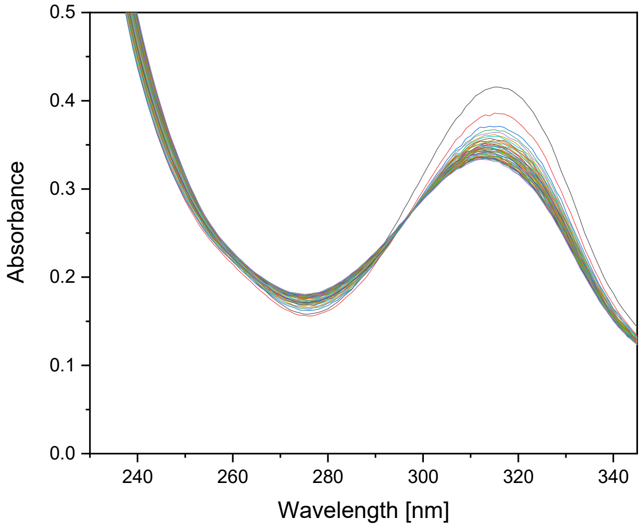


**Figure S30.** Change of UV/Vis spectrum of **(3)** upon incremental addition of NaOAc in aqueous bis-tris buffer at pH = 6.

Fitting parameters of bindfit fitter UV 1:1 of the range 290-330nm without the first 2 values:

| **Parameter (bounds)** | **Optimised** | **Error** | **Initial** |
|---|---|---|---|
| **K** ( 0 → ∞ ) | 5927.25 $M^{-1}$ | ± 0.3995 % | 100.00 $M^{-1}$ |

**Link to the calculation**: http://app.supramolecular.org/bindfit/view/27c9df48-ab7b-48a4-9a8a-d962feb2b3a8

**Benzoic acid to (3) measurement 1**

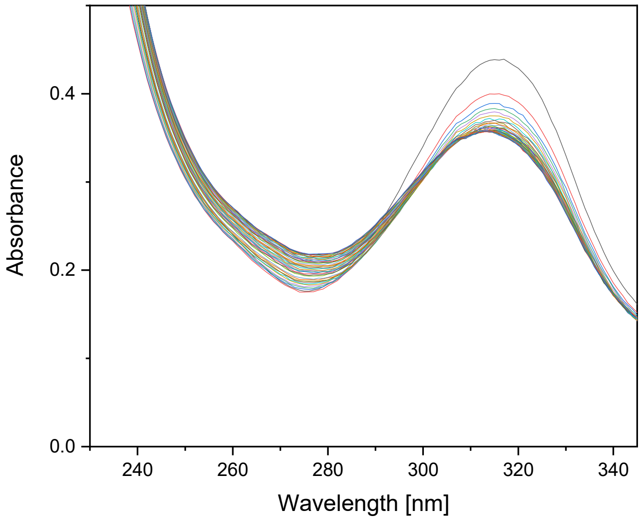


**Figure S31.** Change of UV/Vis spectrum of **(3)** upon incremental addition of benzoic acid in aqueous bis-tris buffer at pH = 6.

Fitting parameters of bindfit fitter UV 1:1 of the range 290-330nm without the first 2 values:

| **Parameter (bounds)** | **Optimised** | **Error** | **Initial** |
|---|---|---|---|
| **K** ( 0 → ∞ ) | 1649.22 $M^{-1}$ | ± 1.9488 % | 100.00 $M^{-1}$ |

**Link to the calculation**: http://app.supramolecular.org/bindfit/view/759d1fe3-812e-4ecd-ae31-a97149500865

**Benzoic acid to (3) measurement 2**

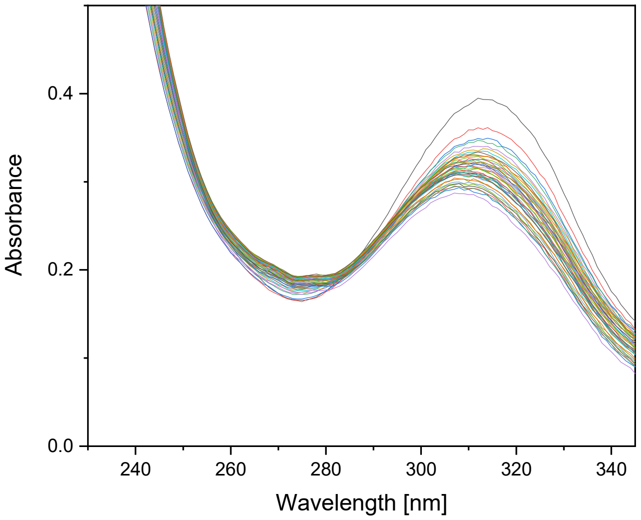


**Figure S32.** Change of UV/Vis spectrum of **(3)** upon incremental addition of benzoic acid in aqueous bis-tris buffer at pH = 6.

Fitting parameters of bindfit fitter UV 1:1 of the range 290-330nm without the first 2 values:

| **Parameter (bounds)** | **Optimised** | **Error** | **Initial** |
|---|---|---|---|
| **K** ( 0 → ∞ ) | 1102.65 $M^{-1}$ | ± 0.5111 % | 100.00 $M^{-1}$ |

**Link to the calculation**: http://app.supramolecular.org/bindfit/view/287b534f-560e-479f-9d45-435b4a8b116a

**Methylphosphonic acid to (3) measurement 1**

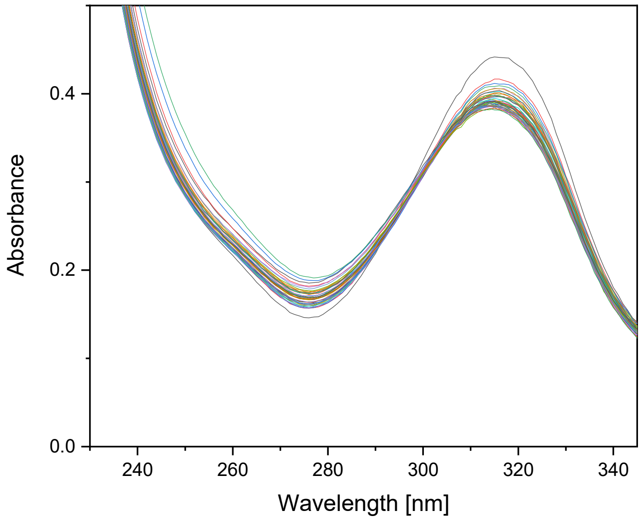


**Figure S33.** Change of UV/Vis spectrum of **(3)** upon incremental addition of methylphosphonic acid in aqueous bis-tris buffer at pH = 6.

Fitting parameters of bindfit fitter UV 1:1 of the range 290-330nm without the first 2 values:

| Parameter (bounds) | Optimised | Error | Initial |
|---|---|---|---|
| **K** ( 0 → ∞ ) | 5599.08 $M^{-1}$ | ± 0.9651 % | 100.00 $M^{-1}$ |

**Link to the calculation**: http://app.supramolecular.org/bindfit/view/5de27a95-c80e-4098-92c9-db03232be021

**Methylphosphonic acid to (3) measurement 2**

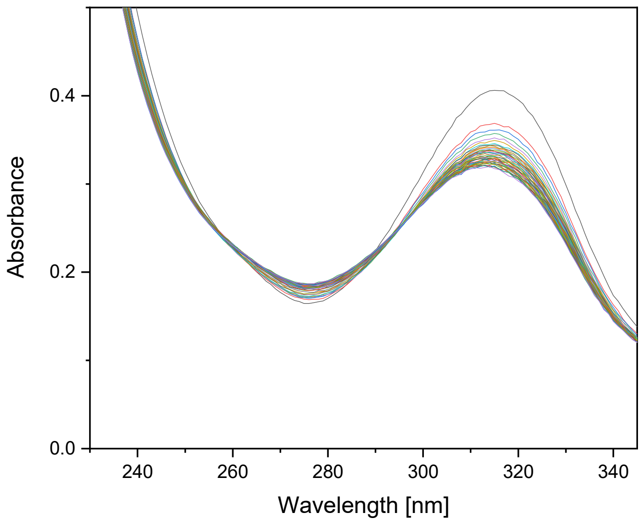


**Figure S34.** Change of UV/Vis spectrum of **(3)** upon incremental addition of methylphosphonic acid in aqueous bis-tris buffer at pH = 6.

Fitting parameters of bindfit fitter UV 1:1 of the range 290-330nm without the first 2 values:

| **Parameter (bounds)** | **Optimised** | **Error** | **Initial** |
|---|---|---|---|
| **K** ( 0 → ∞ ) | 8238.08 $M^{-1}$ | ± 0.4741 % | 100.00 $M^{-1}$ |

**Link to the calculation**: http://app.supramolecular.org/bindfit/view/65b1c134-c2a4-442f-aac6-9606b442c8ea

**Phenylphosphonic acid to (3) measurement 1**

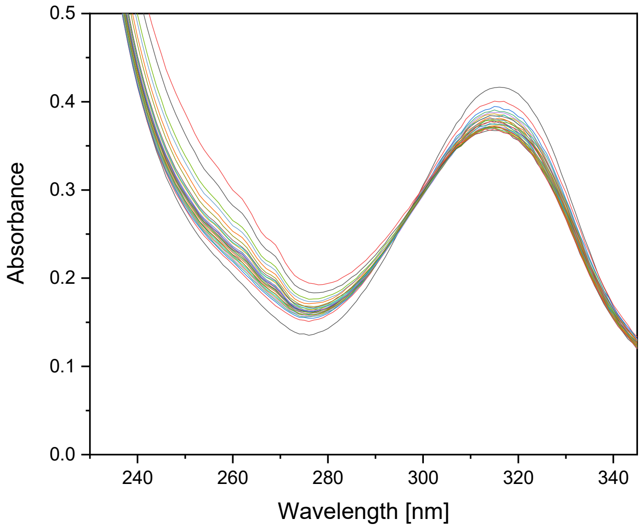


**Figure S35.** Change of UV/Vis spectrum of **(3)** upon incremental addition of phenylphosphonic acid in aqueous bis-tris buffer at pH = 6.

Fitting parameters of bindfit fitter UV 1:1 of the range 290-330nm without the first 2 values:

| **Parameter (bounds)** | **Optimised** | **Error** | **Initial** |
|---|---|---|---|
| **K** ( 0 → ∞ ) | 3849.89 $M^{-1}$ | ± 0.7873 % | 100.00 $M^{-1}$ |

**Link to the calculation**: http://app.supramolecular.org/bindfit/view/afd53d4d-9271-4f90-97a9-de7fcfa65f9c

**Phenylphosphonic acid to (3) measurement 2**

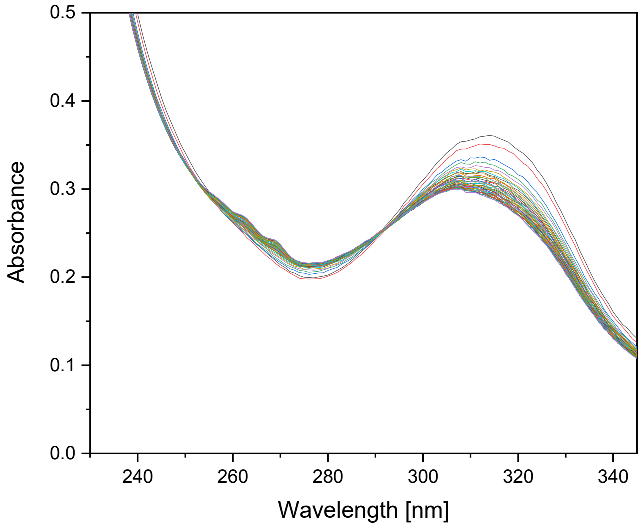


**Figure S36.** Change of UV/Vis spectrum of **(3)** upon incremental addition of phenylphosphonic acid in aqueous bis-tris buffer at pH = 6.

Fitting parameters of bindfit fitter UV 1:1 of the range 290-330nm without the first 2 values:

| Parameter (bounds) | Optimised | Error | Initial |
|---|---|---|---|
| **K** ( 0 → ∞ ) | 9273.96 $M^{-1}$ | ± 0.4405 % | 100.00 $M^{-1}$ |

**Link to the calculation**:http://app.supramolecular.org/bindfit/view/68d17e82-8b99-4351-bb86-4e3cb7acfa49

**Methanesulfonic acid to (3) measurement 1**

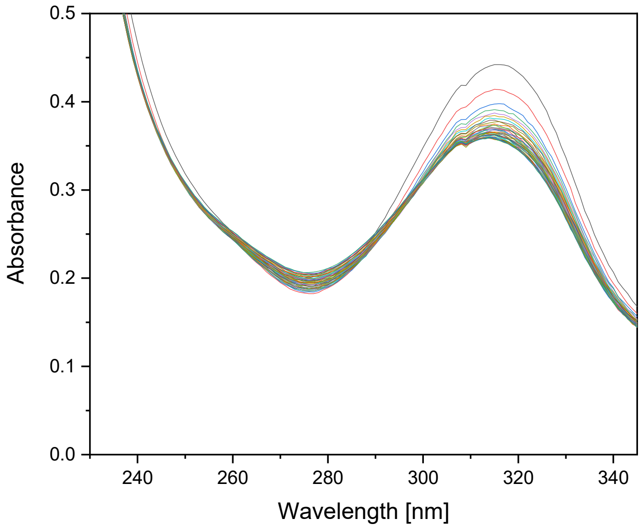


**Figure S37.** Change of UV/Vis spectrum of **(3)** upon incremental addition of methanesulfonic acid in aqueous bis-tris buffer at pH = 6.

Fitting parameters of bindfit fitter UV 1:1 of the range 290-330nm without the first 2 values:

| Parameter (bounds) | Optimised | Error | Initial |
|---|---|---|---|
| **K** ( 0 → ∞ ) | 13335.27 $M^{-1}$ | ± 0.7739 % | 100.00 $M^{-1}$ |

**Link to the calculation**: http://app.supramolecular.org/bindfit/view/a7e8ce7b-70dc-4ab9-be43-5c50600acd27

**Methanesulfonic acid to (3) measurement 2**

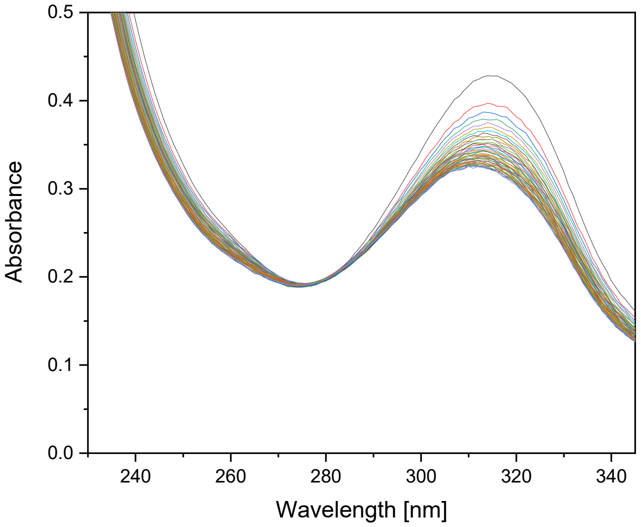


**Figure S38.** Change of UV/Vis spectrum of **(3)** upon incremental addition of methanesulfonic acid in aqueous bis-tris buffer at pH = 6.

Fitting parameters of bindfit fitter UV 1:1 of the range 290-330nm without the first 2 values:

| Parameter (bounds) | Optimised | Error | Initial |
|---|---|---|---|
| **K** ( 0 → ∞ ) | 8578.39 $M^{-1}$ | ± 0.4231 % | 100.00 $M^{-1}$ |

**Link to the calculation**: http://app.supramolecular.org/bindfit/view/0752603e-cc76-4daf-a407-ac64588656ee

**Benzenesulfonic acid to (3) measurement 1**

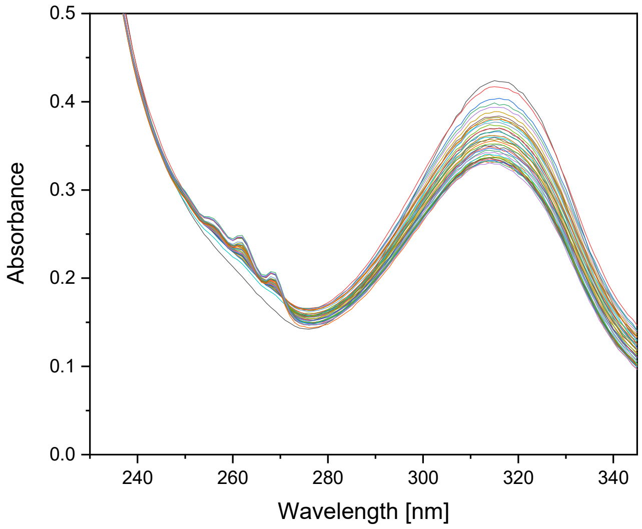


**Figure S39.** Change of UV/Vis spectrum of **(3)** upon incremental addition of benzenesulfonic acid in aqueous bis-tris buffer at pH = 6.

Fitting parameters of bindfit fitter UV 1:1 of the range 290-330nm without the first 2 values:

| Parameter (bounds) | Optimised | Error | Initial |
|---|---|---|---|
| **K** ( 0 → ∞ ) | 3081.83 $M^{-1}$ | ± 0.3804 % | 100.00 $M^{-1}$ |

**Link to the calculation**: http://app.supramolecular.org/bindfit/view/41fac8ee-5a87-4c0a-9291-babcc0f4974c

**Benzenesulfonic acid to (3) measurement 2**

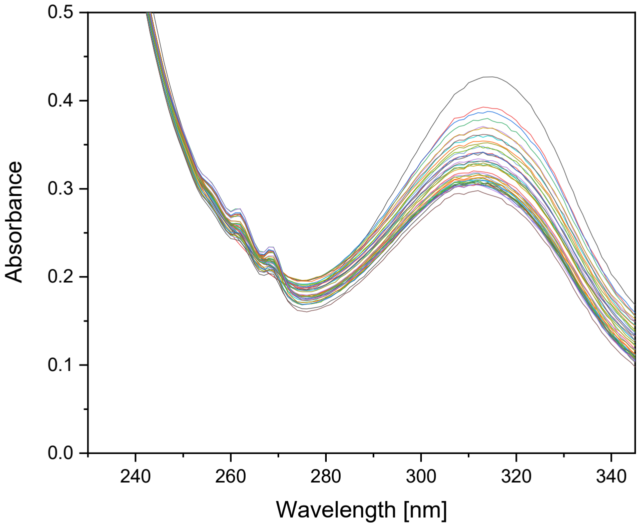


**Figure S40.** Change of UV/Vis spectrum of **(3)** upon incremental addition of benzenesulfonic acid in aqueous bis-tris buffer at pH = 6.

Fitting parameters of bindfit fitter UV 1:1 of the range 290-330nm without the first 2 values:

| Parameter (bounds) | Optimised | Error | Initial |
|---|---|---|---|
| **K** ( 0 → ∞ ) | 7486.78 $M^{-1}$ | ± 0.8539 % | 100.00 $M^{-1}$ |

**Link to the calculation**: http://app.supramolecular.org/bindfit/view/4527c88f-2c44-42ea-868d-bdaf031aedf2

**Table S3.** Association constants ($K_{ass}$) and errors as calculated with http://supramolecular.org for the 1:1 complexes formed between **1**, **2** and **3**and various substrates in bis-tris buffer 6mM, pH = 6.

| | **(1)** | | **(2)** | | **(3)** | |
|---|---|---|---|---|---|---|
| | $K_{ass}$ [$M^{-1}$] | error [%] | $K_{ass}$ [$M^{-1}$] | error[%] | $K_{ass}$ [$M^{-1}$] | error [%] |
| NaOAc[(a)] | 16760.58 | 1.04 | 15921.06 | 1.23 | 9306.29 | 0.47 |
| | 19201.36 | 1.71 | 16806.93 | 0.73 | 5927.25 | 0.40 |
| Benz[(a)] | 7022.15 | 3.69 | 18167.90 | 1.03 | 1649.22 | 1.95 |
| | 4432.12 | 2.84 | 16509.29 | 1.05 | 1102.65 | 0.51 |
| Mphos[(a)] | 5243.62 | 0.90 | 7237.86 | 0.73 | 5599.08 | 0.97 |
| | 3597.68 | 0.66 | 8483.17 | 1.43 | 8238.08 | 0.47 |
| PPhos[(a)] | 6424.26 | 0.69 | 5170.38 | 0.47 | 3849.89 | 0.79 |
| | 5500.85 | 1.26 | 5425.76 | 0.59 | 9273.96 | 0.44 |
| MSulf[(a)] | 11221.38 | 6.44 | 2145.35 | 0.77 | 13335.27 | 0.77 |
| | 11498.69 | 1.22 | 1667.95 | 0.55 | 8578.39 | 0.42 |
| BSulf[(a)] | 8267.55 | 8.19 | 4034.63 | 0.63 | 3081.83 | 0.38 |
| | 4249.24 | 0.77 | 3094.79 | 0.65 | 7486.78 | 0.85 |

(a) NaOAc: sodiumacetate, Benz: benzoic acid, MPhos: methylphosphonic acid, PPhos: phenylphosphonic acid, MSulf: methanesulfonic acid, BSulf: benzenesulfonic acid

## 5. Job plots

Job plots were performed using a modified procedure known from literature [51].

Buffered solutions of the substrates (sodium acetate, benzoic acid, methylphosphonic acid, phenylphosphonic acid, methanesulfonic acid, benzenesulfonic acid) (c = 6.0 x $10^{-5}$ M) and the receptors (**1, 2, 3**) (c = 6.0 x $10^{-5}$ M) in bis-tris buffer, 6 mM, pH = 6, were prepared. UV/Vis measurements (350-230 nm) of the maximal absorption of the receptors (λ = 300 nm for **1**, λ = 314 nm for **2**, λ = 320 nm for **3**) were recorded, starting with 800 µL of a solution that contained only the receptor. Defined amounts of solvent were then removed from the cuvette and replaced with an equal amount of substrate solution (80.00 µL, 88.89 µL, 70.0 µL, 65.75 µL, 83.58 µL, 66.67 µL, 72.73 µL, 80.00 µL, 88.89 µL, 120.00 µL, 164.71 µL, 207.41 µL, 400.00 µL). This ensures that the total concentration [C] = [R] + [S] (R = receptor, S = substrate) remains constant and mole fractions of: 0, 0.1, 0.2, 0.27, 0.33, 0.4, 0.45, 0.5, 0.55, 0.6, 0.66, 0.73, 0.8, 0.9 and 1. A final data point of pure substrate was recorded. All data was recorded in triplicate and for further evaluation the average was formed. Also a blank measurement of the buffer was subtracted from the spectra.

The data was evaluated by plotting the molar fraction (χ) against $\Delta A = |A_{exp} - A_R - A_S|$. $A_{exp}$ is the absorption of a given mixture, $A_R$ and $A_S$ are the absorptions of receptor and substrate at the corresponding concentration at the wavelength specific to the receptor (λ = 300 nm for 1, λ = 314 nm for 2, λ = 320 nm for 3). $A_R$ was calculated by using the absorbance at the first data point of the mentioned measurement (just receptor is present) to calculate the absorbance at each data points by using the known receptor concentrations. $A_S$ was determined experimentally by conducting a blank measurement without the receptor as described above. The data points were fitted with a polynomial fit of the 2nd degree and the maximum identified. A maximum at a molar ration of 0.5 indicates a 1:1 complex stoichiometry [7].

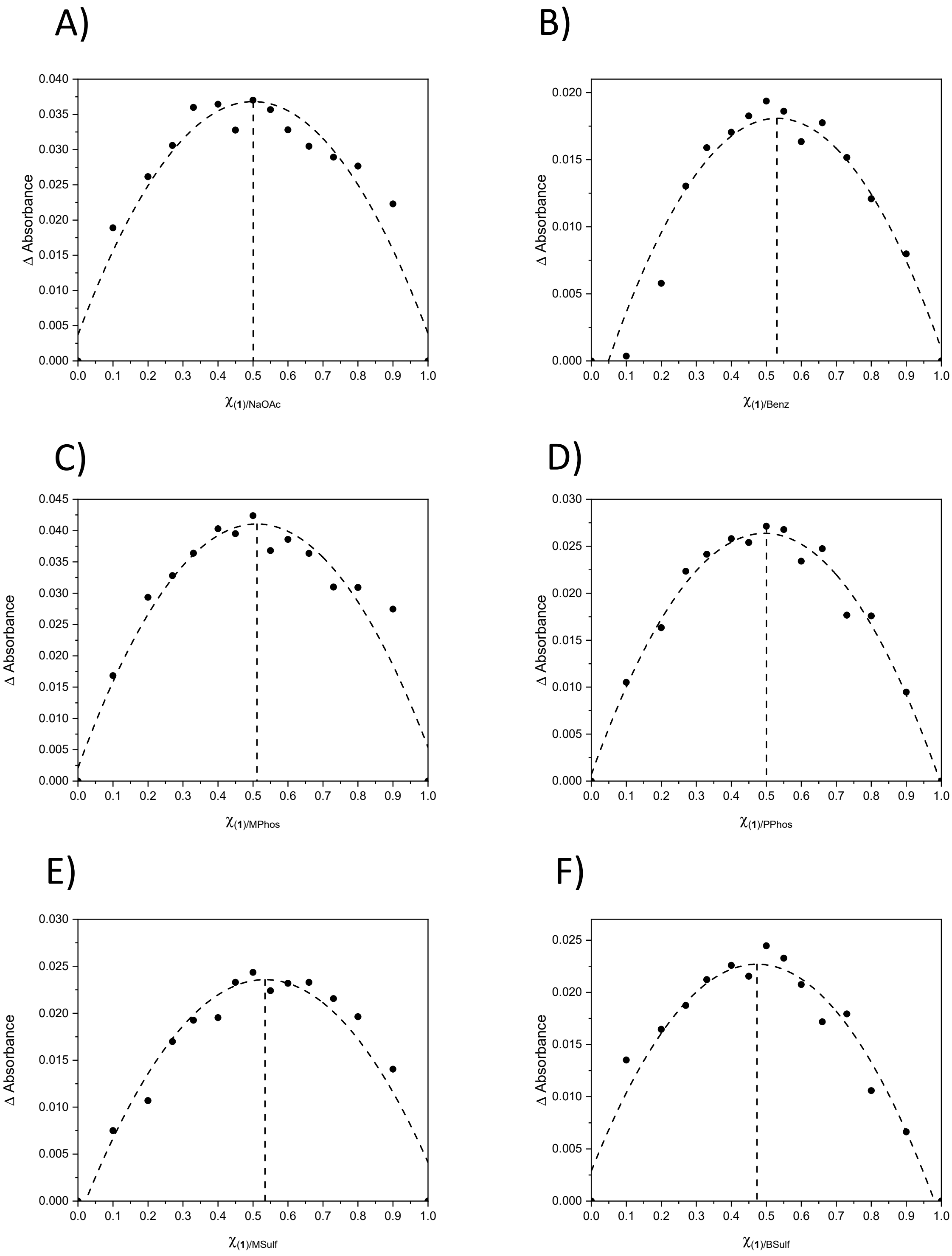


**Figure S41.** Job plots of the complex formation between receptor **1** and A) sodium acetate, B) benzoic acid, C) methylphosphonic acid, D) phenylphosphonic acid, E) methanesulfonic acid, F) benzenesulfonic acid, which confirm a 1:1 complex stoichiometry in all cases.

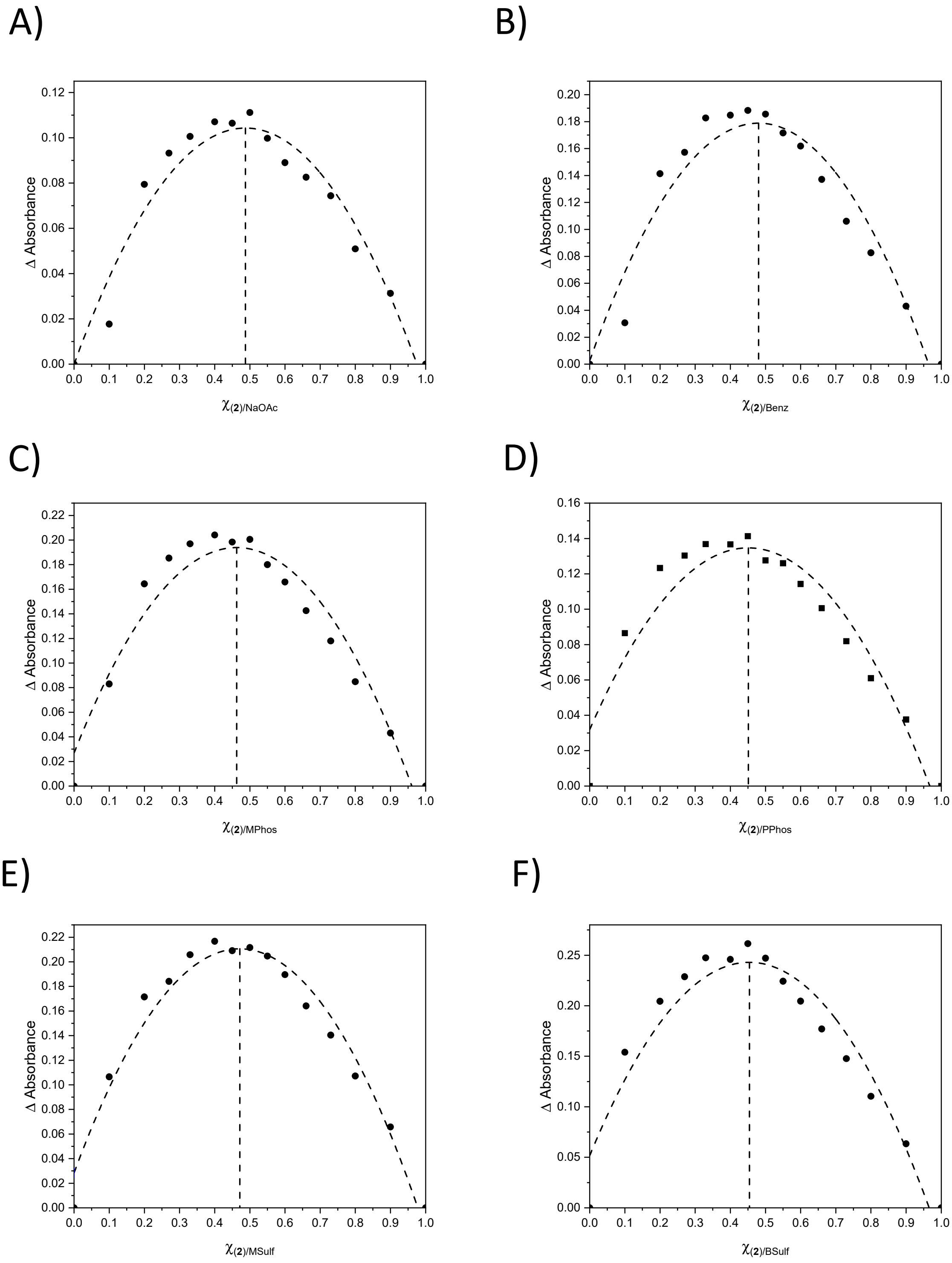


**Figure S42.** Job plots of the complex formation between receptor **2** and A) sodium acetate, B) benzoic acid, C) methylphosphonic acid, D) phenylphosphonic acid, E) methanesulfonic acid, F) benzenesulfonic acid, which confirm a 1:1 complex stoichiometry in all cases.

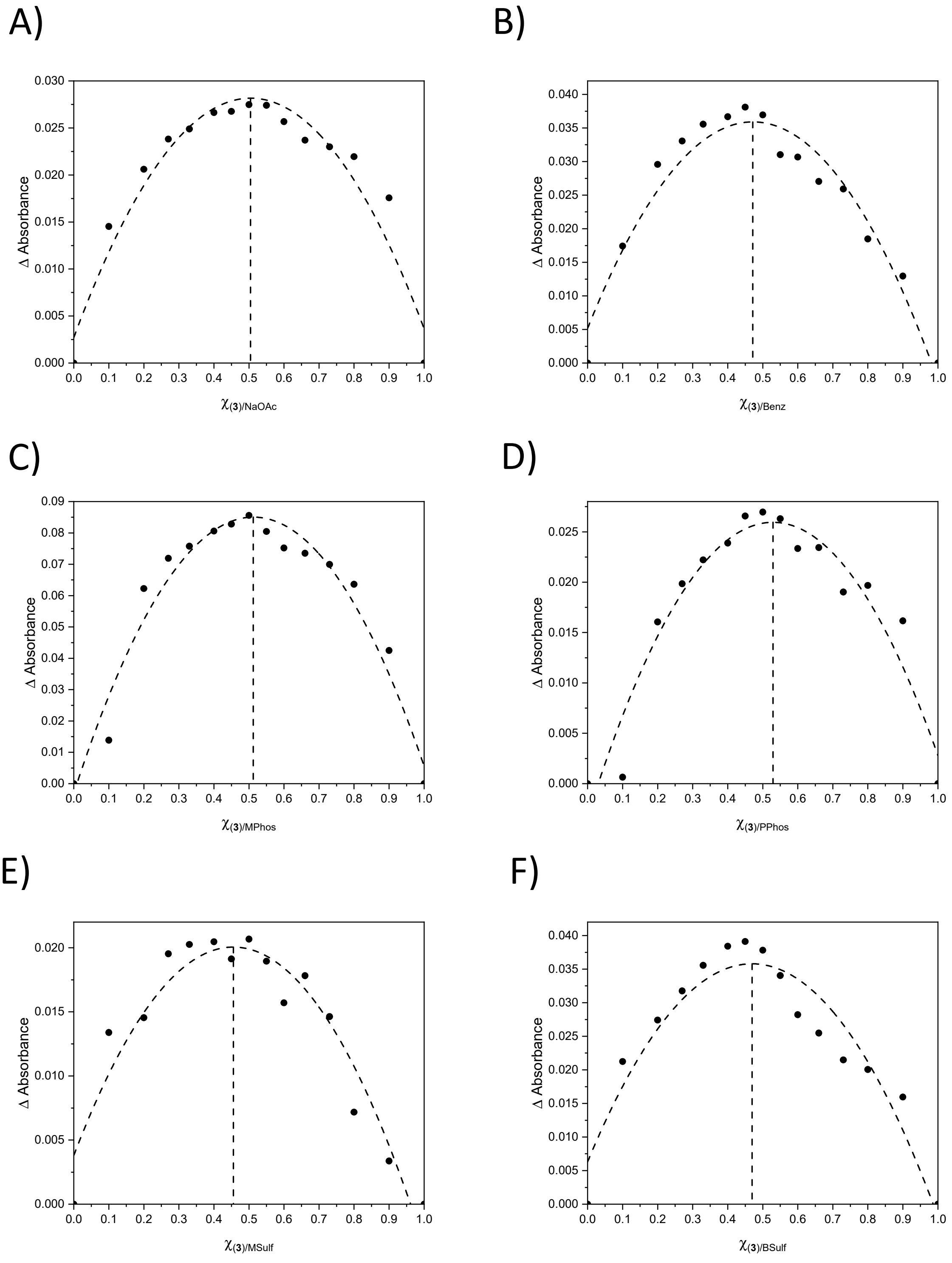


**Figure S43.** Job plots of the complex formation between receptor **3** and A) sodium acetate, B) benzoic acid, C) methylphosphonic acid, D) phenylphosphonic acid, E) methanesulfonic acid, F) benzenesulfonic acid, which confirm a 1:1 complex stoichiometry in all cases.

## 6. p$K_a$ determination

P$K_a$ values were determined by use of the Henderson-Hasselbach equation, using a pH-titration approach. Aqueous solutions of **5** and **6** (800 µL, 6.0 x $10^{-5}$ M) were prepared and adjusted to a high pH value (pH ≈ 12) with $NaOH_{aq}$. Absorption spectra (230-530 nm) were measured and $HCl_{aq}$ (0.1 mM) was subsequently added (0.5 – 5 µL) till the pH value reaches pH ≈ 3. After each addition of $HCl_{aq}$ the pH is measured and an absorption spectrum is recorded.

For each measured data point the ratio of [A⁻]/[HA] can be determined with the following formula:

$$\frac{[A^-]}{[HA]} = \frac{E_{lpH} - E}{E - E_{hpH}}$$

E: Extinction (pH dependent)
$E_{lpH}$: Extinction at lowest measured pH
$E_{hpH}$: Extinction at highest measured pH

For determination of the p$K_a$ value the Henderson-Hasselbach equation is used:

$$pH = pK_A + log\frac{[A^-]}{[HA]}$$

The pH-value is plotted against log([A⁻]/[HA]) for three different wavelengths (λ = 315 nm, 330 nm, 250 nm). The data points form a straight line, which intersects the y-axis at the p$K_a$ value. For a deeper understanding one may consult LD Chemistry Leaflets C4.2.2.1 [52].

### p$K_a$ determination of compound 2

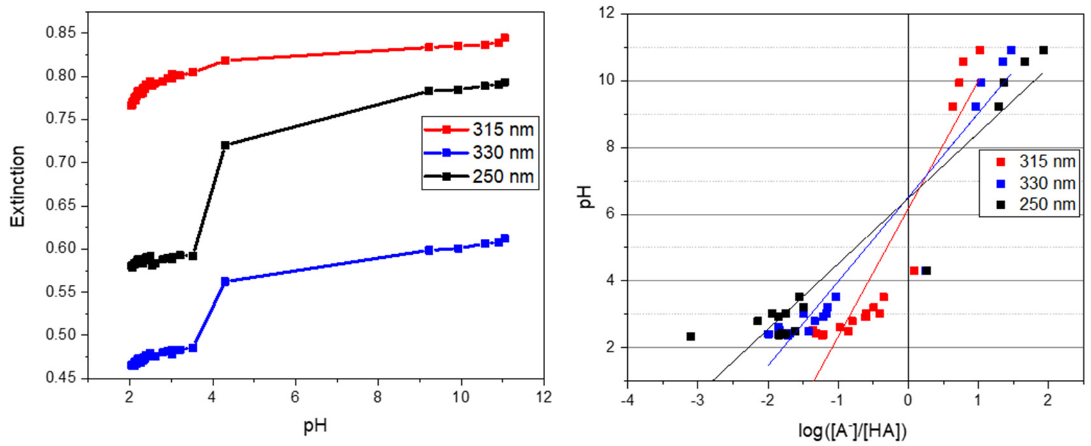


**Figure S44.** Plots of p$K_a$ measurement 1 of compound **2**.

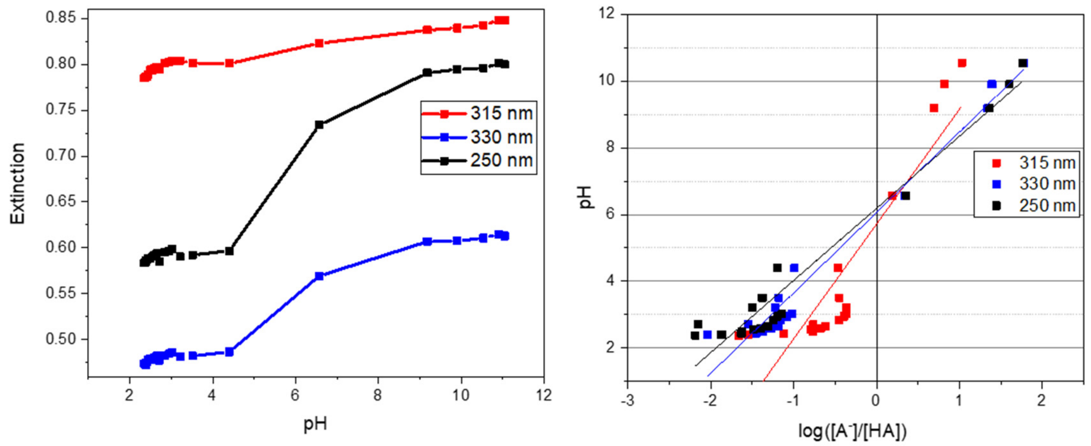


**Figure S45.** Plots of p$K_a$ measurement 2 of compound **2**.

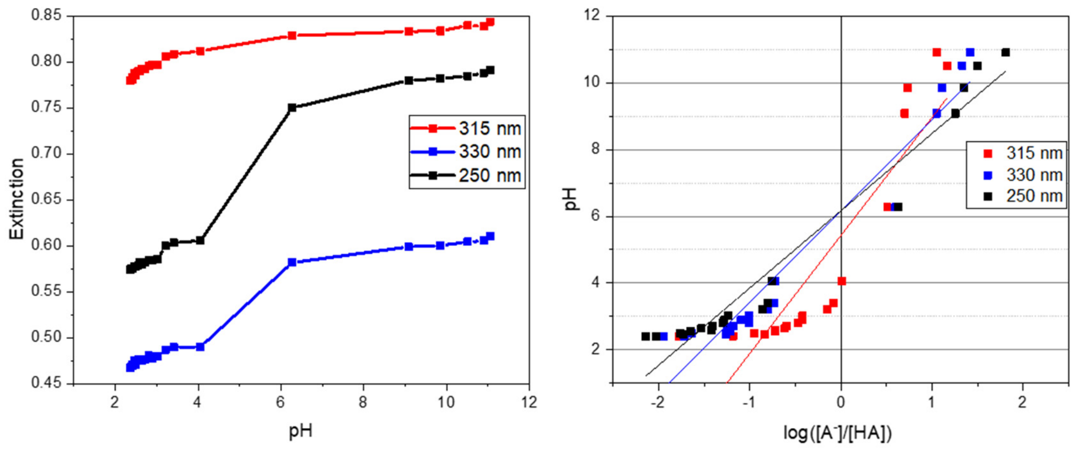


**Figure S46.** Plots of p$K_a$ measurement 3 of compound **2**.

**Table S4.** Calculated p$K_a$ values of **2.**

| | Wavelength [nm] | p$K_a$ |
|---|---|---|
| Measurement 1 | 315 | 6.18 |
| | 330 | 6.51 |
| | 250 | 6.48 |
| Measurement 2 | 315 | 5.72 |
| | 330 | 6.06 |
| | 250 | 6.19 |
| Measurement 3 | 315 | 6.17 |
| | 330 | 5.42 |
| | 250 | 6.17 |

**Substance 3**

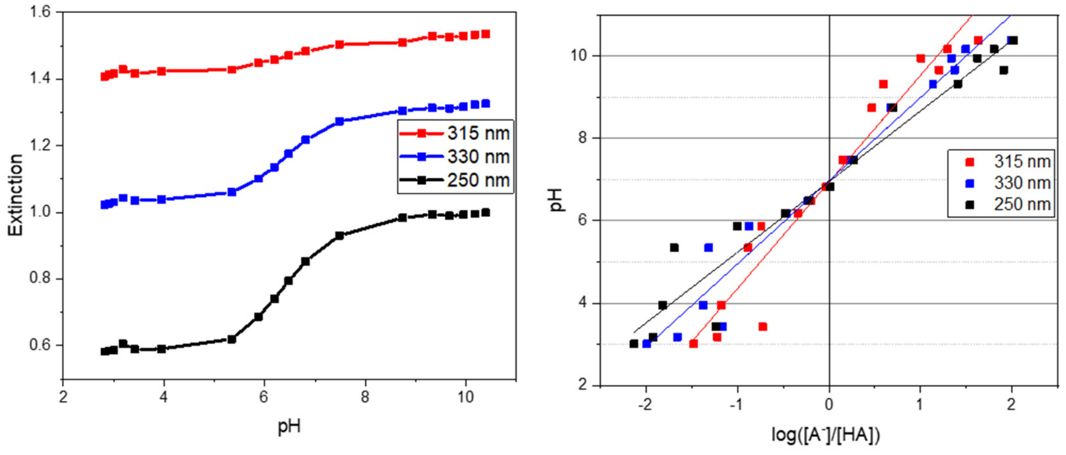


**Figure S47.** Plots of $pK_a$ measurement 1 of compound **3**.

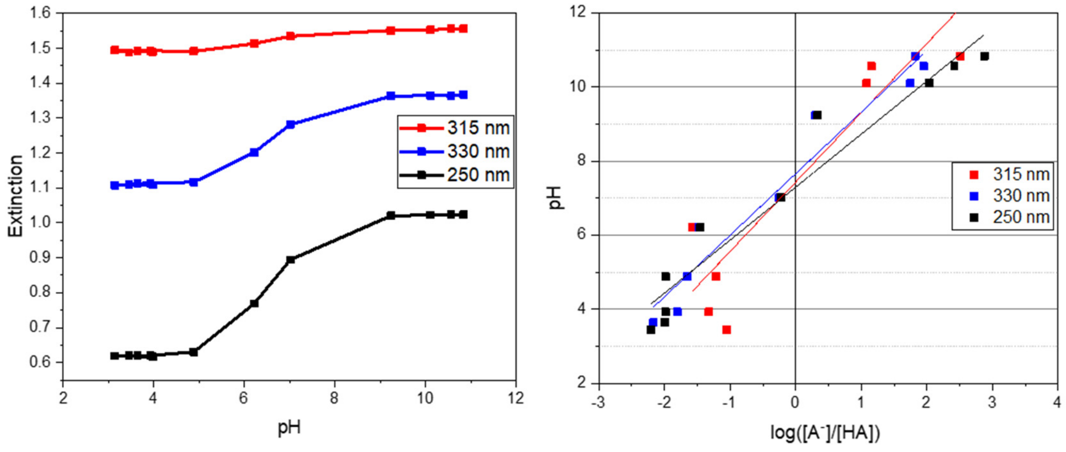


**Figure S48.** Plots of $pK_a$ measurement 2 of compound **3**.

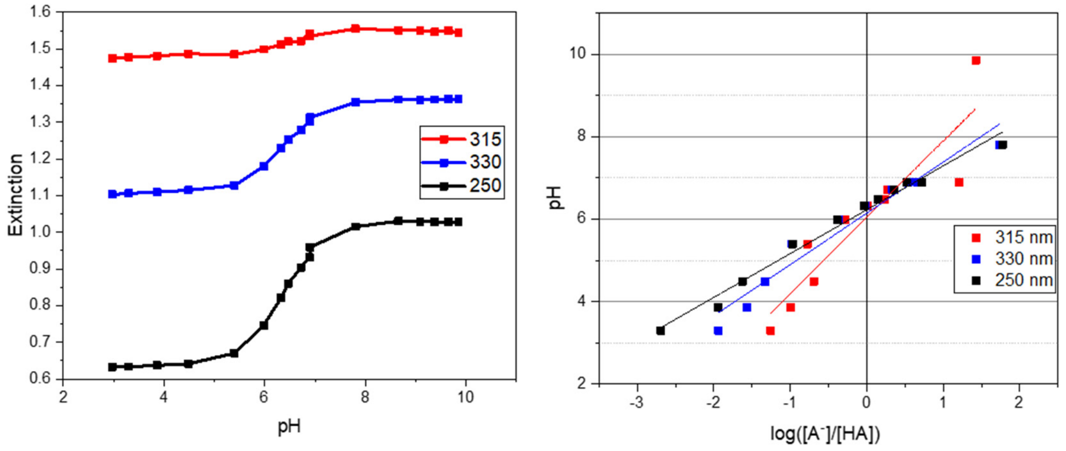


**Figure S49.** Plots of $pK_a$ measurement 3 of compound **3**.

**Table S5.** Calculated p$K_a$ values of **3.**

| | Wavelength [nm] | p$K_a$ |
|---|---|---|
| Measurement 1 | 315 | 6.94 |
| | 330 | 6.97 |
| | 250 | 6.95 |
| Measurement 2 | 315 | 7.43 |
| | 330 | 7.66 |
| | 250 | 7.30 |
| Measurement 3 | 315 | 6.04 |
| | 330 | 6.14 |
| | 250 | 6.23 |

**Table S6.** p$K_a$ values of **1, 2** and **3.**

| | p$K_a$ |
|---|---|
| **1** | 6.9 ± 0.2[(a)] |
| **2** | 6.1 ± 0.3 |
| **3** | 6.8 ± 0.5 |
| (a) value from literature [23]. | |

## 7. X-Ray crystallography

**Single-crystal X-ray analyses.**

The crystals were mounted on nylon loops in inert oil. Data were collected on a Bruker AXS D8 Venture diffractometer with Photon II detector (mono-chromated $Cu_{K\alpha}$ radiation, $\lambda$ = 1.54178 Å, micro-focus source) at 131(2) K. The structures were solved by Direct Methods (SHELXS-97)[53] and refined anisotropically by full-matrix least-squares on $F^2$ (SHELXL-2014)[54, 55]. Absorption corrections were performed semi-empirically from equivalent reflections on basis of multi-scans (Bruker AXS APEX3). Hydrogen atoms were refined using a riding model or rigid methyl groups. In crystal structure **1** NH and OH hydrogen atoms were refined freely. The bond lengths within the water molecules were restrained to be equal (SADI). (Refinable) Residual electron density can be found near any N or O atom. The maxima were chosen to match quantum chemical calculations of the hydrogen positions. They might be wrong and should be confirmed by other analytical means. The crystal diffracted rather poorly. No reflections beyond 0.84 Å of resolution could be obtained. Consequently, the model should be carefully interpreted and any conclusions draw from it supported by other means. Quantitative results may be unreliable. The absolute structure could *not* be determined reliably and was arbitrarily chosen. In crystal structure **2** NH and OH hydrogen atoms were refined freely. In the water molecules bond lengths and angle were restrained with DFIX and the isotropic displacement parameters were constrained to be equal to 1.2 times the equivalent *Uij* of the corresponding oxygen atom. In the final difference Fourier synthesis two larger residual density peaks remained. The location near hydrogen bond donor and acceptors made us assume they are a partially occupied water molecule disordered over two positions. However, the hydrogen atoms could not be identified but were added to the sum formula for completeness. Before anisotropic refinement the occupancies were constrained to the values of the free variables obtained during isotropic refinement. SIMU restraints were applied to the anisotropic displacement parameters.

CCDC-2150514 and 2150515 contain the supplementary crystallographic data for this paper. These data can be obtained free of charge from The Cambridge Crystallographic Data Centre via www.ccdc.cam.ac.uk/data_request/cif.

### 7.1. GCP-ethylamide (1)

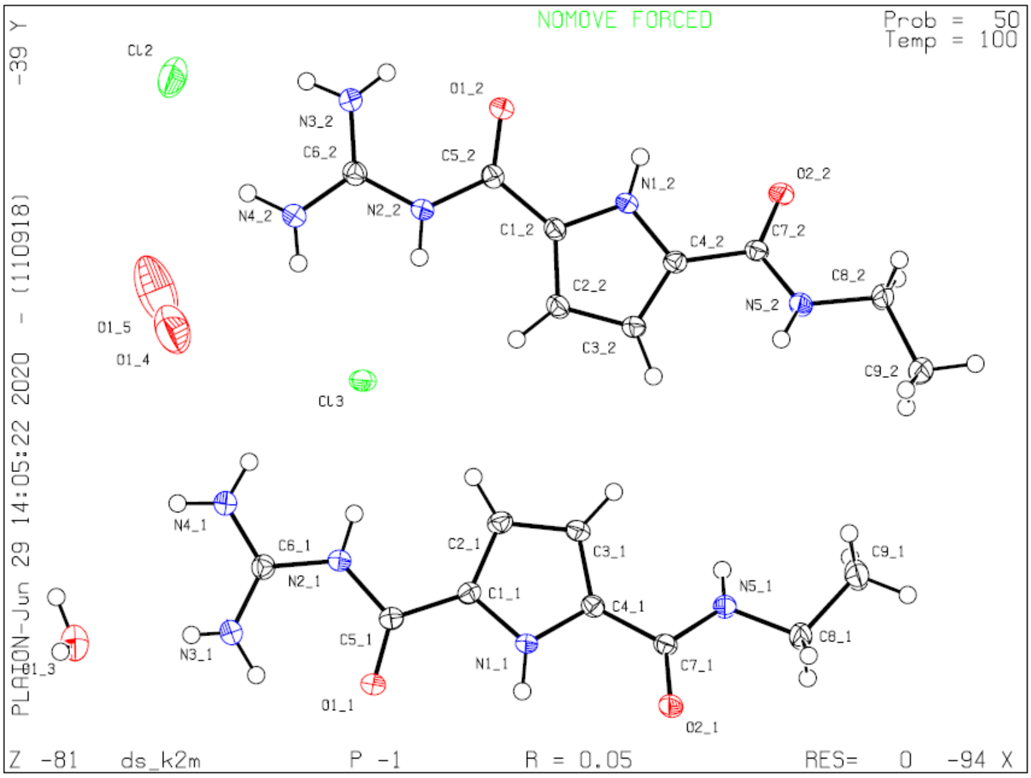


**Table S7.** Crystal data and structure refinement for **1**.

| **Identification code** | **1** |
|---|---|
| Empirical formula | $C_9\ H_{15.66}\ Cl\ N_5\ O_{2.83}$ |
| Formula weight | 274.65 |
| Density (calculated) | 1.464 g·cm$^{-3}$ |
| *F*(000) | 577 |
| Temperature | 100(2) K |
| Crystal size | 0.256 × 0.119 × 0.065 mm |
| Crystal colour | colourless |
| Crystal description | plate |
| Wavelength | 1.54178 Å |
| Crystal system | triclinic |
| Space group | $P\bar{1}$ |
| Unit cell dimensions | |
| *a* [Å] | 5.0421(4) |
| *b* [Å] | 15.7408(14) |
| *c* [Å] | 17.5287(15) |
| *α* [°] | 114.599(3) |
| *β* [°] | 94.776(3) |

| | |
|---|---|
| $\gamma$ [°] | 95.974(3) |
| Volume | 1245.68(18) Å$^3$ |
| $Z$ | 4 |
| Cell measurement reflections used | 9483 |
| Cell measurement $\vartheta$ min/max | 2.80°/80.09° |
| Diffractometer control software | Bruker APEX3(v2017.3-0) |
| Diffractometer measurement device | Bruker D8 Venture (Photon II detector) |
| Diffractometer measurement method | Data collection strategy APEX 3/Queen |
| $\vartheta$ range for data collection | 2.800°- 81.241° |
| Completeness to $\vartheta$ = 67.679° | 99.9% |
| Completeness to $\vartheta_{max}$ = 81.241° | 98.6% |
| Index ranges | $-6 \le h \le 6$ |
| | $-20 \le k \le 20$ |
| | $-22 \le l \le 22$ |
| Computing data reduction | Bruker APEX3(v2017.3-0) |
| Absorption coefficient | 2.821 mm$^{-1}$ |
| Absorption correction | Semi-empirical from equivalents |
| Computation absorption correction | SADABS |
| Max./min. Transmission | 0.75/0.58 |
| $R_{merg}$ before/after correction | 0.1301/0.0579 |
| Computing structure solution | Bruker APEX3(v2017.3-0) |
| Computing structure refinement | SHELXL-2017/1 (Sheldrick, 2017) |
| Refinement method | Full-matrix least-squares on $F^2$ |
| Reflections collected | 110495 |
| Independent reflections | 5437 |
| $R_{int}$ | 0.0378 |
| Reflections with $I > 2\sigma(I)$ | 5275 |
| Restraints | 9 |
| Parameter | 400 |
| GooF | 1.137 |
| Weighting details | $w = 1/[\sigma^2(F_{obs}^2) + (0.0440P)^2 + 3.0343P]$ |
| | where $P = (F_{obs}^2 + 2F_{calc}^2)/3$ |
| $R_1$ [$I > 2\sigma(I)$] | 0.0512 |
| $wR_2$ [$I > 2\sigma(I)$] | 0.1441 |
| $R_1$ [all data] | 0.0522 |
| $wR_2$ [all data] | 0.1446 |
| Largest diff. peak and hole | 0.727/-0.747 |

**Table S8.** Atomic coordinates ( × $10^4$) and equivalent isotropic displacement parameters (Å$^2$ × $10^3$) for **1**. $U_{eq}$ is defined as one third of the trace of the orthogonalized $U_{ij}$ tensor.

| | *x* | *y* | *z* | $U_{eq}$ |
|---|---|---|---|---|
| Cl(2) | 11513(2) | 1573(1) | 295(1) | 51(1) |
| Cl(3) | 4279(1) | 1472(1) | 3098(1) | 22(1) |
| O11 | -4256(4) | -179(1) | 3939(1) | 21(1) |
| O21 | -2643(3) | 2216(1) | 7246(1) | 22(1) |
| N11 | -1608(4) | 1201(2) | 5530(1) | 17(1) |
| N21 | -840(4) | 175(2) | 3279(1) | 19(1) |
| N31 | -4241(5) | -912(2) | 2246(2) | 23(1) |
| N41 | -564(5) | -246(2) | 1876(2) | 25(1) |
| N51 | 1846(4) | 2684(2) | 7642(1) | 22(1) |
| C11 | -572(5) | 991(2) | 4784(2) | 17(1) |
| C21 | 1919(5) | 1540(2) | 4962(2) | 20(1) |
| C31 | 2425(5) | 2082(2) | 5838(2) | 20(1) |
| C41 | 214(5) | 1862(2) | 6176(2) | 17(1) |
| C51 | -2096(5) | 284(2) | 3984(2) | 18(1) |
| C61 | -1968(5) | -341(2) | 2449(2) | 19(1) |
| C71 | -346(5) | 2262(2) | 7061(2) | 18(1) |
| C81 | 1645(5) | 3128(2) | 8545(2) | 28(1) |
| C91 | 4268(6) | 3748(2) | 9029(2) | 34(1) |
| O12 | 13382(4) | 4053(1) | 3980(1) | 22(1) |
| O22 | 14678(3) | 5214(1) | 7299(1) | 21(1) |
| N12 | 12081(4) | 4374(2) | 5591(1) | 17(1) |
| N22 | 9512(4) | 2984(2) | 3341(1) | 19(1) |
| N32 | 12089(5) | 3000(2) | 2293(2) | 22(1) |
| N42 | 8265(5) | 1915(2) | 1949(2) | 24(1) |
| N52 | 10586(4) | 5232(2) | 7739(1) | 21(1) |
| C12 | 10427(5) | 3783(2) | 4855(2) | 18(1) |
| C22 | 8098(5) | 3437(2) | 5066(2) | 20(1) |
| C32 | 8355(5) | 3843(2) | 5954(2) | 20(1) |
| C42 | 10845(5) | 4415(2) | 6262(2) | 17(1) |
| C52 | 11286(5) | 3634(2) | 4041(2) | 18(1) |
| C62 | 10018(5) | 2627(2) | 2514(2) | 19(1) |
| C72 | 12195(5) | 4986(2) | 7140(2) | 17(1) |
| C82 | 11696(5) | 5842(2) | 8625(2) | 25(1) |
| C92 | 9701(6) | 5827(2) | 9213(2) | 30(1) |
| O13 | -6365(6) | -2010(2) | 537(2) | 49(1) |
| O14 | 4110(30) | 241(9) | 887(8) | 64(3) |

| | | | | |
|---|---|---|---|---|
| O15 | 5340(40) | 229(14) | 621(13) | 87(6) |

**Table S9.** Anisotropic displacement parameters (Å$^2$ × 10$^3$) for **1**.

| | $U_{11}$ | $U_{22}$ | $U_{33}$ | $U_{23}$ | $U_{13}$ | $U_{12}$ |
|---|---|---|---|---|---|---|
| Cl(2) | 51(1) | 65(1) | 25(1) | 6(1) | 11(1) | 7(1) |
| Cl(3) | 18(1) | 24(1) | 24(1) | 12(1) | 4(1) | -1(1) |
| O11 | 19(1) | 22(1) | 21(1) | 9(1) | 3(1) | -3(1) |
| O21 | 13(1) | 27(1) | 20(1) | 5(1) | 3(1) | 0(1) |
| N11 | 13(1) | 18(1) | 16(1) | 7(1) | 3(1) | -1(1) |
| N21 | 16(1) | 21(1) | 18(1) | 7(1) | 3(1) | -1(1) |
| N31 | 22(1) | 24(1) | 18(1) | 6(1) | 1(1) | -2(1) |
| N41 | 25(1) | 28(1) | 17(1) | 7(1) | 2(1) | -5(1) |
| N51 | 12(1) | 30(1) | 18(1) | 5(1) | 4(1) | 1(1) |
| C11 | 17(1) | 18(1) | 17(1) | 8(1) | 4(1) | 3(1) |
| C21 | 16(1) | 24(1) | 20(1) | 11(1) | 5(1) | 4(1) |
| C31 | 15(1) | 23(1) | 21(1) | 10(1) | 1(1) | -2(1) |
| C41 | 14(1) | 18(1) | 19(1) | 7(1) | 1(1) | 3(1) |
| C51 | 17(1) | 18(1) | 19(1) | 9(1) | 3(1) | 3(1) |
| C61 | 21(1) | 18(1) | 18(1) | 7(1) | 3(1) | 3(1) |
| C71 | 15(1) | 17(1) | 19(1) | 7(1) | 2(1) | 1(1) |
| C81 | 18(1) | 38(2) | 18(1) | 3(1) | 4(1) | -1(1) |
| C91 | 22(1) | 43(2) | 22(1) | 1(1) | 2(1) | -2(1) |
| O12 | 20(1) | 24(1) | 20(1) | 9(1) | 2(1) | -4(1) |
| O22 | 13(1) | 29(1) | 21(1) | 10(1) | 2(1) | 0(1) |
| N12 | 13(1) | 20(1) | 17(1) | 7(1) | 1(1) | -2(1) |
| N22 | 16(1) | 21(1) | 18(1) | 7(1) | 3(1) | -2(1) |
| N32 | 22(1) | 23(1) | 17(1) | 7(1) | 3(1) | -3(1) |
| N42 | 22(1) | 26(1) | 19(1) | 7(1) | 2(1) | -5(1) |
| N52 | 11(1) | 29(1) | 18(1) | 6(1) | 1(1) | 1(1) |
| C12 | 16(1) | 18(1) | 17(1) | 7(1) | -1(1) | 0(1) |
| C22 | 16(1) | 21(1) | 19(1) | 6(1) | 1(1) | 1(1) |
| C32 | 16(1) | 21(1) | 19(1) | 6(1) | 4(1) | 0(1) |
| C42 | 15(1) | 18(1) | 19(1) | 7(1) | 4(1) | 4(1) |
| C52 | 17(1) | 18(1) | 18(1) | 7(1) | 1(1) | 2(1) |
| C62 | 18(1) | 20(1) | 20(1) | 10(1) | 1(1) | 2(1) |
| C72 | 14(1) | 18(1) | 19(1) | 9(1) | 2(1) | 1(1) |
| C82 | 17(1) | 34(1) | 16(1) | 6(1) | 1(1) | 0(1) |
| C92 | 22(1) | 41(2) | 20(1) | 7(1) | 4(1) | -2(1) |
| O13 | 46(2) | 57(2) | 26(1) | 6(1) | 2(1) | -8(1) |

| | | | | | | |
|---|---|---|---|---|---|---|
| O14 | 85(9) | 43(5) | 50(6) | 14(4) | -13(5) | -8(6) |
| O15 | 89(13) | 60(8) | 84(13) | 9(9) | -41(9) | 24(10) |

**Table S10.** Bond lengths [Å] for **1**.

| | |
|---|---|
| O11-C51 | 1.222(3) |
| O21-C71 | 1.231(3) |
| N11-C41 | 1.362(3) |
| N11-C11 | 1.373(3) |
| N21-C61 | 1.372(3) |
| N21-C51 | 1.392(3) |
| N31-C61 | 1.303(3) |
| N41-C61 | 1.323(3) |
| N51-C71 | 1.341(3) |
| N51-C81 | 1.456(3) |
| C11-C21 | 1.381(3) |
| C11-C51 | 1.461(3) |
| C21-C31 | 1.396(4) |
| C31-C41 | 1.384(3) |
| C41-C71 | 1.476(3) |
| C81-C91 | 1.509(4) |
| O12-C52 | 1.223(3) |
| O22-C72 | 1.241(3) |
| N12-C42 | 1.358(3) |
| N12-C12 | 1.374(3) |
| N22-C62 | 1.377(3) |
| N22-C52 | 1.391(3) |
| N32-C62 | 1.309(3) |
| N42-C62 | 1.320(3) |
| N52-C72 | 1.333(3) |
| N52-C82 | 1.466(3) |
| C12-C22 | 1.386(3) |
| C12-C52 | 1.457(3) |
| C22-C32 | 1.402(4) |
| C32-C42 | 1.388(3) |
| C42-C72 | 1.479(3) |
| C82-C92 | 1.505(4) |

**Table S11.** Bond angles [°] for **1**.

| | |
|---|---|
| C41-N11-C11 | 108.6(2) |
| C61-N21-C51 | 125.9(2) |
| C71-N51-C81 | 121.5(2) |
| N11-C11-C21 | 108.3(2) |
| N11-C11-C51 | 120.3(2) |
| C21-C11-C51 | 131.5(2) |
| C11-C21-C31 | 107.3(2) |
| C41-C31-C21 | 107.4(2) |
| N11-C41-C31 | 108.4(2) |
| N11-C41-C71 | 121.8(2) |
| C31-C41-C71 | 129.7(2) |
| O11-C51-N21 | 123.0(2) |
| O11-C51-C11 | 123.3(2) |
| N21-C51-C11 | 113.7(2) |
| N31-C61-N41 | 122.7(3) |
| N31-C61-N21 | 121.3(2) |
| N41-C61-N21 | 115.9(2) |
| O21-C71-N51 | 123.0(2) |
| O21-C71-C41 | 122.5(2) |
| N51-C71-C41 | 114.5(2) |
| N51-C81-C91 | 109.9(2) |
| C42-N12-C12 | 109.0(2) |
| C62-N22-C52 | 125.6(2) |
| C72-N52-C82 | 120.5(2) |
| N12-C12-C22 | 108.3(2) |
| N12-C12-C52 | 119.6(2) |
| C22-C12-C52 | 132.1(2) |
| C12-C22-C32 | 106.9(2) |
| C42-C32-C22 | 107.6(2) |
| N12-C42-C32 | 108.2(2) |
| N12-C42-C72 | 120.9(2) |
| C32-C42-C72 | 130.9(2) |
| O12-C52-N22 | 122.9(2) |
| O12-C52-C12 | 122.7(2) |
| N22-C52-C12 | 114.4(2) |
| N32-C62-N42 | 121.5(2) |
| N32-C62-N22 | 121.5(2) |
| N42-C62-N22 | 117.0(2) |
| O22-C72-N52 | 122.8(2) |

| | |
|---|---|
| O22-C72-C42 | 121.2(2) |
| N52-C72-C42 | 116.0(2) |
| N52-C82-C92 | 111.3(2) |

### 7.2. GCl-ethylamide (2)

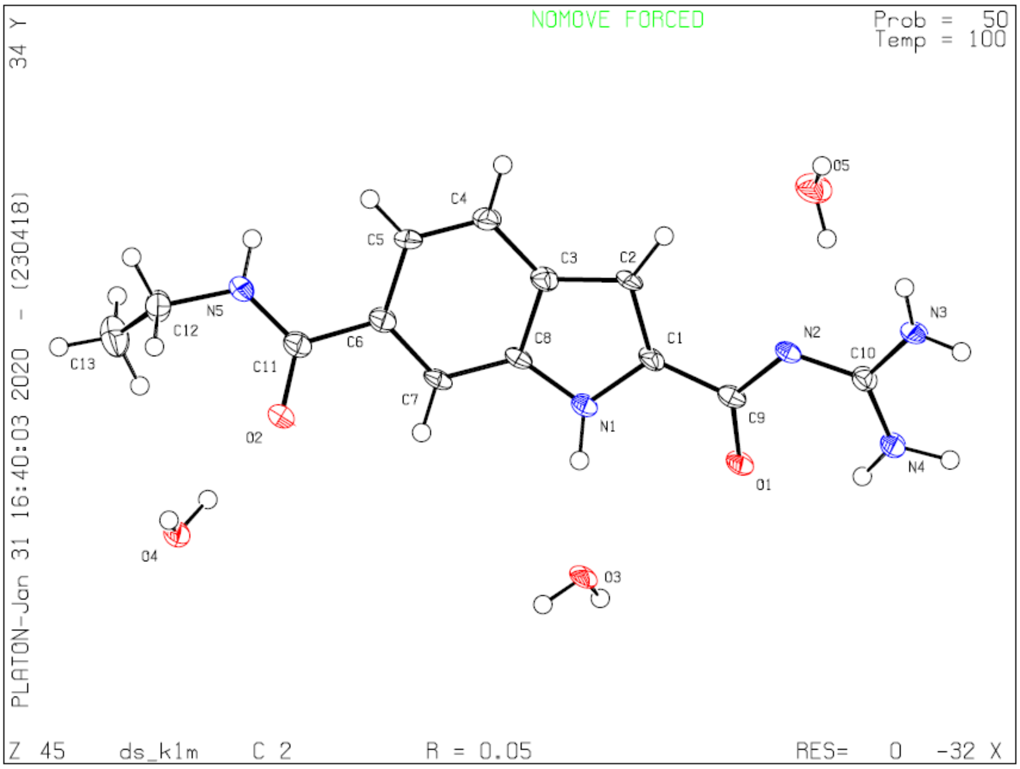


**Table S12.** Crystal data and structure refinement for **2**.

| **Identification code** | **K** |
|---|---|
| Empirical formula | C13 H19 N5 O4 |
| Formula weight | 309.33 |
| Density (calculated) | 1.389 g·cm$^{-3}$ |
| *F*(000) | 656 |
| Temperature | 100(2) K |
| Crystal size | 0.048 × 0.032 × 0.032 mm |
| Crystal colour | colourless |
| Crystal description | needle |
| Wavelength | 1.54178 Å |
| Crystal system | monoclinic |
| Space group | *C*2 |
| Unit cell dimensions | |
| *a* [Å] | 20.149(2) |
| *b* [Å] | 6.3428(7) |
| *c* [Å] | 13.6572(14) |
| *α* [°] | 90 |
| *β* [°] | 122.048(6) |
| *γ* [°] | 90 |

| | |
|---|---|
| Volume | 1479.4(3) Å$^3$ |
| *Z* | 4 |
| Cell measurement reflections used | 6448 |
| Cell measurement $\vartheta$ min/max | 3.82°/68.55° |
| Diffractometer control software | Bruker APEX3(v2017.3-0) |
| Diffractometer measurement device | Bruker D8 Venture (Photon II detector) |
| Diffractometer measurement method | Data collection strategy APEX 3/Queen |
| $\vartheta$ range for data collection | 3.818°- 68.548° |
| Completeness to $\vartheta$ = 67.679° | 99.6% |
| Completeness to $\vartheta_{max}$ = 68.548° | 99.4% |
| Index ranges | $-24 \le h \le 24$<br>$-7 \le k \le 7$<br>$-16 \le l \le 16$ |
| Computing data reduction | Bruker APEX3 (v2017.3-0) |
| Absorption coefficient | 0.882 mm$^{-1}$ |
| Absorption correction | Semi-empirical from equivalents |
| Computation absorption correction | SADABS |
| Max./min. Transmission | 0.75/0.50 |
| $R_{merg}$ before/after correction | 0.1550/0.0777 |
| Computing structure solution | Bruker APEX3(v2017.3-0) |
| Computing structure refinement | SHELXL-2017/1 (Sheldrick, 2017) |
| Refinement method | Full-matrix least-squares on $F^2$ |
| Reflections collected | 11305 |
| Independent reflections | 2636 |
| $R_{int}$ | 0.0759 |
| Reflections with $I > 2\sigma(I)$ | 2301 |
| Restraints | 7 |
| Parameter | 241 |
| GooF | 0.955 |
| Weighting details | $w = 1/[\sigma^2(F_{obs}^2) + (0.1000P)^2]$<br>where $P = (F_{obs}^2 + 2F_{calc}^2)/3$ |
| $R_1$ [$I > 2\sigma(I)$] | 0.0468 |
| $wR_2$ [$I > 2\sigma(I)$] | 0.1279 |
| $R_1$ [all data] | 0.0559 |
| $wR_2$ [all data] | 0.1375 |
| Absolute structure parameter | 0.4(3) |
| Largest diff. peak and hole | 0.242/-0.224 |

**Table S13.** Atomic coordinates ( × 10$^4$) and equivalent isotropic displacement parameters (Å$^2$ × 10$^3$) for **2**. $U_{eq}$ is defined as one third of the trace of the orthogonalized $U_{ij}$ tensor.

| | *x* | *y* | *z* | $U_{eq}$ |
|---|---|---|---|---|
| O(1) | 9229(2) | 8534(5) | 7876(2) | 22(1) |
| O(2) | 9129(2) | 5711(5) | 13384(3) | 27(1) |
| O(3) | 10000 | 5003(7) | 10000 | 20(1) |
| O(4) | 10000 | 2670(7) | 15000 | 23(1) |
| O(5) | 8042(2) | 15341(6) | 7120(3) | 33(1) |

| | | | | |
|---|---|---|---|---|
| N(1) | 8965(2) | 8451(6) | 9700(3) | 20(1) |
| N(2) | 9290(2) | 12205(6) | 8009(3) | 19(1) |
| N(3) | 9722(2) | 14473(6) | 7172(3) | 21(1) |
| N(4) | 9653(2) | 10983(6) | 6701(3) | 23(1) |
| N(5) | 8297(2) | 7828(6) | 13556(3) | 22(1) |
| C(1) | 8882(2) | 10242(7) | 9074(3) | 19(1) |
| C(2) | 8556(2) | 11828(7) | 9380(4) | 19(1) |
| C(3) | 8464(2) | 10994(7) | 10271(3) | 19(1) |
| C(4) | 8199(2) | 11831(7) | 10962(4) | 22(1) |
| C(5) | 8248(2) | 10632(7) | 11825(4) | 22(1) |
| C(6) | 8540(2) | 8517(7) | 12027(4) | 21(1) |
| C(7) | 8769(2) | 7636(7) | 11323(4) | 19(1) |
| C(8) | 8735(2) | 8897(6) | 10457(3) | 18(1) |
| C(9) | 9150(2) | 10247(7) | 8248(3) | 19(1) |
| C(10) | 9555(2) | 12487(7) | 7290(3) | 19(1) |
| C(11) | 8666(2) | 7252(7) | 13037(4) | 22(1) |
| C(12) | 8422(3) | 6781(8) | 14587(4) | 26(1) |
| C(13) | 7923(3) | 4835(9) | 14318(4) | 38(1) |

**Table S14.** Anisotropic displacement parameters (Å$^2$ × 10$^3$) for **2**.

| | $U_{11}$ | $U_{22}$ | $U_{33}$ | $U_{23}$ | $U_{13}$ | $U_{12}$ |
|---|---|---|---|---|---|---|
| O(1) | 34(2) | 7(2) | 29(2) | -1(1) | 19(1) | -2(1) |
| O(2) | 36(2) | 20(2) | 31(2) | 10(1) | 23(1) | 11(1) |
| O(3) | 29(2) | 7(2) | 25(2) | 0 | 14(2) | 0 |
| O(4) | 34(2) | 14(2) | 26(2) | 0 | 19(2) | 0 |
| O(5) | 34(2) | 19(2) | 48(2) | 0(2) | 24(2) | -2(2) |
| N(1) | 28(2) | 10(2) | 25(2) | 1(1) | 16(2) | 0(2) |
| N(2) | 26(2) | 9(2) | 24(2) | 1(1) | 15(2) | 1(1) |
| N(3) | 29(2) | 9(2) | 30(2) | -1(1) | 19(2) | 0(2) |
| N(4) | 37(2) | 10(2) | 31(2) | 0(2) | 24(2) | -2(2) |
| N(5) | 29(2) | 16(2) | 29(2) | 6(2) | 19(2) | 3(2) |
| C(1) | 26(2) | 8(2) | 23(2) | 1(2) | 13(2) | -2(2) |
| C(2) | 25(2) | 7(2) | 26(2) | 3(2) | 14(2) | 3(2) |
| C(3) | 22(2) | 12(2) | 24(2) | -2(2) | 12(2) | -2(2) |

| | | | | | | |
|---|---|---|---|---|---|---|
| C(4) | 26(2) | 14(2) | 31(2) | 1(2) | 18(2) | 2(2) |
| C(5) | 28(2) | 13(2) | 29(2) | 2(2) | 18(2) | 6(2) |
| C(6) | 26(2) | 13(2) | 24(2) | -1(2) | 14(2) | -2(2) |
| C(7) | 23(2) | 7(2) | 26(2) | 1(2) | 12(2) | 1(2) |
| C(8) | 20(2) | 8(2) | 21(2) | 1(2) | 9(2) | 0(2) |
| C(9) | 22(2) | 10(2) | 23(2) | -1(2) | 10(2) | -1(2) |
| C(10) | 21(2) | 15(2) | 21(2) | 1(2) | 11(2) | -1(2) |
| C(11) | 27(2) | 15(2) | 26(2) | 0(2) | 14(2) | -1(2) |
| C(12) | 32(2) | 27(3) | 25(2) | 2(2) | 18(2) | -1(2) |
| C(13) | 47(3) | 34(3) | 35(3) | 5(2) | 24(2) | -8(2) |

**Table S15.** Bond lengths [Å] for **2**.

| | |
|---|---|
| O(1)-C(9) | 1.245(5) |
| O(2)-C(11) | 1.257(5) |
| N(1)-C(8) | 1.369(5) |
| N(1)-C(1) | 1.377(5) |
| N(2)-C(9) | 1.351(6) |
| N(2)-C(10) | 1.357(5) |
| N(3)-C(10) | 1.335(6) |
| N(4)-C(10) | 1.327(6) |
| N(5)-C(11) | 1.321(6) |
| N(5)-C(12) | 1.453(6) |
| C(1)-C(2) | 1.382(6) |
| C(1)-C(9) | 1.487(6) |
| C(2)-C(3) | 1.425(6) |
| C(3)-C(8) | 1.409(6) |
| C(3)-C(4) | 1.411(6) |
| C(4)-C(5) | 1.361(6) |
| C(5)-C(6) | 1.432(7) |
| C(6)-C(7) | 1.385(6) |
| C(6)-C(11) | 1.498(6) |
| C(7)-C(8) | 1.399(6) |
| C(12)-C(13) | 1.510(7) |

**Table S16**: Bond angles [°] for **2**.

| | |
|---|---|
| C(8)-N(1)-C(1) | 108.6(4) |
| C(9)-N(2)-C(10) | 120.6(4) |
| C(11)-N(5)-C(12) | 122.4(4) |
| N(1)-C(1)-C(2) | 109.5(3) |
| N(1)-C(1)-C(9) | 119.9(4) |
| C(2)-C(1)-C(9) | 130.6(4) |
| C(1)-C(2)-C(3) | 106.6(4) |
| C(8)-C(3)-C(4) | 118.8(4) |
| C(8)-C(3)-C(2) | 106.9(4) |
| C(4)-C(3)-C(2) | 134.2(4) |
| C(5)-C(4)-C(3) | 119.3(4) |
| C(4)-C(5)-C(6) | 121.6(4) |
| C(7)-C(6)-C(5) | 119.9(4) |
| C(7)-C(6)-C(11) | 117.9(4) |
| C(5)-C(6)-C(11) | 122.1(4) |
| C(6)-C(7)-C(8) | 118.1(4) |
| N(1)-C(8)-C(7) | 129.5(4) |
| N(1)-C(8)-C(3) | 108.2(4) |
| C(7)-C(8)-C(3) | 122.2(4) |
| O(1)-C(9)-N(2) | 127.9(4) |
| O(1)-C(9)-C(1) | 119.0(4) |
| N(2)-C(9)-C(1) | 113.1(4) |
| N(4)-C(10)-N(3) | 118.5(4) |
| N(4)-C(10)-N(2) | 125.8(4) |
| N(3)-C(10)-N(2) | 115.6(4) |
| O(2)-C(11)-N(5) | 122.0(4) |
| O(2)-C(11)-C(6) | 119.5(4) |
| N(5)-C(11)-C(6) | 118.5(4) |
| N(5)-C(12)-C(13) | 112.8(4) |

## 8. Lowest energy conformations

Lowest energy conformations were calculated using Maestro© 12.4 by Schrödinger LLC. Hereby the structures of (**1**), (**2**) and (**3**) were paired with acetate, benzoic acid, methylphosphonic acid, phenylphosphonic acid, methanesulfonic acid and benzenesulfonic acid respectively. In each calculation the structures were prepared by LigPrep to generate tautomers and possible states at pH = 7±2. The receptor-ligand pair was pre-coordinated by minimization and calculation of the potential energy performed using MacroModel conformational search. The force field used was OPLS 2005. Conformational search with mixed torsional/low-mode sampling and water as solvent was used. The conformation with the lowest potential energy was considered.

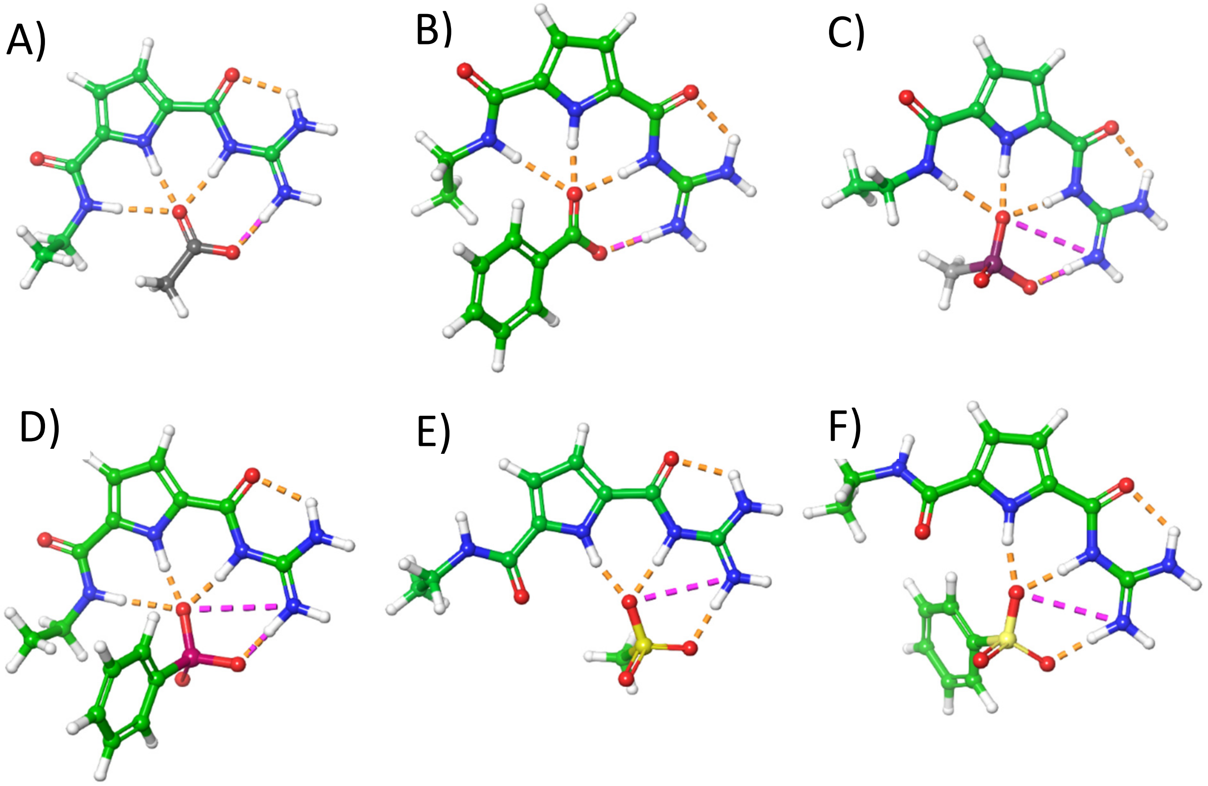


**Figure S50.** Calculated complex conformations with the lowest potential energy of **1** with A) sodium acetate, B) benzoic acid, C) methylphosphonic acid, D) phenylphosphonic acid, E) methanesulfonic acid and F) benzenesulfonic acid. (Ionic interactions are depicted in pink, H-bonds in orange).

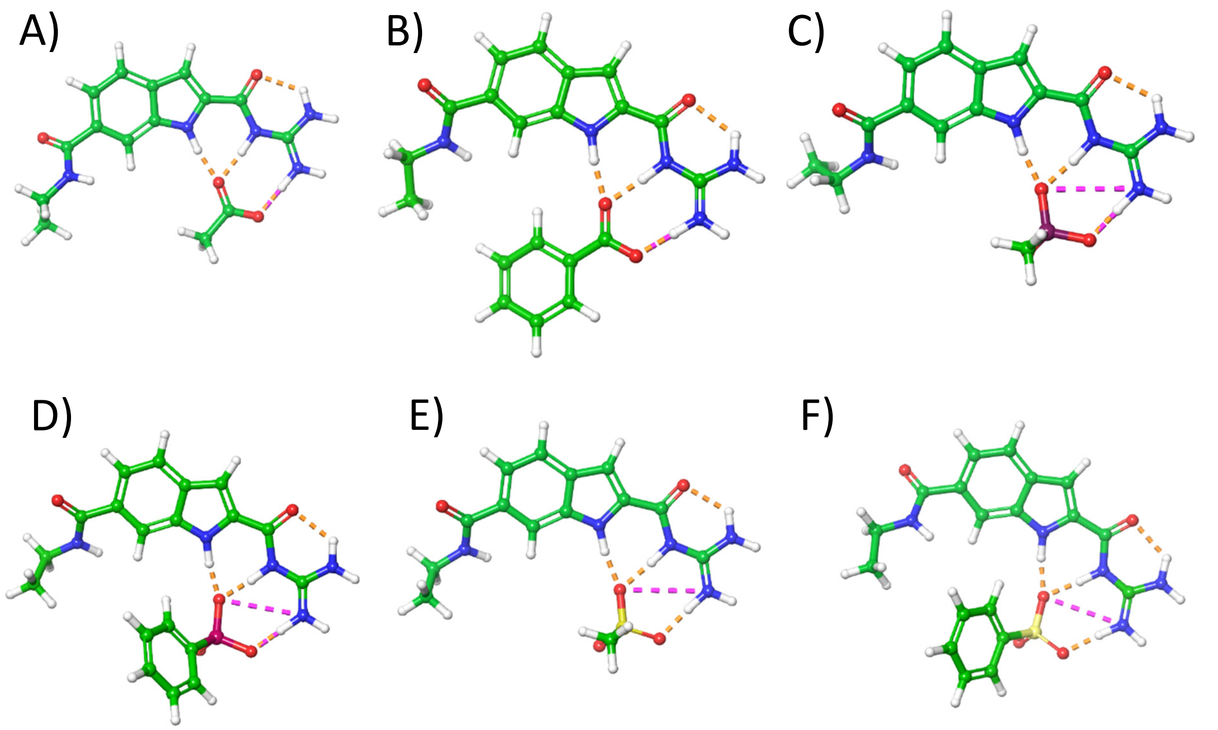


**Figure S51.** Calculated complex conformations with the lowest potential energy of **2** with A) sodium acetate, B) benzoic acid, C) methylphosphonic acid, D) phenylphosphonic acid, E) methanesulfonic acid and F) benzenesulfonic acid. (Ionic interactions are depicted in pink, H-bonds in orange).

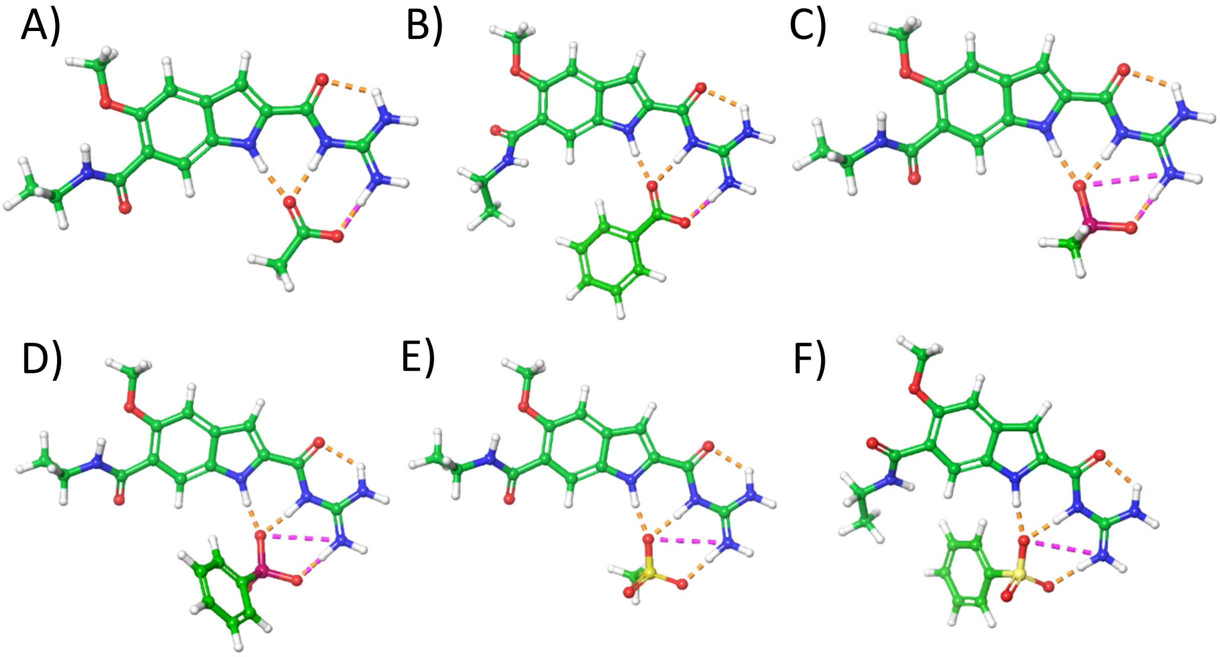


**Figure S52.** Calculated complex conformations with the lowest potential energy of **3** with A) sodium acetate, B) benzoic acid, C) methylphosphonic acid, D) phenylphosphonic acid, E) methanesulfonic acid and F) benzenesulfonic acid. (Ionic interactions are depicted in pink, H-bonds in orange).

## 9. DFT Calculations

The theoretical calculations were conducted using Gaussian 09’ as software [56] and a high performance computing cluster of the University Duisburg-Essen. The calculations, which have been carried out involved the density functional theory (DFT) with Becke’s three-parameter hybrid exchange functional and the Lee-Yang-Parr correlation functional (B3LYP) and 6-311G (++) (d,p) basis set.[57–59] All structures were optimized in the gas phase, and the nature of the stationary point (in these case minima on the potential energy surface) was confirmed by the normal-mode analysis. Molecular orbital contributions were determined using Gauss Sum 2.2. as software.[60]

For DFT not only the chloride-salts (**1**, **2** and **3**) were considered, but also the unprotonated molecules (**1a**, **2a**, **3a**) as well as variations with different electronic structure (**1b**, **2b**, **3b**), as these structures are dependent on the pH-value of the examined media and therefore of interest in this investigation.

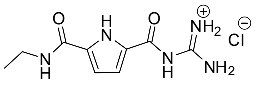

**(1)**

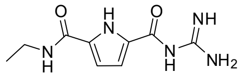

**(1a)**

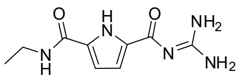

**(1b)**

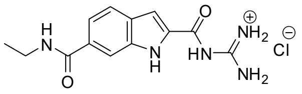

**(2)**

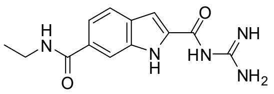

**(2a)**

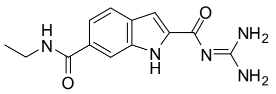

**(2b)**

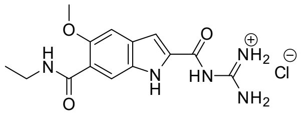

**(3)**

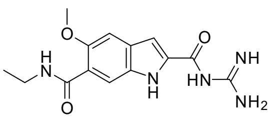

**(3a)**

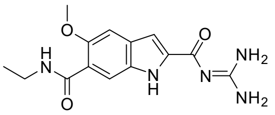

**(3b)**

**Figure S53.** Considered molecular structures for DFT calculations.

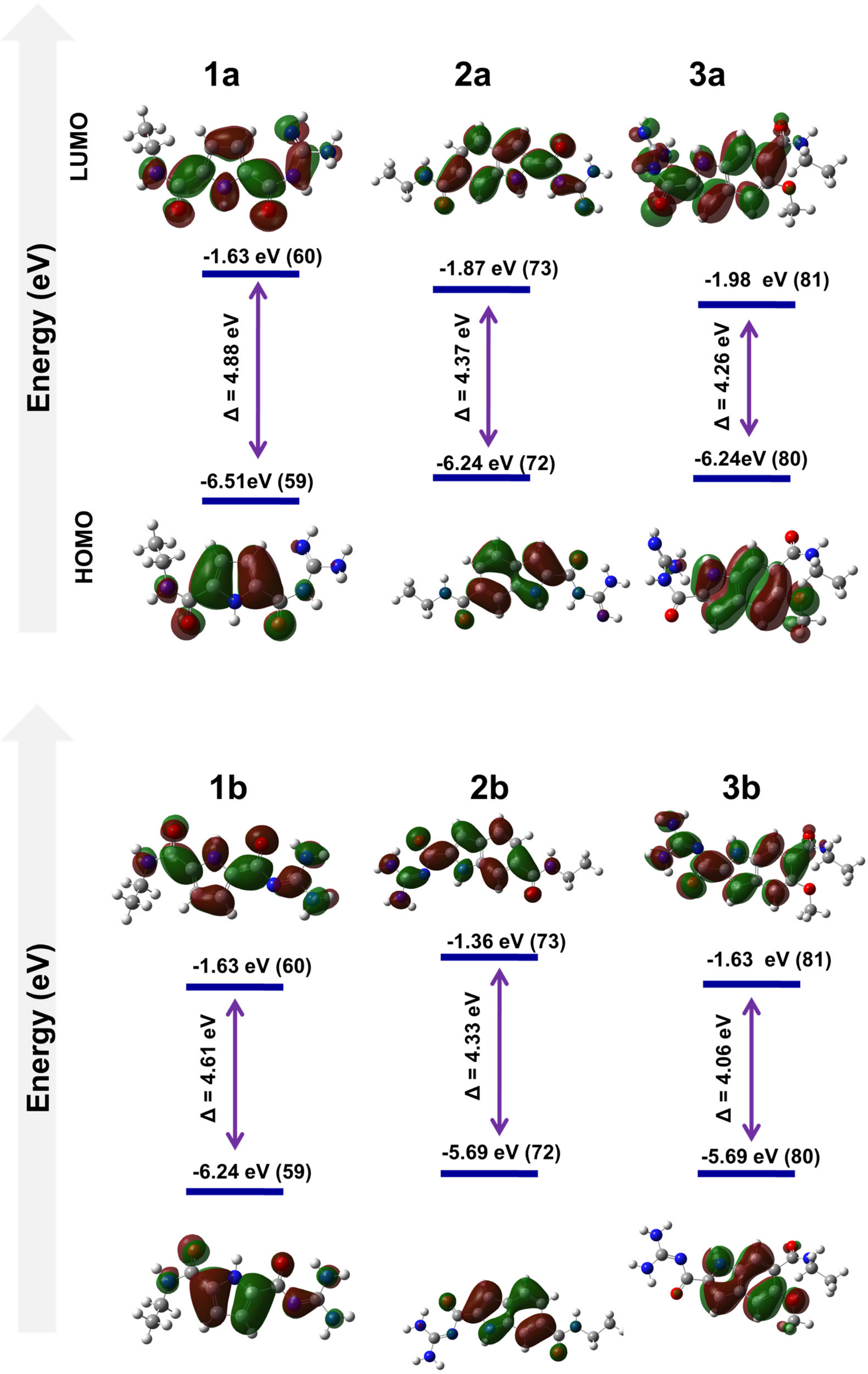


**Figure S54.** HOMO and LUMO energies for structures **1a**, **2a**, **3a**, **1b**, **2b** and **3b**.

## List of Calculated Coordinates for the optimized geometry

### Coordinates of 1:

### Charge = 1 Multiplicity = 1

| | | | |
|---|---|---|---|
| C | -2.9499 | 0.1382 | 0.5021 |
| C | -1.5991 | -0.2255 | 0.7179 |
| C | -0.8207 | 0.6155 | -0.0458 |
| N | -1.6545 | 1.4734 | -0.7145 |
| C | -2.9548 | 1.19 | -0.3867 |
| C | 0.528 | 0.5979 | -0.1224 |
| C | -4.0491 | 1.8301 | -0.8536 |
| O | 1.1031 | 1.3897 | -0.8307 |
| O | -4.1395 | 3.0298 | -0.7447 |
| N | 1.243 | -0.3171 | 0.6027 |
| N | -5.0555 | 1.1192 | -1.4501 |
| C | 0.5527 | -1.2675 | 1.4528 |
| C | -0.2553 | -0.5167 | 2.5031 |
| C | -4.9549 | -0.2166 | -1.5713 |
| N | -5.8856 | -0.874 | -2.123 |
| N | -3.9019 | -0.8325 | -1.1221 |
| H | -3.8326 | -0.3324 | 0.9598 |
| H | -1.2379 | -1.031 | 1.3742 |
| H | -1.3456 | 2.2293 | -1.3745 |
| H | 2.2532 | -0.3302 | 0.5453 |
| H | -5.8752 | 1.5987 | -1.7998 |
| H | -0.1319 | -1.8894 | 0.8336 |
| H | 1.2974 | -1.9234 | 1.9568 |
| H | -0.7851 | -1.2462 | 3.1557 |
| H | 0.4293 | 0.1052 | 3.1223 |
| H | -1. | 0.1392 | 1.9992 |
| H | -5.8071 | -1.9168 | -2.2176 |
| H | -6.7361 | -0.3765 | -2.4858 |
| H | -3.8237 | -1.8703 | -1.2163 |
| H | -3.1337 | -0.2899 | -0.6668 |

**Coordinates of 1a:**

**Charge = 0 Multiplicity = 1**

| | | | |
|---|---|---|---|
| C | -2.9499 | 0.3472 | 0.7416 |
| C | -1.5991 | -0.0165 | 0.9574 |
| C | -0.8207 | 0.8245 | 0.1937 |
| N | -1.6545 | 1.6824 | -0.475 |
| C | -2.9548 | 1.399 | -0.1472 |
| C | 0.528 | 0.807 | 0.1171 |
| C | -4.0491 | 2.0391 | -0.6141 |
| O | 1.1031 | 1.5987 | -0.5912 |
| O | -4.1395 | 3.2388 | -0.5052 |
| N | 1.243 | -0.1081 | 0.8421 |
| N | -5.0555 | 1.3282 | -1.2106 |
| C | 0.5527 | -1.0584 | 1.6923 |
| C | -0.3838 | -1.9108 | 0.8462 |
| C | -4.9549 | -0.0075 | -1.3319 |
| N | -5.8856 | -0.665 | -1.8835 |
| N | -3.9343 | -0.6045 | -0.8964 |
| H | -3.8326 | -0.1234 | 1.1992 |
| H | -1.2379 | -0.8219 | 1.6137 |
| H | -1.3456 | 2.4383 | -1.1351 |
| H | 2.2532 | -0.1212 | 0.7847 |
| H | -5.8752 | 1.8077 | -1.5603 |
| H | -0.0379 | -0.5088 | 2.459 |
| H | 1.2974 | -1.7143 | 2.1963 |
| H | -0.9137 | -2.6403 | 1.4987 |
| H | 0.2068 | -2.4605 | 0.0794 |
| H | -1.1285 | -1.255 | 0.3422 |
| H | -5.8071 | -1.7077 | -1.9781 |
| H | -6.7361 | -0.1675 | -2.2463 |
| H | -3.8578 | -1.6195 | -0.9886 |

**Coordinates of 1b:**

**Charge = 0 Multiplicity = 1**

| | | | |
|---|---|---|---|
| C | -2.3584 | 0.1929 | 0.1154 |
| C | -1.0076 | -0.1708 | 0.3312 |
| C | -0.2292 | 0.6702 | -0.4325 |
| N | -1.063 | 1.5281 | -1.1012 |
| C | -2.3633 | 1.2447 | -0.7734 |
| C | 1.1195 | 0.6526 | -0.5091 |
| C | -3.56424 | 2.23825 | -0.84315 |
| O | 1.6946 | 1.4444 | -1.2173 |
| O | -3.3357 | 2.7985 | -2.0209 |
| N | 1.8345 | -0.2624 | 0.216 |
| N | -4.6053 | 1.5287 | -0.8613 |
| C | 1.1442 | -1.2128 | 1.0662 |
| C | 0.2077 | -2.0652 | 0.22 |
| C | -4.7324 | 0.5756 | -0.047 |
| N | -5.72383 | 0.54317 | 0.73957 |
| N | -3.86874 | -0.34944 | -0.01543 |
| H | -3.2411 | -0.2777 | 0.5731 |
| H | -0.6464 | -0.9763 | 0.9876 |
| H | -0.754 | 2.284 | -1.7612 |
| H | 2.8448 | -0.2755 | 0.1586 |
| H | 0.5536 | -0.6631 | 1.8329 |
| H | 1.8889 | -1.8686 | 1.5701 |
| H | -0.3222 | -2.7947 | 0.8726 |
| H | 0.7983 | -2.6148 | -0.5467 |
| H | -0.537 | -1.4093 | -0.2839 |
| H | -5.82983 | -0.2511 | 1.4182 |
| H | -6.44015 | 1.31043 | 0.71345 |
| H | -3.97466 | -1.14364 | 0.66307 |
| H | -3.04654 | -0.32249 | -0.66791 |

**Coordinates of 2:**

Charge = 1 Multiplicity = 1

| | | | |
|---|---|---|---|
| C | -2.2129 | 3.3245 | -0.4147 |
| C | -1.237 | 2.3311 | -0.4626 |
| C | -1.5313 | 1.0126 | -0.1015 |
| C | -2.8398 | 0.7307 | 0.3086 |
| C | -3.837 | 1.7047 | 0.3665 |
| C | -3.5167 | 3.0254 | -0.0021 |
| N | -3.409 | -0.4553 | 0.7148 |
| C | -4.7292 | -0.265 | 1.0269 |
| C | -5.0228 | 1.0637 | 0.8207 |
| C | -5.5895 | -1.2125 | 1.4599 |
| O | -6.7361 | -0.9211 | 1.7039 |
| N | -5.1617 | -2.5028 | 1.622 |
| C | -3.885 | -2.8272 | 1.3502 |
| N | -3.4788 | -4.0525 | 1.5042 |
| N | -3.0789 | -1.9393 | 0.9445 |
| C | 0.0136 | 2.6468 | -0.8643 |
| O | 0.4227 | 3.7761 | -0.7347 |
| N | 0.8174 | 1.687 | -1.4183 |
| C | 0.3264 | 0.3316 | -1.5739 |
| C | 1.4082 | -0.5311 | -2.2104 |
| H | -1.9562 | 4.3542 | -0.704 |
| H | -0.7631 | 0.2261 | -0.1383 |
| H | -4.2829 | 3.8138 | 0.0338 |
| H | -2.9038 | -1.3737 | 0.7765 |
| H | -5.9997 | 1.544 | 0.9789 |
| H | -5.8061 | -3.2125 | 1.9463 |
| H | -2.4869 | -4.3045 | 1.2931 |
| H | -4.1442 | -4.7854 | 1.8391 |
| H | -2.0822 | -2.1926 | 0.7324 |
| H | -3.407 | -0.9497 | 0.8202 |
| H | 1.7542 | 1.9235 | -1.7193 |
| H | 0.0613 | -0.0833 | -0.5757 |
| H | -0.5755 | 0.3364 | -2.2261 |
| H | 1.0314 | -1.5715 | -2.3298 |
| H | 1.6733 | -0.1162 | -3.2086 |
| H | 2.3101 | -0.5359 | -1.5582 |

**Coordinates of 2a:**

Charge = 0 Multiplicity = 1

| | | | |
|---|---|---|---|
| C | 0.50890000 | 2.17260000 | -0.58390000 |
| C | 1.48480000 | 1.17920000 | -0.63180000 |
| C | 1.19050000 | -0.13930000 | -0.27070000 |
| C | -0.11800000 | -0.42120000 | 0.13940000 |
| C | -1.11520000 | 0.55280000 | 0.19730000 |
| C | -0.79490000 | 1.87350000 | -0.17130000 |
| N | -0.68720000 | -1.60720000 | 0.54560000 |
| C | -2.00740000 | -1.41690000 | 0.85770000 |
| C | -2.30100000 | -0.08820000 | 0.65150000 |
| C | -2.86770000 | -2.36440000 | 1.29060000 |
| O | -4.01430000 | -2.07310000 | 1.53470000 |
| N | -2.43990000 | -3.65470000 | 1.45280000 |
| C | -3.29640000 | -4.59800000 | 1.88380000 |
| N | -2.90270000 | -5.78560000 | 2.03300000 |
| N | -4.49810000 | -4.29270000 | 2.13950000 |
| C | 2.73540000 | 1.49490000 | -1.03360000 |
| O | 3.58890000 | 0.64120000 | -1.07870000 |
| N | 3.03550000 | 2.78230000 | -1.38950000 |
| C | 4.37780000 | 3.12110000 | -1.82070000 |
| C | 4.44440000 | 4.60520000 | -2.15650000 |
| H | 0.76560000 | 3.20230000 | -0.87320000 |
| H | 1.95860000 | -0.92580000 | -0.30750000 |
| H | -1.56120000 | 2.66190000 | -0.13540000 |
| H | -0.18200000 | -2.52560000 | 0.60730000 |
| H | -3.27790000 | 0.39210000 | 0.80970000 |
| H | -1.47930000 | -3.89880000 | 1.24830000 |
| H | -3.67280000 | -6.37020000 | 2.36410000 |
| H | -5.16670000 | -5.02910000 | 2.47600000 |
| H | -4.82620000 | -3.30300000 | 2.01520000 |
| H | 2.32050000 | 3.49750000 | -1.35170000 |
| H | 5.09800000 | 2.89350000 | -1.00330000 |
| H | 4.63900000 | 2.52460000 | -2.72340000 |
| H | 5.47480000 | 4.86530000 | -2.48750000 |
| H | 3.72420000 | 4.83280000 | -2.97390000 |
| H | 4.18320000 | 5.20170000 | -1.25390000 |

**Coordinates of 2b:**

Charge = 0 Multiplicity = 1

| | | | |
|---|---|---|---|
| C | 0.60460000 | 2.17880000 | -0.59320000 |
| C | 1.58050000 | 1.18540000 | -0.64110000 |
| C | 1.28620000 | -0.13310000 | -0.28000000 |
| C | -0.02230000 | -0.41500000 | 0.13010000 |
| C | -1.01950000 | 0.55900000 | 0.18800000 |
| C | -0.69920000 | 1.87970000 | -0.18060000 |
| N | -0.59150000 | -1.60100000 | 0.53630000 |
| C | -1.91170000 | -1.41070000 | 0.84840000 |
| C | -2.20530000 | -0.08200000 | 0.64220000 |
| C | -2.77190000 | -2.35820000 | 1.28130000 |
| O | -3.91860000 | -2.06680000 | 1.52540000 |
| N | -2.37820000 | -3.54580000 | 1.43050000 |
| C | -3.25750000 | -4.35100000 | 1.83800000 |
| N | -2.96450000 | -5.56900000 | 2.02070000 |
| N | -4.43400000 | -3.94210000 | 2.06490000 |
| C | 2.83120000 | 1.50110000 | -1.04290000 |
| O | 3.68460000 | 0.64740000 | -1.08800000 |
| N | 3.13120000 | 2.78850000 | -1.39890000 |
| C | 4.47350000 | 3.12740000 | -1.83010000 |
| C | 4.54020000 | 4.61140000 | -2.16580000 |
| H | 0.86130000 | 3.20860000 | -0.88260000 |
| H | 2.05440000 | -0.91960000 | -0.31690000 |
| H | -1.46540000 | 2.66810000 | -0.14470000 |
| H | -0.08620000 | -2.51940000 | 0.59800000 |
| H | -3.18220000 | 0.39830000 | 0.80030000 |
| H | -3.69720000 | -6.24010000 | 2.36030000 |
| H | -1.98870000 | -5.90820000 | 1.83250000 |
| H | -5.16670000 | -4.61310000 | 2.40450000 |
| H | -4.67700000 | -2.93190000 | 1.91340000 |
| H | 2.41620000 | 3.50370000 | -1.36110000 |
| H | 5.19380000 | 2.89980000 | -1.01260000 |
| H | 4.73470000 | 2.53090000 | -2.73270000 |
| H | 5.57050000 | 4.87150000 | -2.49680000 |
| H | 3.81990000 | 4.83900000 | -2.98330000 |
| H | 4.27900000 | 5.20790000 | -1.26320000 |

**Coordinates of 3:**

Charge = 1 Multiplicity = 1

| | | | |
|---|---|---|---|
| C | -2.6231 | 2.713 | 0.0432 |
| C | -1.6472 | 1.7196 | -0.0047 |
| C | -1.9415 | 0.4011 | 0.3564 |
| C | -3.25 | 0.1192 | 0.7665 |
| C | -4.2472 | 1.0932 | 0.8244 |
| C | -3.9269 | 2.4139 | 0.4558 |
| N | -3.8192 | -1.0668 | 1.1727 |
| C | -5.1394 | -0.8765 | 1.4848 |
| C | -5.433 | 0.4522 | 1.2786 |
| C | -5.9996 | -1.824 | 1.9177 |
| O | -6.7361 | -1.5921 | 2.8467 |
| N | -6.0367 | -3.047 | 1.3036 |
| C | -5.2167 | -3.3052 | 0.2692 |
| N | -5.2519 | -4.4665 | -0.314 |
| N | -4.4106 | -2.4173 | -0.1365 |
| C | -0.3965 | 2.0353 | -0.4065 |
| O | 0.0125 | 3.1645 | -0.2769 |
| N | 0.4072 | 1.0755 | -0.9605 |
| C | -0.0837 | -0.28 | -1.1161 |
| C | 0.9981 | -1.1426 | -1.7525 |
| O | -2.3068 | 3.9814 | -0.3133 |
| C | -3.3186 | 4.9404 | -0.4631 |
| H | -1.1733 | -0.3854 | 0.3195 |
| H | -4.6931 | 3.2023 | 0.4917 |
| H | -3.3139 | -1.9852 | 1.2344 |
| H | -6.4098 | 0.9325 | 1.4367 |
| H | -6.6811 | -3.7567 | 1.6279 |
| H | -4.6148 | -4.6672 | -1.1176 |
| H | -5.9173 | -5.1994 | 0.0209 |
| H | -3.7704 | -2.6189 | -0.944 |
| H | -4.3821 | -1.4793 | 0.3345 |
| H | 1.3441 | 1.312 | -1.2614 |
| H | -0.3488 | -0.6948 | -0.1179 |
| H | -0.9856 | -0.2751 | -1.7682 |
| H | 0.6212 | -2.183 | -1.8719 |
| H | 1.2631 | -0.7278 | -2.7507 |
| H | 1.9 | -1.1474 | -1.1003 |
| H | -2.8668 | 5.9143 | -0.7567 |
| H | -4.0294 | 4.6087 | -1.2527 |
| H | -3.8624 | 5.0594 | 0.5007 |

**Coordinates of 3a:**

Charge = 0 Multiplicity = 1

| | | | |
|---|---|---|---|
| C | -2.2129 | 2.6047 | -0.2682 |
| C | -1.237 | 1.6112 | -0.3161 |
| C | -1.5313 | 0.2927 | 0.045 |
| C | -2.8398 | 0.0109 | 0.4551 |
| C | -3.837 | 0.9848 | 0.513 |
| C | -3.5167 | 2.3055 | 0.1444 |
| N | -3.409 | -1.1752 | 0.8613 |
| C | -4.7292 | -0.9849 | 1.1734 |
| C | -5.0228 | 0.3439 | 0.9672 |
| C | -5.80229 | -2.27512 | 1.01608 |
| O | -6.7361 | -1.641 | 1.8504 |
| N | -5.1617 | -3.2226 | 1.7685 |
| C | -3.885 | -3.5471 | 1.4967 |
| N | -3.61282 | -4.67348 | 1.00213 |
| N | -2.9568 | -2.72066 | 1.73793 |
| C | 0.0136 | 1.927 | -0.7179 |
| O | 0.4227 | 3.0562 | -0.5883 |
| N | 0.8174 | 0.9672 | -1.2718 |
| C | 0.3264 | -0.3883 | -1.4274 |
| C | 1.4082 | -1.2509 | -2.0639 |
| O | -1.8967 | 3.8731 | -0.6246 |
| C | -2.9084 | 4.8321 | -0.7745 |
| H | -0.7631 | -0.4937 | 0.0082 |
| H | -4.2829 | 3.094 | 0.1803 |
| H | -2.9038 | -2.0935 | 0.923 |
| H | -5.9997 | 0.8242 | 1.1254 |
| H | -5.8061 | -3.9324 | 2.0928 |
| H | -2.64274 | -4.92006 | 0.79575 |
| H | -1.9601 | -2.97393 | 1.52589 |
| H | -3.18363 | -1.78193 | 2.15016 |
| H | 1.7542 | 1.2037 | -1.5728 |
| H | 0.0613 | -0.8031 | -0.4292 |
| H | -0.5755 | -0.3834 | -2.0796 |
| H | 1.0314 | -2.2914 | -2.1833 |
| H | 1.6733 | -0.8361 | -3.0621 |
| H | 2.3101 | -1.2558 | -1.4117 |
| H | -2.4567 | 5.806 | -1.0681 |
| H | -3.4515 | 4.9513 | 0.1897 |
| H | -3.6198 | 4.5003 | -1.5635 |

**Coordinates of 3b:**

Charge = 0 Multiplicity = 1

| | | | |
|---|---|---|---|
| C | -2.2129 | 2.62 | -0.2657 |
| C | -1.237 | 1.6266 | -0.3136 |
| C | -1.5313 | 0.3081 | 0.0475 |
| C | -2.8398 | 0.0262 | 0.4576 |
| C | -3.837 | 1.0002 | 0.5155 |
| C | -3.5167 | 2.3209 | 0.1469 |
| N | -3.409 | -1.1598 | 0.8638 |
| C | -4.7292 | -0.9695 | 1.1759 |
| C | -5.0228 | 0.3592 | 0.9697 |
| C | -5.74123 | -2.13866 | 1.16042 |
| O | -6.7361 | -1.6257 | 1.8529 |
| N | -5.1958 | -3.1046 | 1.758 |
| C | -3.9998 | -3.4085 | 1.5035 |
| N | -3.70955 | -4.58567 | 1.13918 |
| N | -3.08824 | -2.53661 | 1.61206 |
| C | 0.0136 | 1.9423 | -0.7154 |
| O | 0.4227 | 3.0715 | -0.5858 |
| N | 0.8174 | 0.9825 | -1.2694 |
| C | 0.3264 | -0.373 | -1.4249 |
| C | 1.4082 | -1.2356 | -2.0614 |
| O | -1.8967 | 3.8884 | -0.6221 |
| C | -2.9084 | 4.8474 | -0.772 |
| H | -0.7631 | -0.4784 | 0.0107 |
| H | -4.2829 | 3.1093 | 0.1828 |
| H | -2.9038 | -2.0782 | 0.9255 |
| H | -5.9997 | 0.8395 | 1.1278 |
| H | -2.71285 | -4.83896 | 0.92706 |
| H | -4.46554 | -5.3088 | 1.04916 |
| H | -2.09162 | -2.78985 | 1.39985 |
| H | -3.32895 | -1.56028 | 1.91415 |
| H | 1.7542 | 1.219 | -1.5703 |
| H | 0.0613 | -0.7878 | -0.4267 |
| H | -0.5755 | -0.3681 | -2.0771 |
| H | 1.0314 | -2.2761 | -2.1808 |
| H | 1.6733 | -0.8208 | -3.0596 |
| H | 2.3101 | -1.2405 | -1.4092 |
| H | -2.4567 | 5.8213 | -1.0656 |
| H | -3.4515 | 4.9666 | 0.1922 |
| H | -3.6198 | 4.5156 | -1.561 |

## 10. NMR Spectra

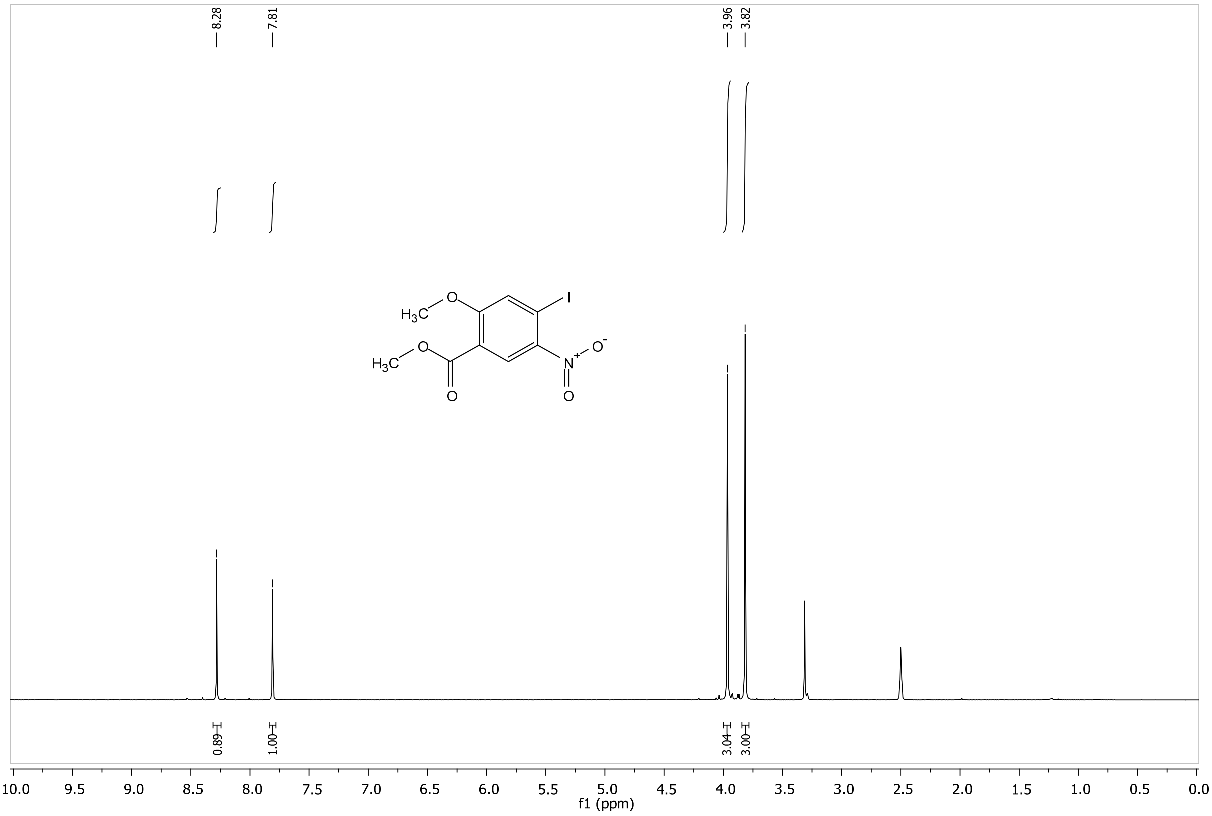


**Figure S55.** $^{1}$H-NMR spectrum of **B** (600 MHz, DMSO-$d_6$).

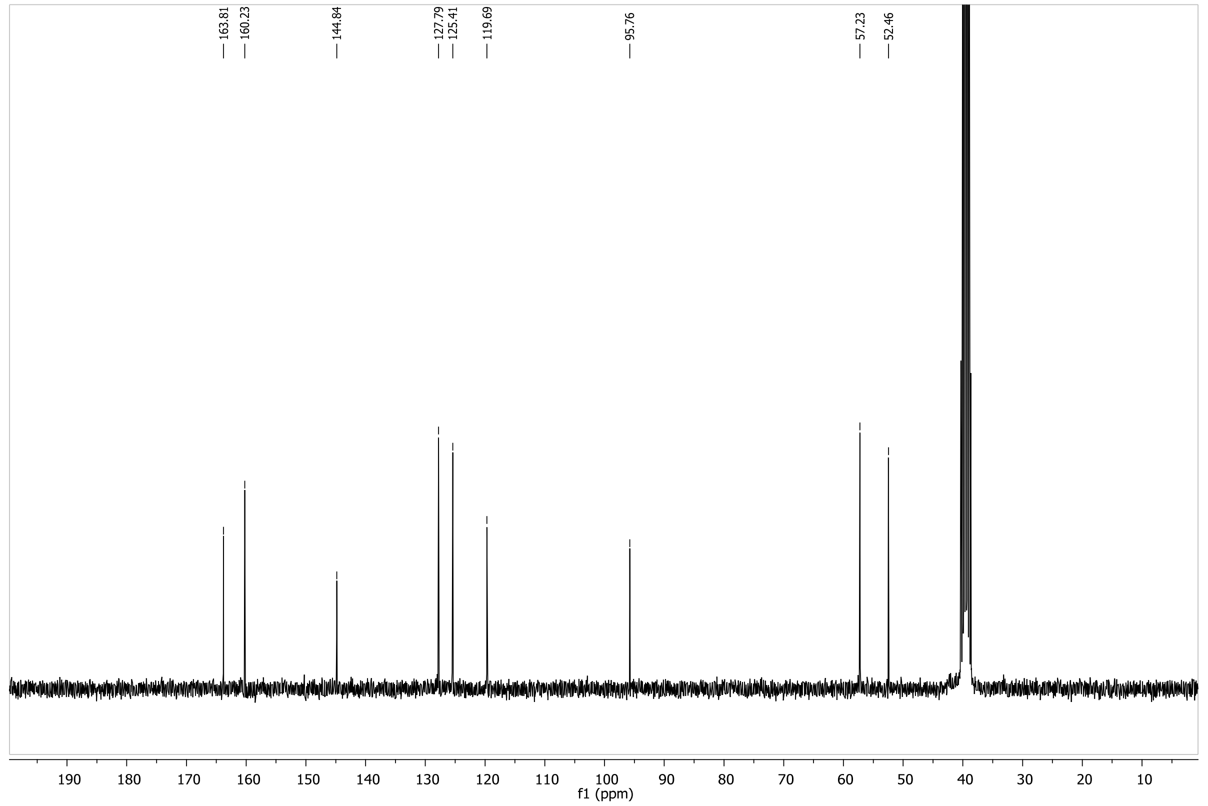


**Figure S56.** $^{13}$C-NMR spectrum of **B** (75 MHz, DMSO-$d_6$).

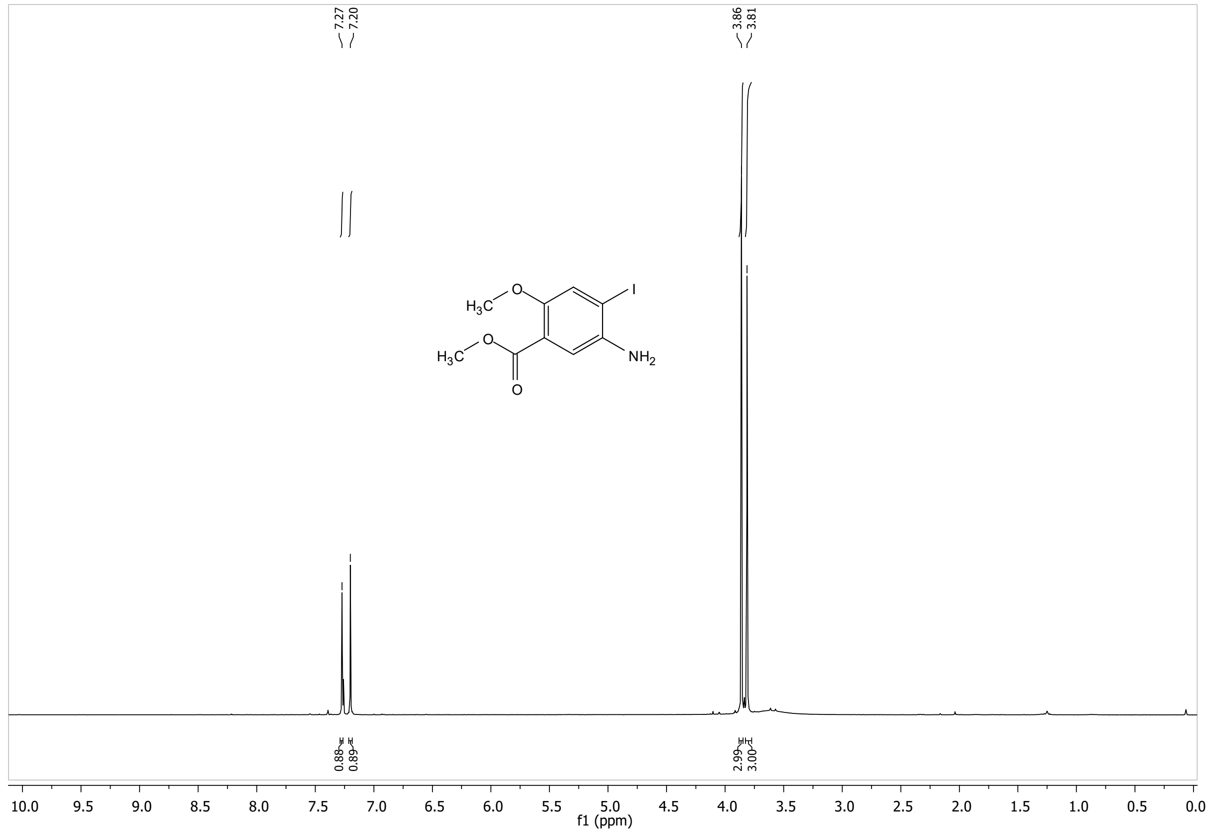

**Figure S57.** $^{1}$H-NMR spectrum of **C** (300 MHz, $CDCl_3$).

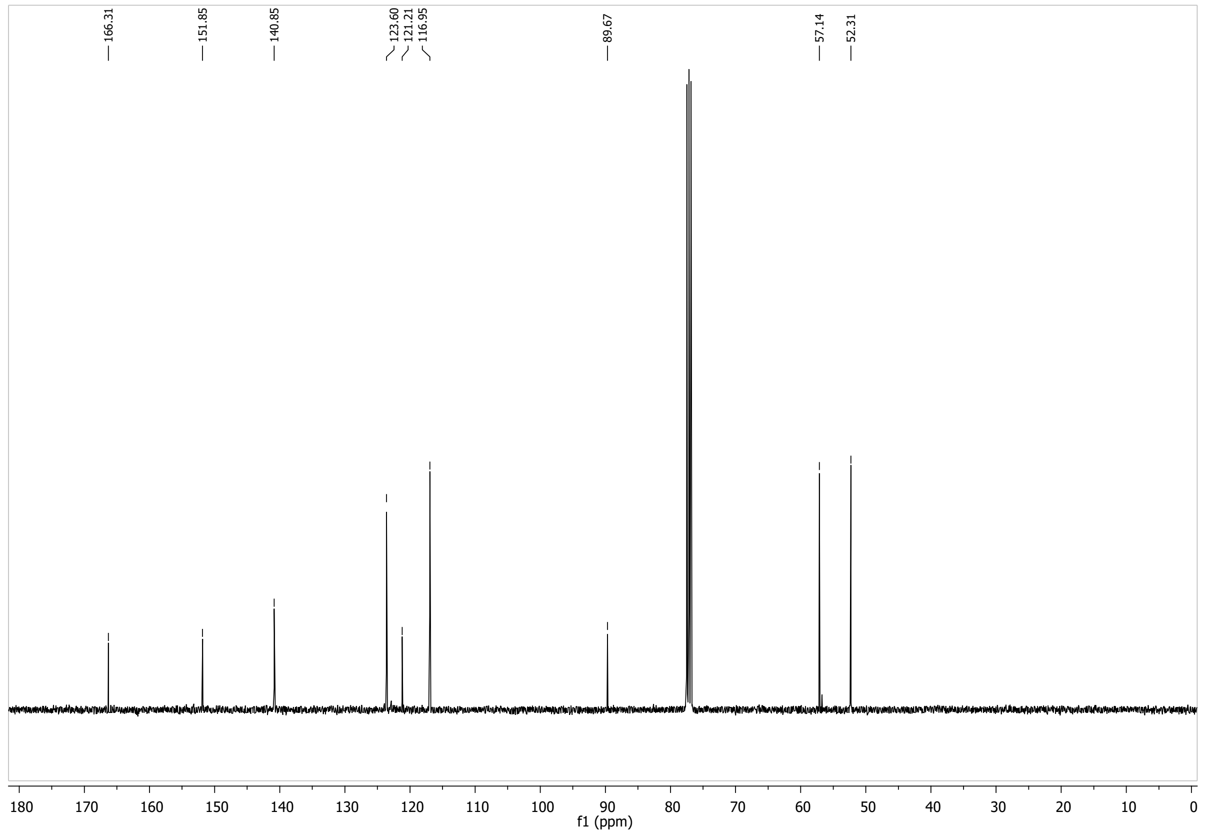

**Figure S58.** $^{13}$C-NMR spectrum of **C** (101 MHz, $CDCl_3$).

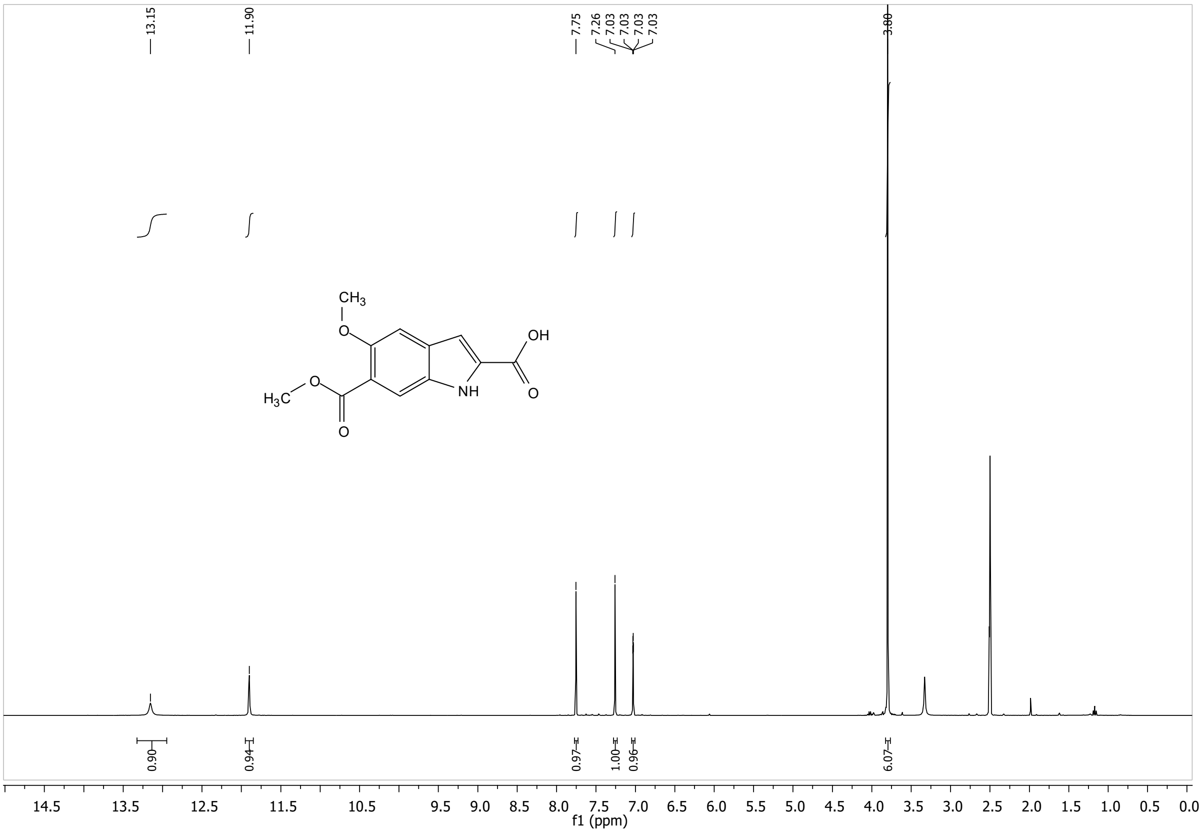


**Figure S59.** $^{1}$H-NMR spectrum of **D** (400 MHz, DMSO-$d_6$).

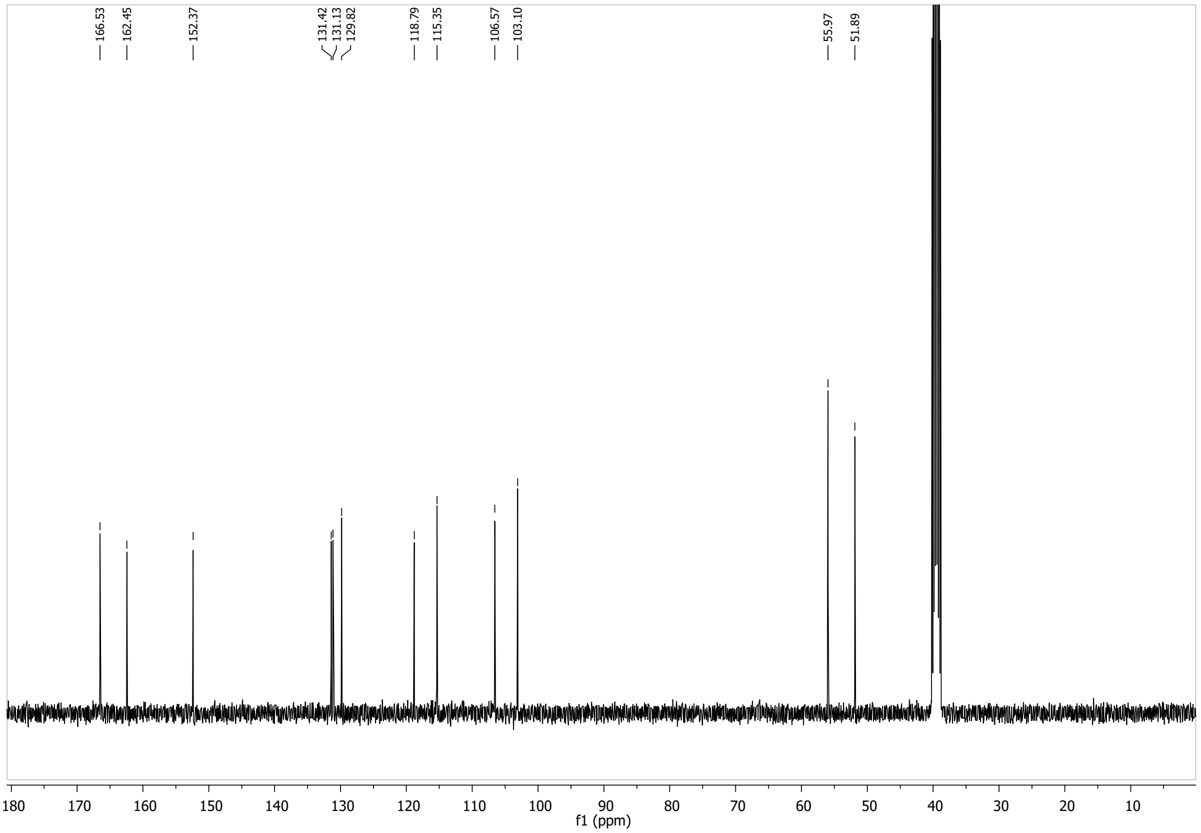


**Figure S60.** $^{13}$C-NMR spectrum of **D** (101 MHz, DMSO-$d_6$).

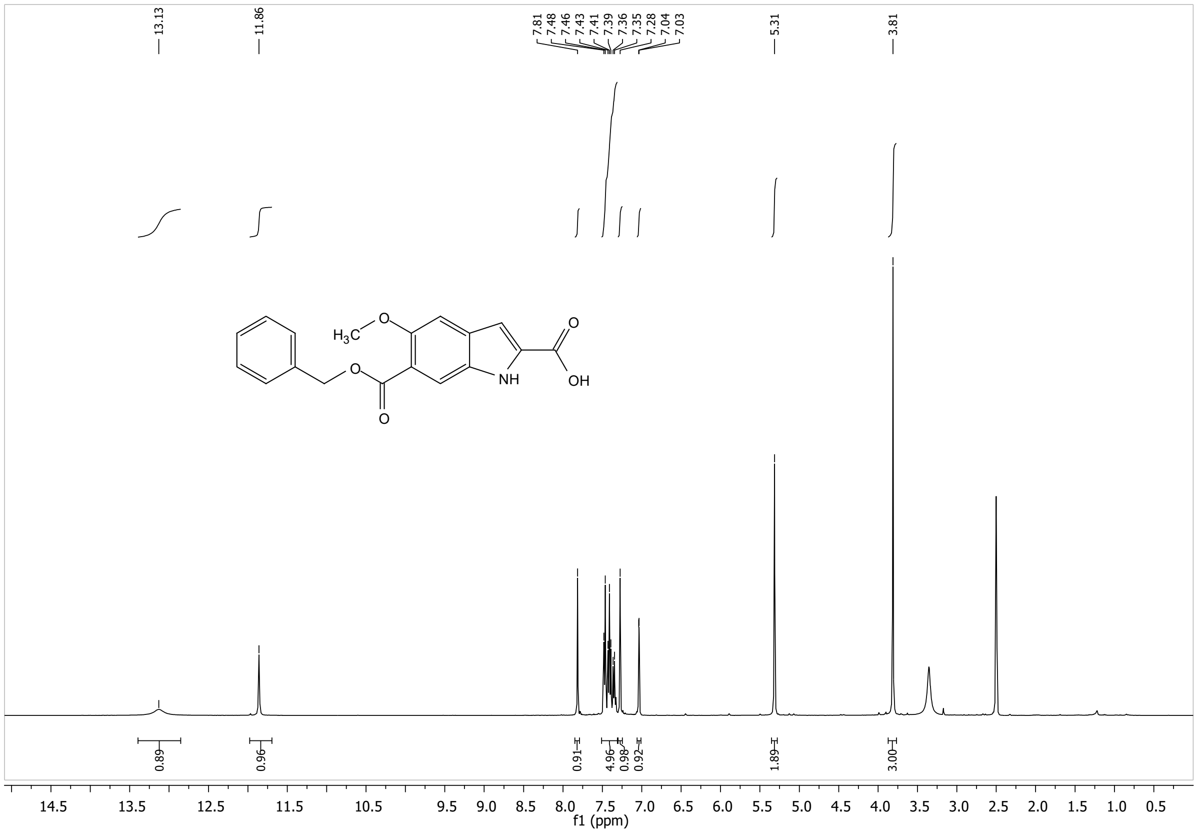

**Figure S61.** $^{1}$H-NMR spectrum of **E** (400 MHz, DMSO-$d_6$).

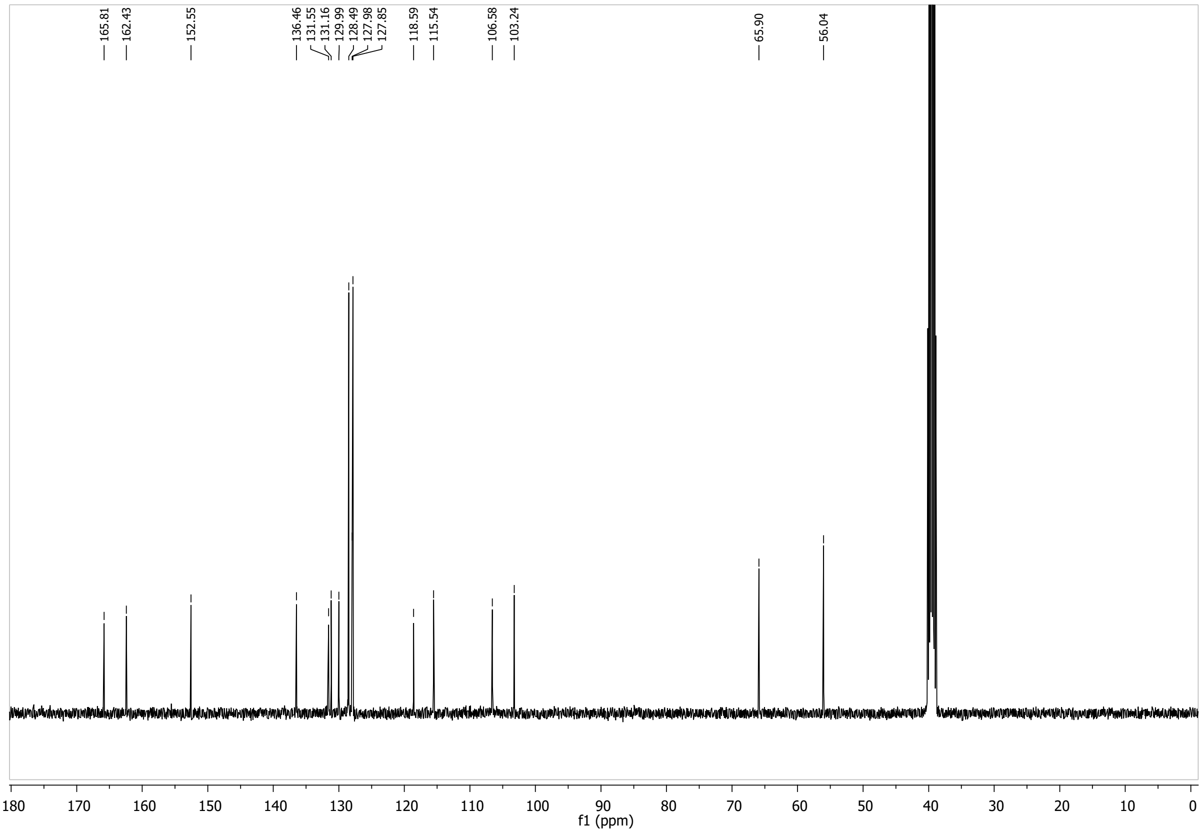

**Figure S62.** $^{13}$C-NMR spectrum of **E** (101 MHz, DMSO-$d_6$).

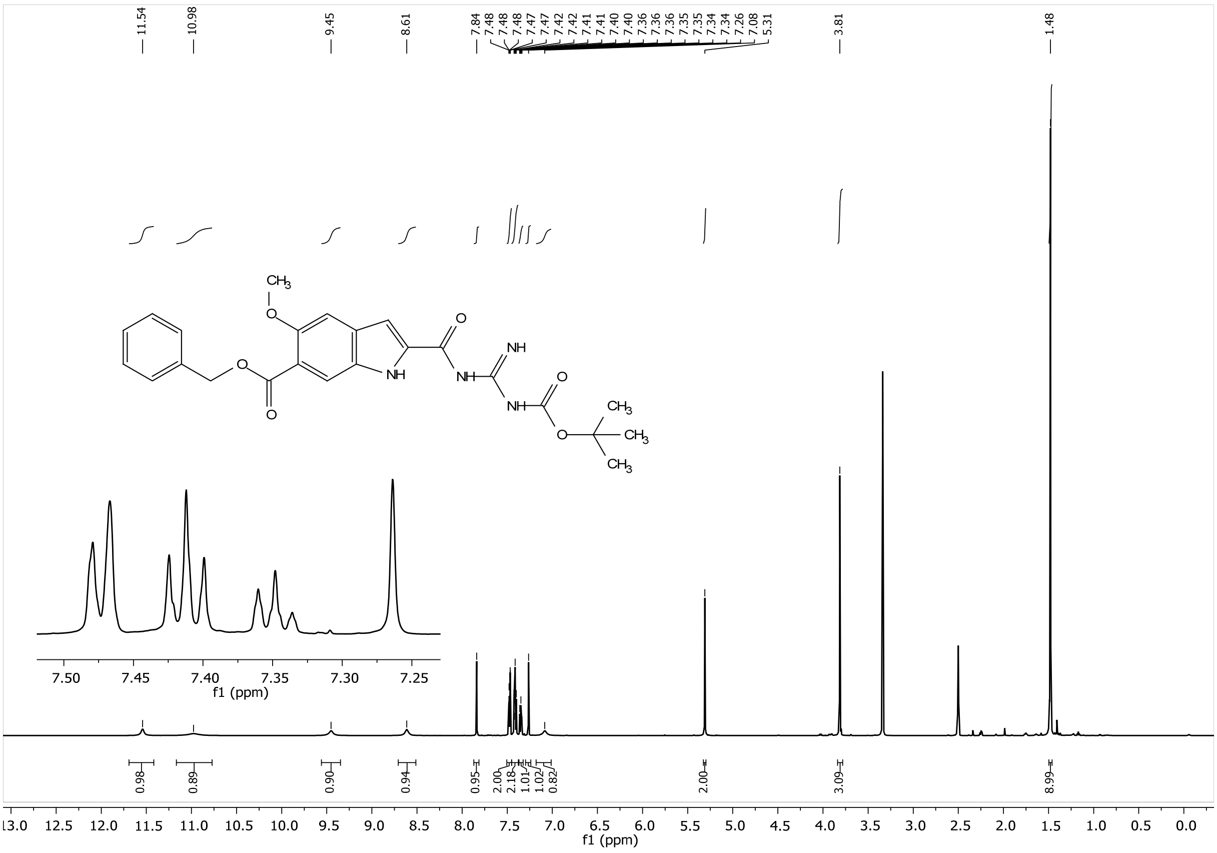

**Figure S63.** $^{1}$H-NMR spectrum of **G** (600 MHz, DMSO-$d_6$). The product was sufficiently pure to be used in the next step.

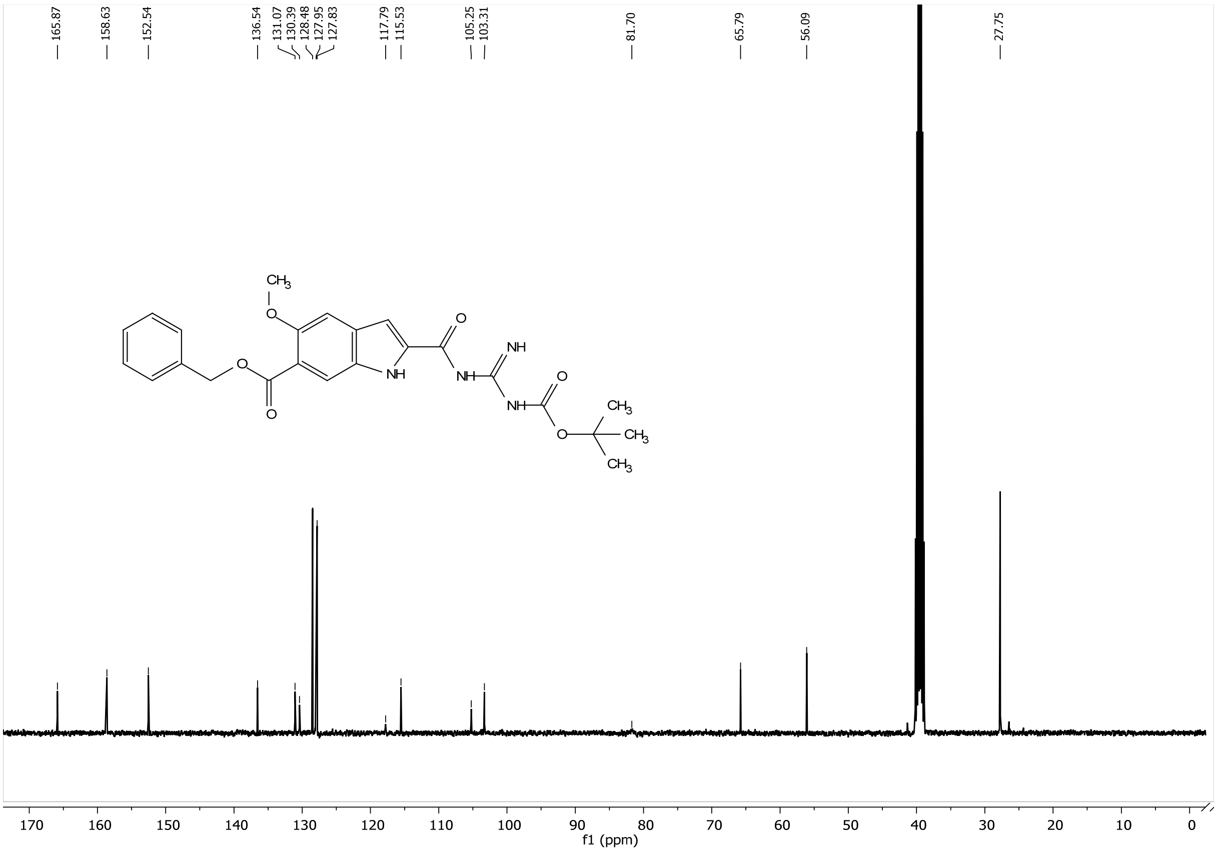

**Figure S64.** $^{13}$C-NMR spectrum of **G** (101 MHz, DMSO-$d_6$).

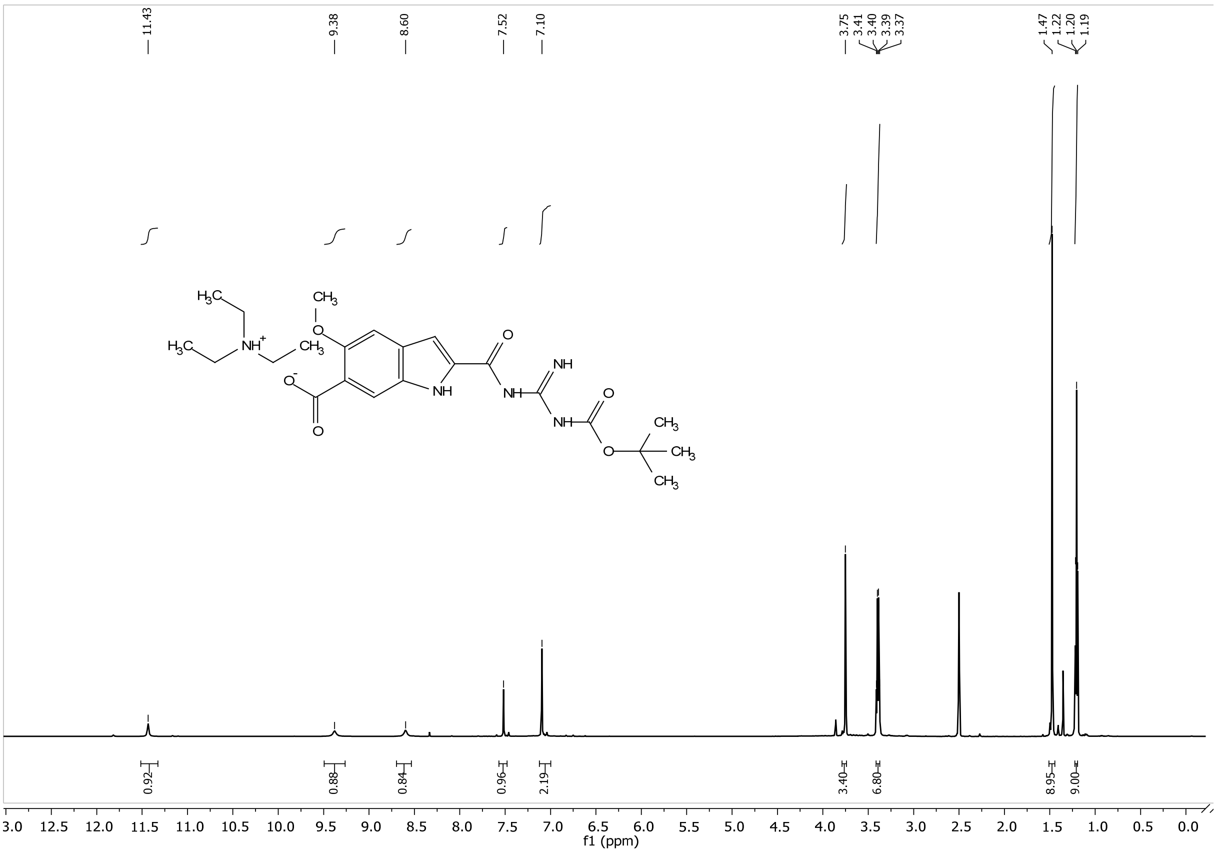


**Figure S65.** $^{1}$H-NMR spectrum of **H** (600 MHz, DMSO-*d$_6$*). The product was sufficiently pure to be used in the next step.

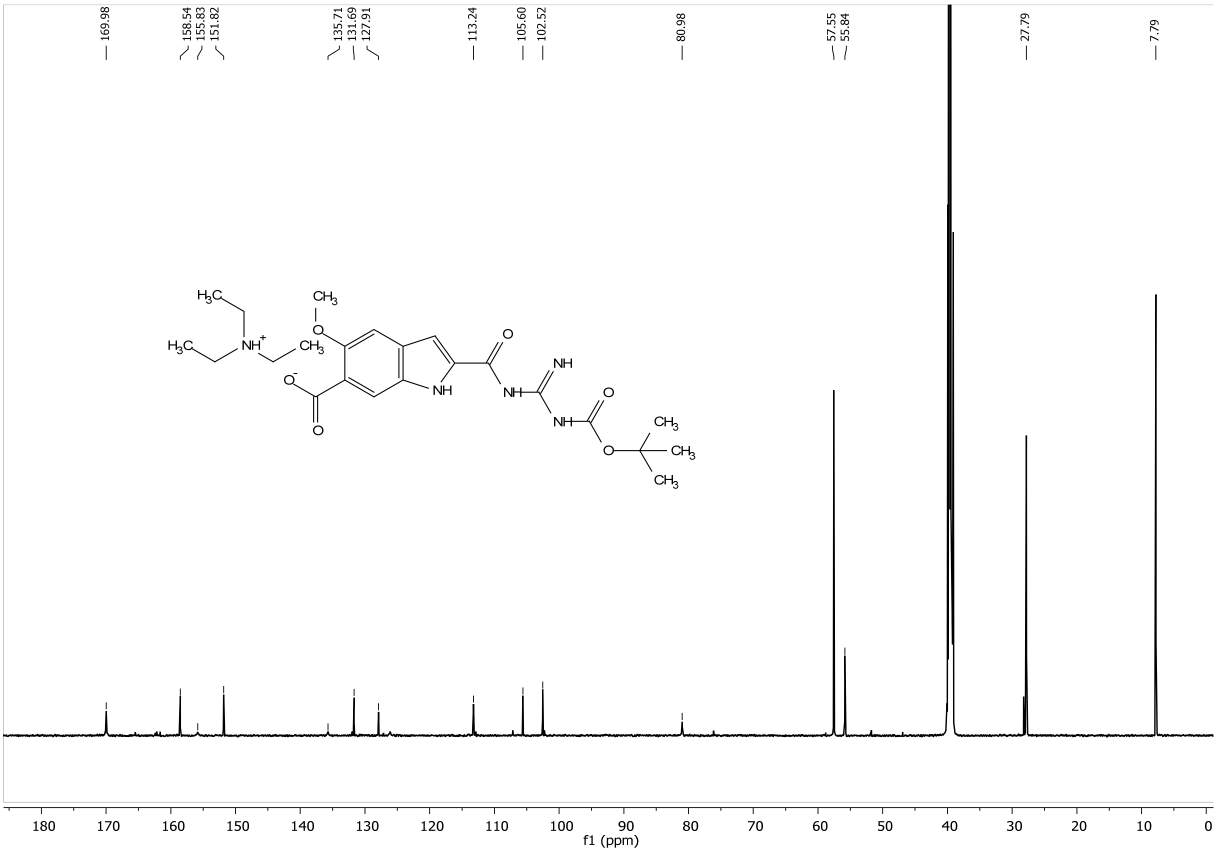


**Figure S66.** $^{13}$C-NMR spectrum of **H** (151 MHz, DMSO-*d$_6$*).

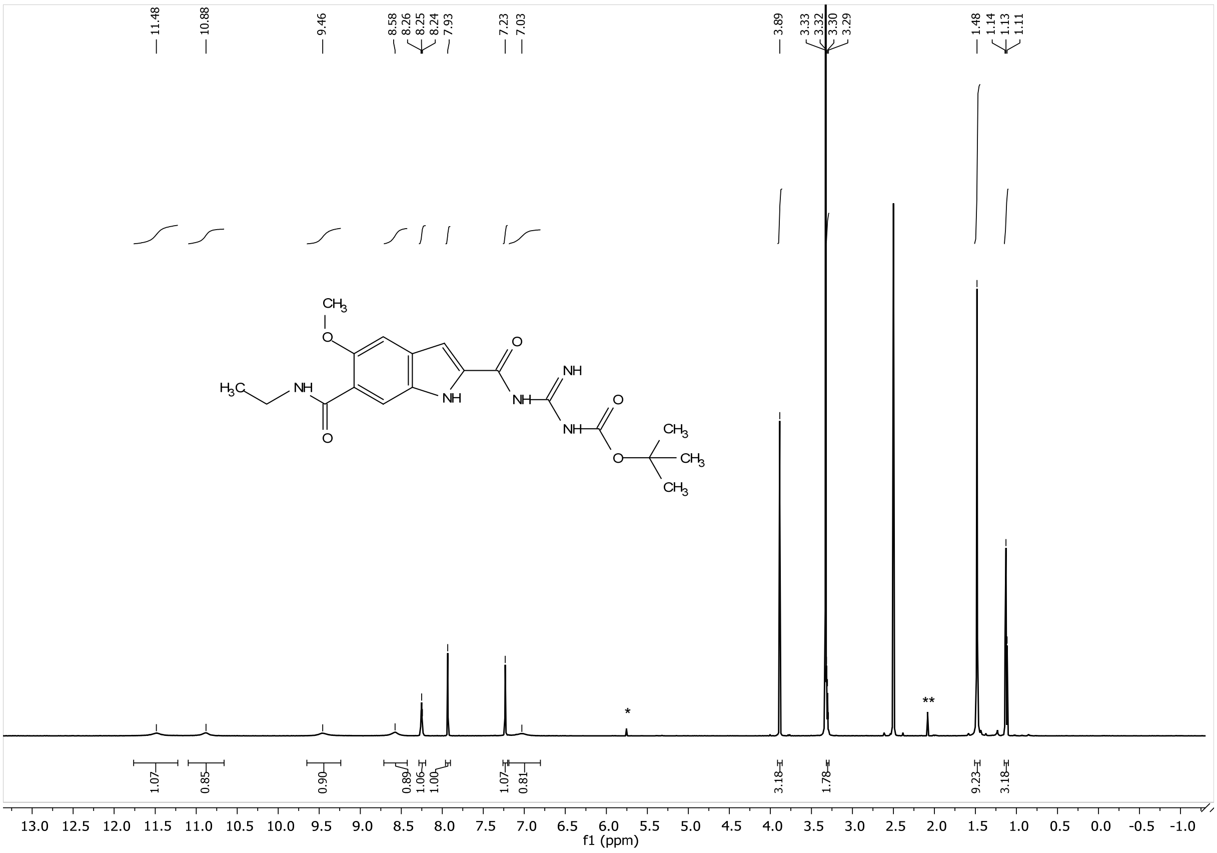


**Figure S67.** $^{1}$H-NMR spectrum of **I** (600 MHz, DMSO-$d_6$).*DCM, **acetone

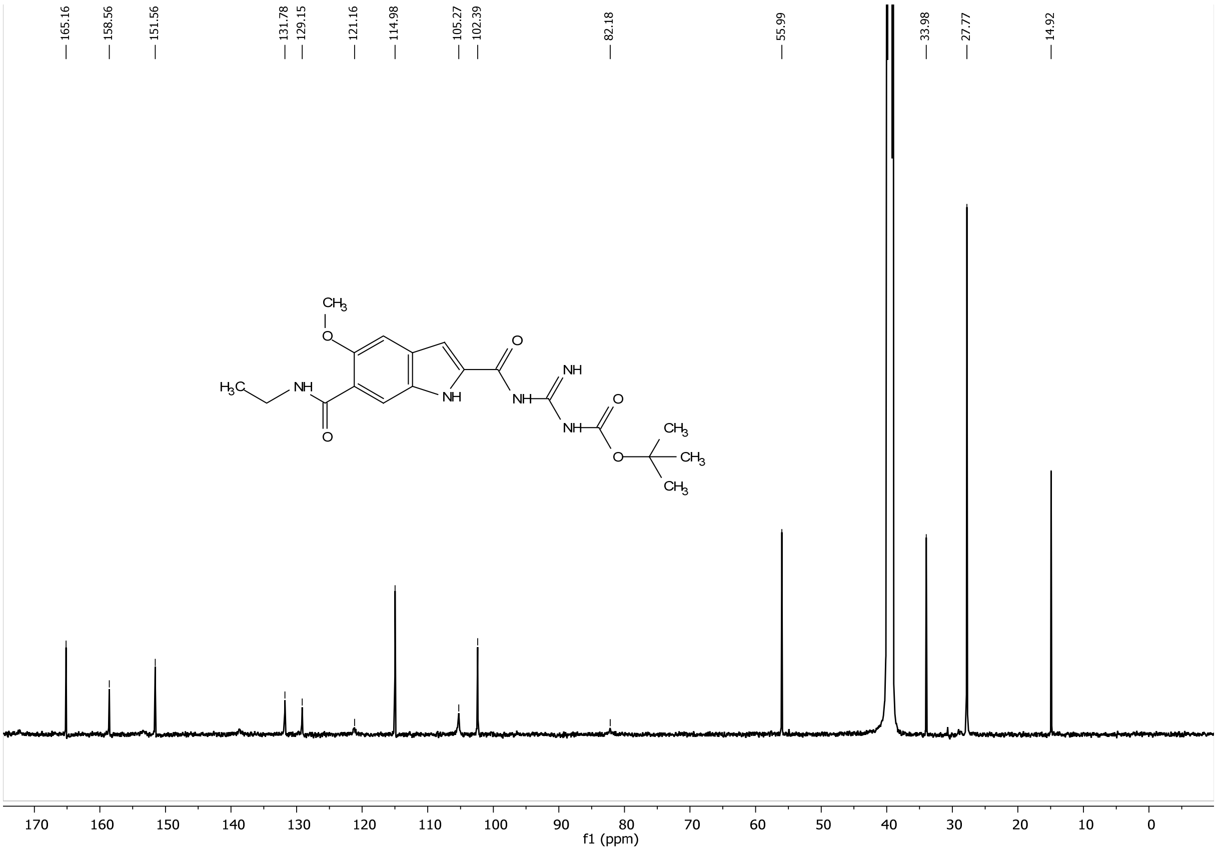


**Figure S68.** $^{13}$C-NMR spectrum of **I** (151 MHz, DMSO-$d_6$).

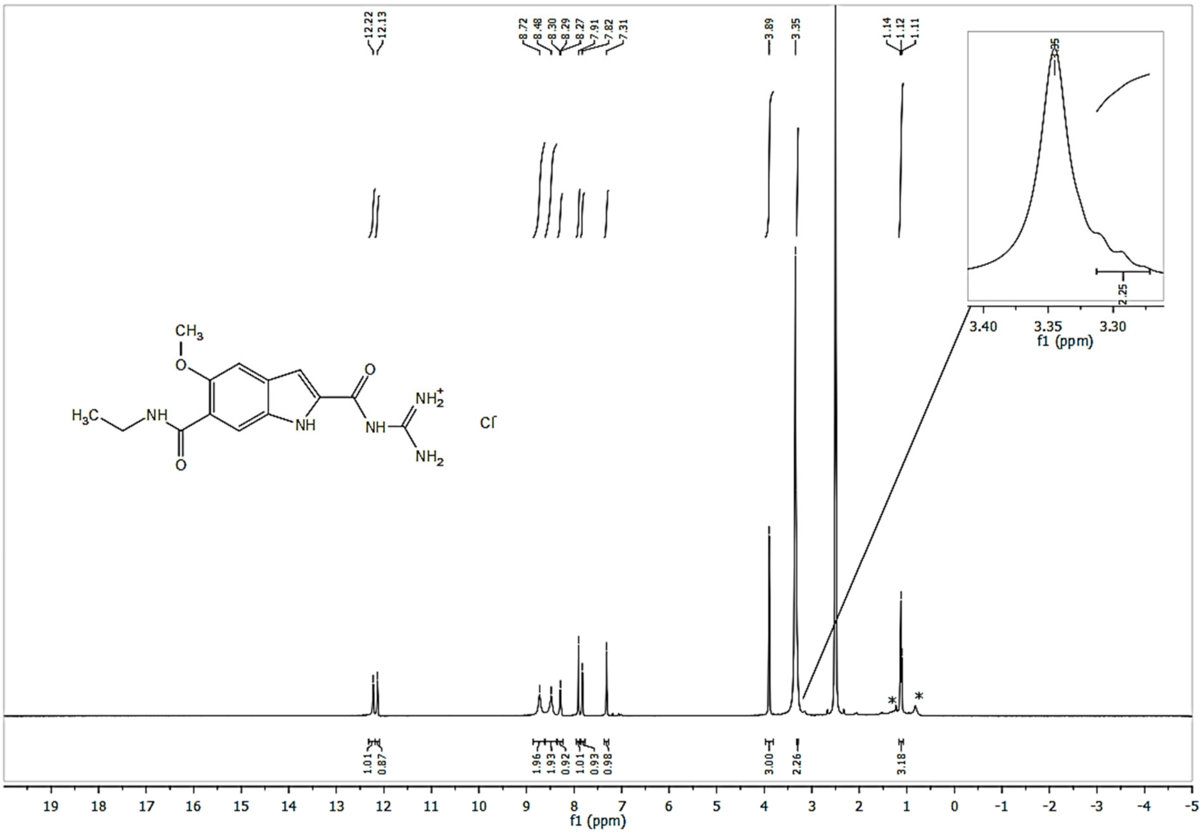

**Figure S69.** $^{1}$H-NMR spectrum of **3** (600 MHz, DMSO-$d_6$). *H-grease.

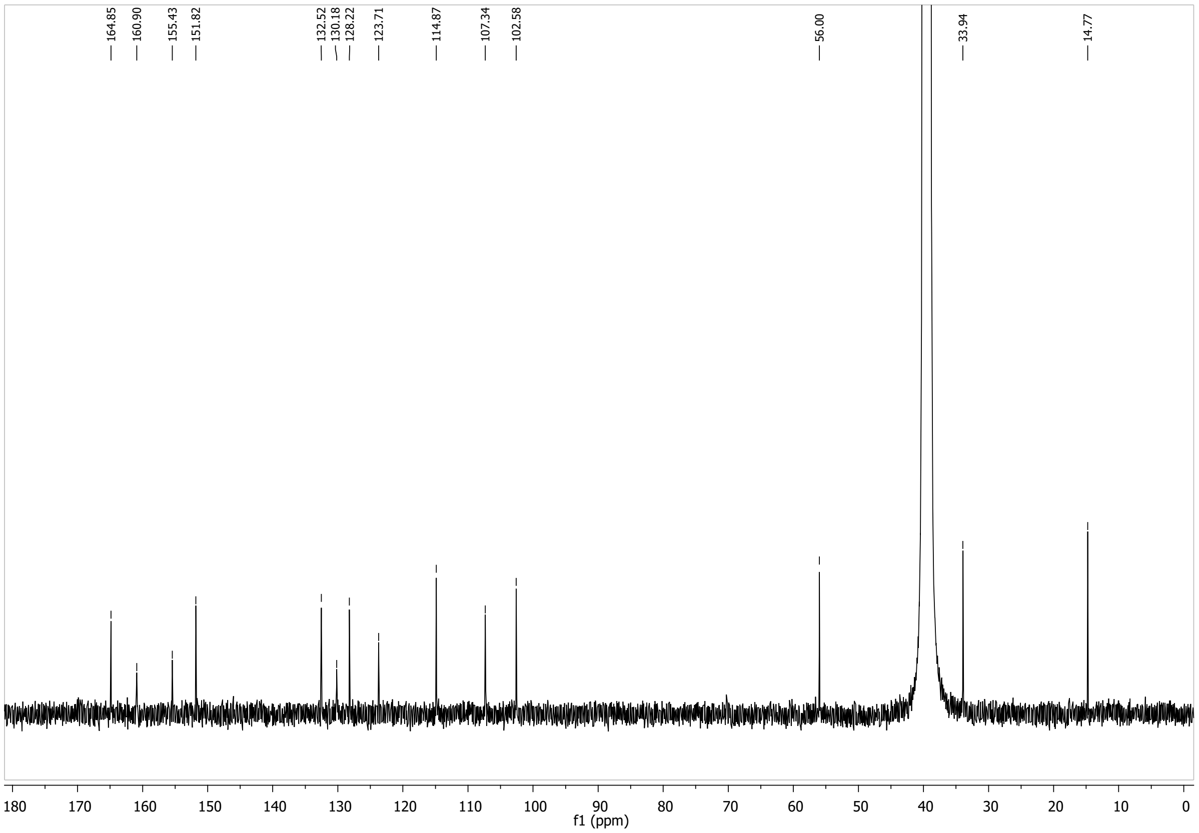

**Figure S70.** $^{13}$C-NMR spectrum of **3** (101 MHz, DMSO-$d_6$).

## 11. Mass Spectra

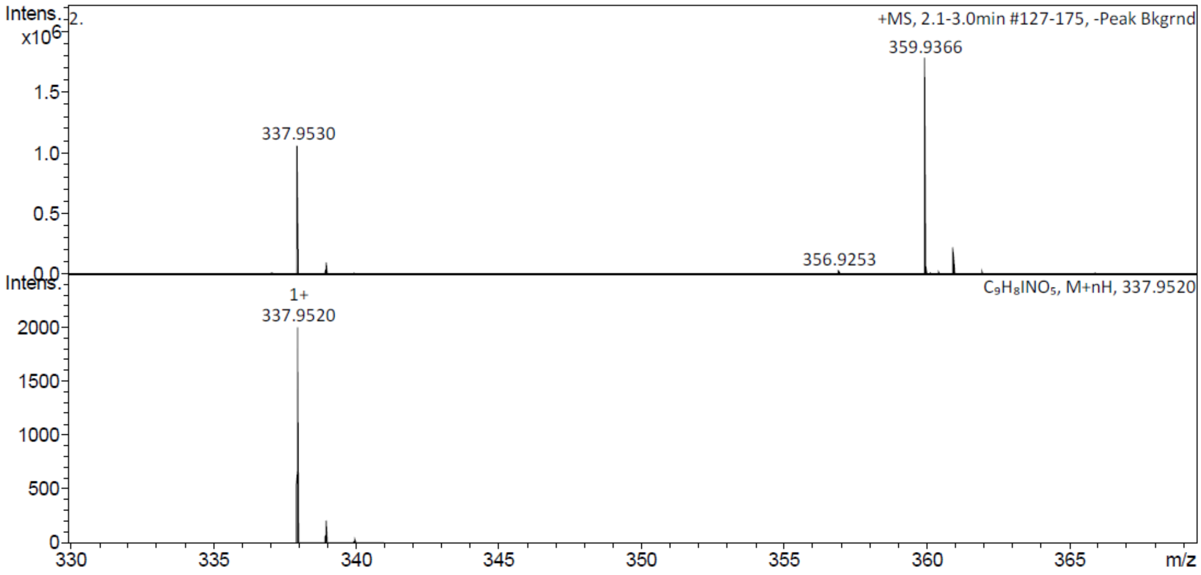


**Figure S71.** HR-ESI mass spectrum of **B** (positive ion mode, MeOH) and predicted mass spectrum of peaks which belongs to **B.**

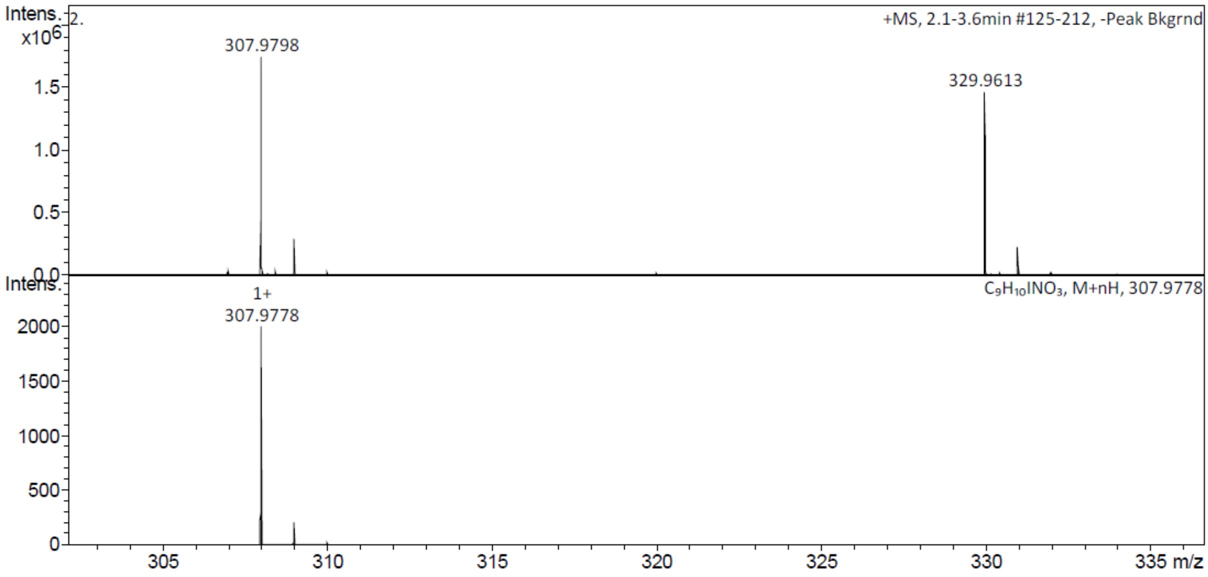


**Figure S72.** HR-ESI mass spectrum of **C** (positive ion mode, MeOH) and predicted mass spectrum of peaks which belongs to **C.**

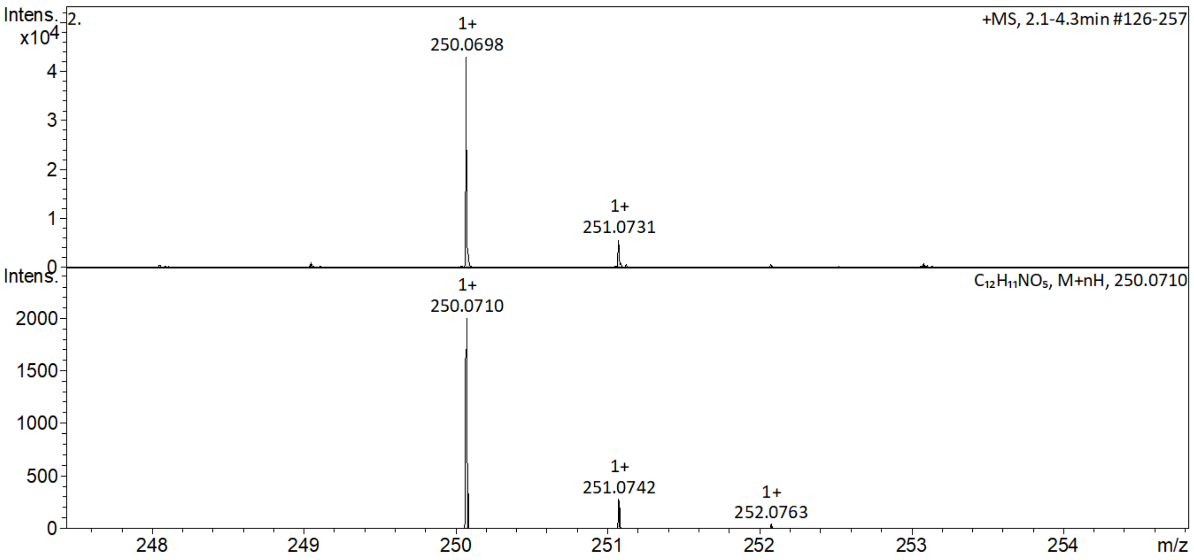


**Figure S73.** HR-ESI mass spectrum of **D** (positive ion mode, MeOH) and predicted mass spectrum of peaks which belongs to **D.**

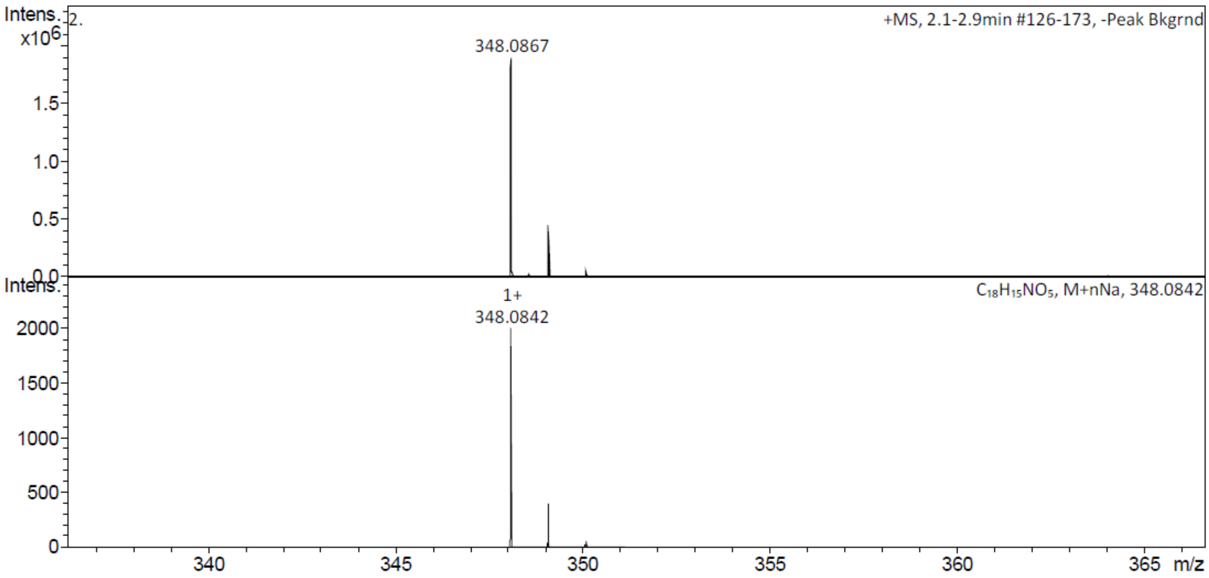


**Figure S74.** HR-ESI mass spectrum of **E** (positive ion mode, MeOH) and predicted mass spectrum of peaks which belongs to **E.**

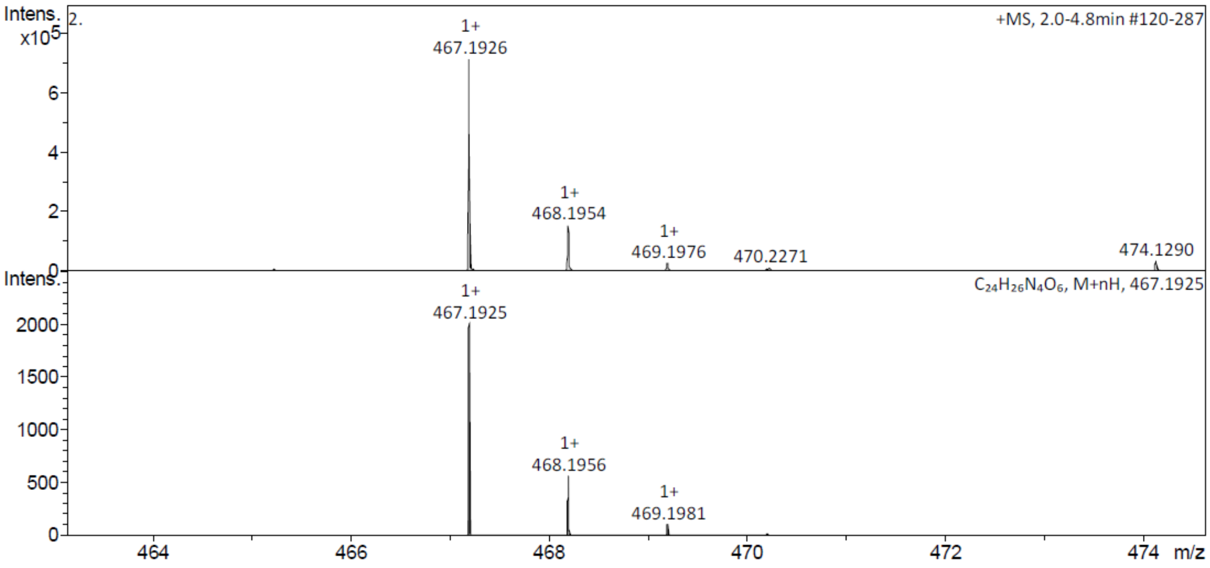


**Figure S75.** HR-ESI mass spectrum of **G** (positive ion mode, MeOH) and predicted mass spectrum of peaks which belongs to **G.**

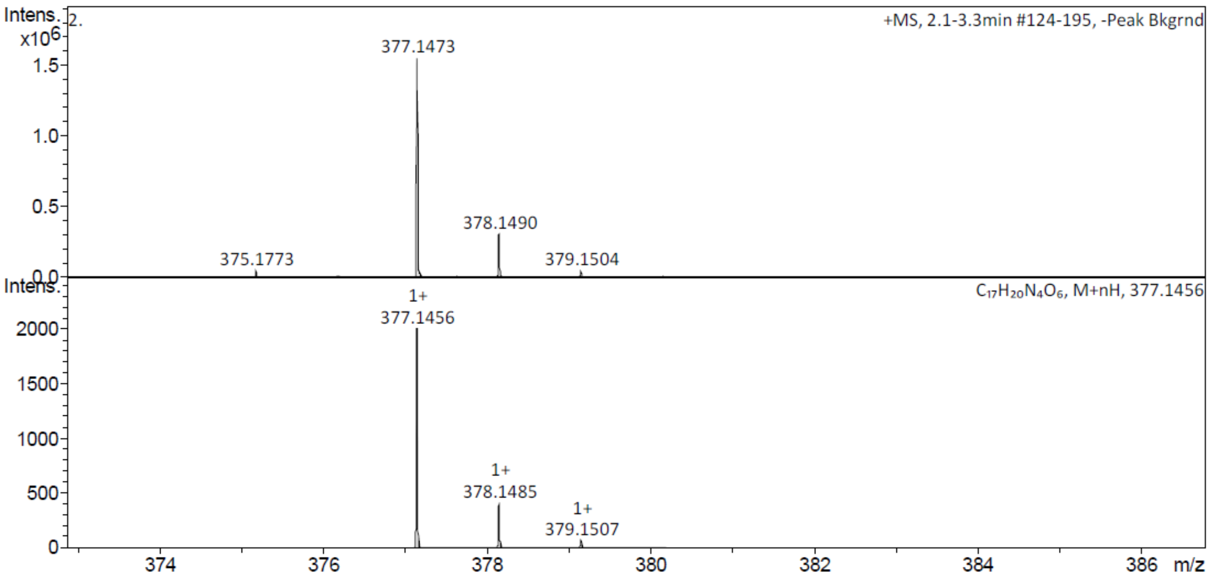


**Figure S76.** HR-ESI mass spectrum of **H** (positive ion mode, MeOH) and predicted mass spectrum of peaks which belongs to **H.**

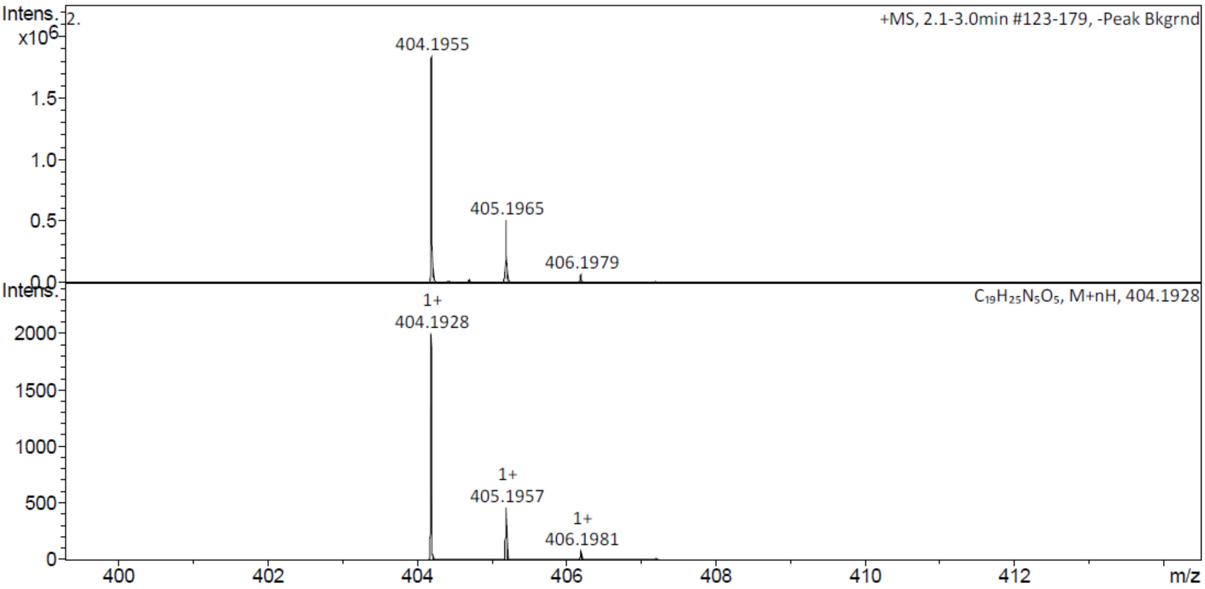


**Figure S77.** HR-ESI mass spectrum of **I** (positive ion mode, MeOH) and predicted mass spectrum of peaks which belongs to **I.**

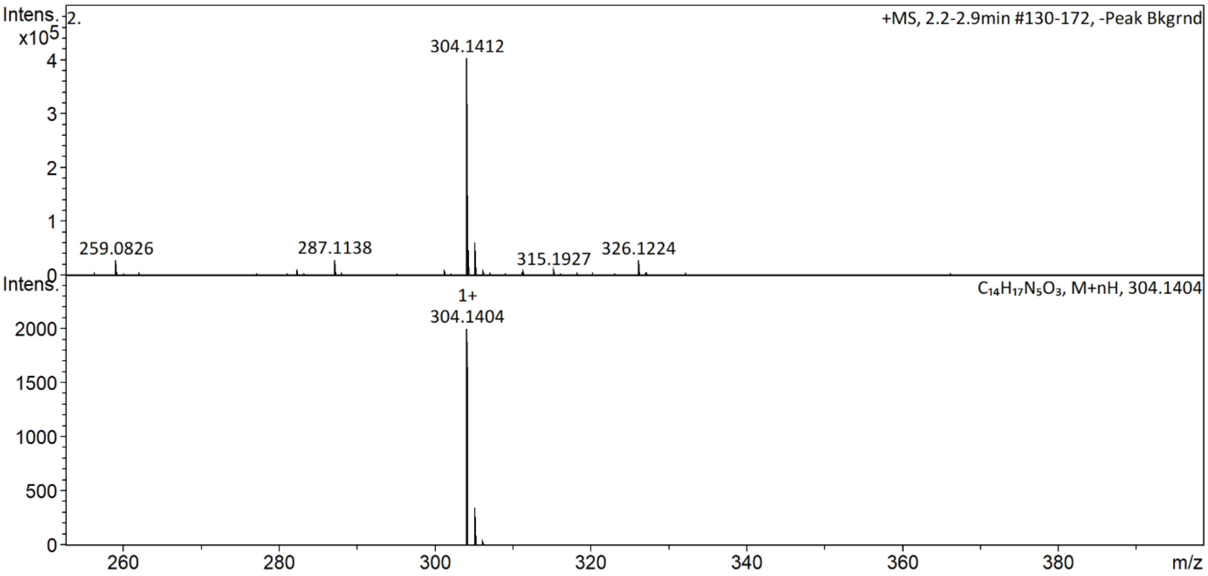


**Figure S78.** HR-ESI mass spectrum of **3** (positive ion mode, MeOH) and predicted mass spectrum of peaks which belongs to **3.**

## 12. Analytical HPLC

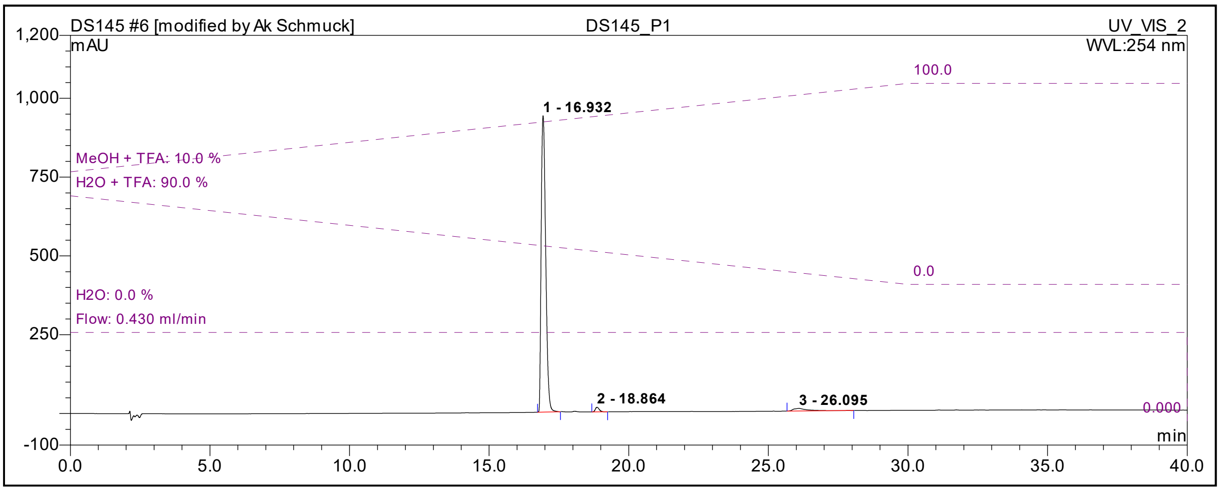


**Figure S79.** Analytical HPLC (RP 18 MeOH/H2O + 0.1% TFA, 10% MeOH + 0.1% TFA to 100% MeOH + 0.1% TFA, gradient) of compound **3**.
