# Supplementary material for: Evolution of Artificial Arginine Analogues—Fluorescent Guanidiniocarbonyl-Indoles as Efficient Oxo-Anion Binders
Source: Molecules. 2022 May 7;27(9):3005. doi: 10.3390/molecules27093005 (PMC9104999; doi:10.3390/molecules27093005)

checkCIF/PLATON report

You have not supplied any structure factors. As a result the full set of tests cannot be run.

THIS REPORT IS FOR GUIDANCE ONLY. IF USED AS PART OF A REVIEW PROCEDURE FOR PUBLICATION, IT SHOULD NOT REPLACE THE EXPERTISE OF AN EXPERIENCED CRYSTALLOGRAPHIC REFEREE.

No syntax errors found. CIF dictionary Interpreting this report

Datablock: ds_k1m

```
Bond precision:   C-C = 0.0065 A          Wavelength=1.54178

Cell:             a=20.149(2)    b=6.3428(7)         c=13.6572(14)
                  alpha=90       beta=122.048(6)     gamma=90
Temperature:      100 K

                 Calculated                     Reported
Volume           1479.4(3)                      1479.4(3)
Space group      C 2                            C 2
Hall group       C 2y                           C 2y
Moiety formula   C13 H15 N5 O2, 2(H2 O)         ?
Sum formula      C13 H19 N5 O4                  C13 H19 N5 O4
Mr               309.33                         309.33
Dx,g cm-3        1.389                          1.389
Z                4                              4
Mu (mm-1)        0.882                          0.882
F000             656.0                          656.0
F000'            658.19
h,k,lmax         24,7,16                        24,7,16
Nref             2731[ 1499]                    2636
Tmin,Tmax        0.967,0.972                    0.500,0.750
Tmin'            0.959

Correction method= # Reported T Limits: Tmin=0.500 Tmax=0.750
AbsCorr = MULTI-SCAN

Data completeness= 1.76/0.97        Theta(max)= 68.548

R(reflections)= 0.0468( 2301)                          wR2(reflections)=
                                                       0.1375( 2636)
S = 0.955                 Npar= 241
```

The following ALERTS were generated. Each ALERT has the format
test-name_ALERT_alert-type_alert-level.
Click on the hyperlinks for more details of the test.

Alert level C

```
STRVA01_ALERT_4_C          Flack test results are ambiguous.
           From the CIF: _refine_ls_abs_structure_Flack    0.400
           From the CIF: _refine_ls_abs_structure_Flack_su    0.300
PLAT085_ALERT_2_C SHELXL Default Weighting Scheme is not Optimized     Please Check
PLAT089_ALERT_3_C Poor Data / Parameter Ratio (Zmax < 18) ........       6.22 Note
PLAT222_ALERT_3_C NonSolvent Resd 1  H   Uiso(max)/Uiso(min) Range        4.7 Ratio
PLAT250_ALERT_2_C Large U3/U1 Ratio for Average U(i,j) Tensor ....        2.2 Note
PLAT340_ALERT_3_C Low Bond Precision on  C-C Bonds ..............    0.00645 Ang.
PLAT417_ALERT_2_C Short Inter D-H..H-D       H4A      ..H8       .       2.12 Ang.
                                               2-x,1+y,2-z  =      2_767 Check
```

Alert level G

```
PLAT002_ALERT_2_G Number of Distance or Angle Restraints on AtSite          7 Note
PLAT032_ALERT_4_G Std. Uncertainty on Flack Parameter Value High .      0.300 Report
PLAT128_ALERT_4_G Alternate Setting for Input Space Group  C2            I2 Note
PLAT176_ALERT_4_G The CIF-Embedded .res File Contains SADI Records          1 Report
PLAT790_ALERT_4_G Centre of Gravity not Within Unit Cell: Resd.  #          3 Note
              H2 O
PLAT790_ALERT_4_G Centre of Gravity not Within Unit Cell: Resd.  #          4 Note
              H2 O
PLAT860_ALERT_3_G Number of Least-Squares Restraints ............          7 Note
PLAT965_ALERT_2_G The SHELXL WEIGHT Optimisation has not Converged     Please Check
```

0 **ALERT level A** = Most likely a serious problem - resolve or explain
0 **ALERT level B** = A potentially serious problem, consider carefully
7 **ALERT level C** = Check. Ensure it is not caused by an omission or oversight
8 **ALERT level G** = General information/check it is not something unexpected

0 ALERT type 1 CIF construction/syntax error, inconsistent or missing data
5 ALERT type 2 Indicator that the structure model may be wrong or deficient
4 ALERT type 3 Indicator that the structure quality may be low
6 ALERT type 4 Improvement, methodology, query or suggestion
0 ALERT type 5 Informative message, check

Datablock: ds_k2m

Bond precision:	C-C = 0.0040 A		Wavelength=1.54178
Cell:	a=5.0421(4)	b=15.7408(14)	c=17.5287(15)
	alpha=114.599(3)	beta=94.776(3)	gamma=95.974(3)
Temperature:	100 K		

	Calculated	Reported
Volume	1245.68(19)	1245.68(18)
Space group	P -1	P -1
Hall group	-P 1	-P 1
Moiety formula	2(C9 H14 N5 O2), 2(Cl), H2 O, 0.66(O)	?
Sum formula	C18 H30 Cl2 N10 O5.66	C9 H15.66 Cl N5 O2.83
Mr	547.98	274.65
Dx,g cm-3	1.461	1.464
Z	2	4
Mu (mm-1)	2.821	2.821
F000	574.6	577.0
F000'	577.71	
h,k,lmax	6,20,22	6,20,22
Nref	5515	5437
Tmin,Tmax	0.702,0.832	0.580,0.750
Tmin'	0.463	

Correction method= # Reported T Limits: Tmin=0.580 Tmax=0.750
AbsCorr = MULTI-SCAN

Data completeness= 0.986 Theta(max)= 81.241

R(reflections)= 0.0512(5275) wR2(reflections)= 0.1446(5437)

S = 1.137 Npar= 400

The following ALERTS were generated. Each ALERT has the format
test-name_ALERT_alert-type_alert-level.
Click on the hyperlinks for more details of the test.

Alert level C

```
PLAT041_ALERT_1_C Calc. and Reported SumFormula    Strings  Differ     Please Check
PLAT043_ALERT_1_C Calculated and Reported Mol. Weight Differ by ..       1.32 Check
PLAT077_ALERT_4_C Unitcell Contains Non-integer Number of Atoms ..     Please Check
PLAT193_ALERT_1_C Cell and Diffraction Temperatures Differ by ....          3 Degree
PLAT222_ALERT_3_C NonSolvent Resd 1  H   Uiso(max)/Uiso(min) Range        6.8 Ratio
PLAT352_ALERT_3_C Short  N-H (X0.87,N1.01A)  N3_1     - H3A_1    .       0.72 Ang.
PLAT352_ALERT_3_C Short  N-H (X0.87,N1.01A)  N5_1     - H5_1     .       0.75 Ang.
```

Alert level G

```
FORMU01_ALERT_2_G  There is a discrepancy between the atom counts in the
            _chemical_formula_sum and the formula from the _atom_site* data.
            Atom count from _chemical_formula_sum:C9 H15.66 Cl1 N5 O2.83
            Atom count from the _atom_site data:  C9 H15 Cl1 N5 O2.83
CELLZ01_ALERT_1_G Difference between formula and atom_site contents detected.
```

```
CELLZ01_ALERT_1_G WARNING: H atoms missing from atom site list. Is this intentional?
           From the CIF: _cell_formula_units_Z    4
           From the CIF: _chemical_formula_sum  C9 H15.66 Cl N5 O2.83
           TEST: Compare cell contents of formula and atom_site data

           atom    Z*formula  cif sites diff
           C         36.00     36.00    0.00
           H         62.64     60.00    2.64
           Cl         4.00      4.00    0.00
           N         20.00     20.00    0.00
           O         11.32     11.32    0.00
PLAT002_ALERT_2_G Number of Distance or Angle Restraints on AtSite          3 Note
PLAT003_ALERT_2_G Number of Uiso or Uij Restrained non-H Atoms ...          2 Report
PLAT045_ALERT_1_G Calculated and Reported Z Differ by a Factor ...     0.5000 Check
PLAT068_ALERT_1_G Reported F000 Differs from Calcd (or Missing)...     Please Check
PLAT154_ALERT_1_G The s.u.'s on the Cell Angles are Equal ..(Note)      0.003 Degree
PLAT172_ALERT_4_G The CIF-Embedded .res File Contains DFIX Records          2 Report
PLAT178_ALERT_4_G The CIF-Embedded .res File Contains SIMU Records          1 Report
PLAT300_ALERT_4_G Atom Site Occupancy of O1_4       Constrained at       0.38 Check
PLAT300_ALERT_4_G Atom Site Occupancy of O1_5       Constrained at       0.28 Check
PLAT302_ALERT_4_G Anion/Solvent/Minor-Residue Disorder (Resd  6  )       100% Note
PLAT302_ALERT_4_G Anion/Solvent/Minor-Residue Disorder (Resd  7  )       100% Note
PLAT304_ALERT_4_G Non-Integer Number of Atoms in ..... (Resd  6  )       0.38 Check
PLAT304_ALERT_4_G Non-Integer Number of Atoms in ..... (Resd  7  )       0.28 Check
PLAT311_ALERT_2_G Isolated Disordered Oxygen Atom (No H's ?) .....       O1_4 Check
PLAT311_ALERT_2_G Isolated Disordered Oxygen Atom (No H's ?) .....       O1_5 Check
PLAT431_ALERT_2_G Short Inter HL..A Contact  Cl2      ..O1_4     .       3.06 Ang.
                                                    1+x,y,z  =      1_655 Check
PLAT720_ALERT_4_G Number of Unusual/Non-Standard Labels ..........        65 Note
PLAT790_ALERT_4_G Centre of Gravity not Within Unit Cell: Resd.  #          3 Note
              Cl
PLAT790_ALERT_4_G Centre of Gravity not Within Unit Cell: Resd.  #          5 Note
              H2 O
PLAT860_ALERT_3_G Number of Least-Squares Restraints ............          9 Note
PLAT933_ALERT_2_G Number of HKL-OMIT Records in Embedded .res File          1 Note
```

---

0 **ALERT level A** = Most likely a serious problem - resolve or explain
0 **ALERT level B** = A potentially serious problem, consider carefully
7 **ALERT level C** = Check. Ensure it is not caused by an omission or oversight
24 **ALERT level G** = General information/check it is not something unexpected

8 ALERT type 1 CIF construction/syntax error, inconsistent or missing data
7 ALERT type 2 Indicator that the structure model may be wrong or deficient
4 ALERT type 3 Indicator that the structure quality may be low
12 ALERT type 4 Improvement, methodology, query or suggestion
0 ALERT type 5 Informative message, check

It is advisable to attempt to resolve as many as possible of the alerts in all categories. Often the minor alerts point to easily fixed oversights, errors and omissions in your CIF or refinement strategy, so attention to these fine details can be worthwhile. In order to resolve some of the more serious problems it may be necessary to carry out additional measurements or structure refinements. However, the purpose of your study may justify the reported deviations and the more serious of these should normally be commented upon in the discussion or experimental section of a paper or in the "special_details" fields of the CIF. checkCIF was carefully designed to identify outliers and unusual parameters, but every test has its limitations and alerts that are not important in a particular case may appear. Conversely, the absence of alerts does not guarantee there are no aspects of the results needing attention. It is up to the individual to critically assess their own results and, if necessary, seek expert advice.

Publication of your CIF in IUCr journals

A basic structural check has been run on your CIF. These basic checks will be run on all CIFs submitted for publication in IUCr journals (*Acta Crystallographica*, *Journal of Applied Crystallography*, *Journal of Synchrotron Radiation*); however, if you intend to submit to *Acta Crystallographica Section C* or *E* or *IUCrData*, you should make sure that full publication checks are run on the final version of your CIF prior to submission.

Publication of your CIF in other journals

Please refer to the *Notes for Authors* of the relevant journal for any special instructions relating to CIF submission.

PLATON version of 20/01/2022; check.def file version of 19/01/2022

Datablock ds_k1m - ellipsoid plot

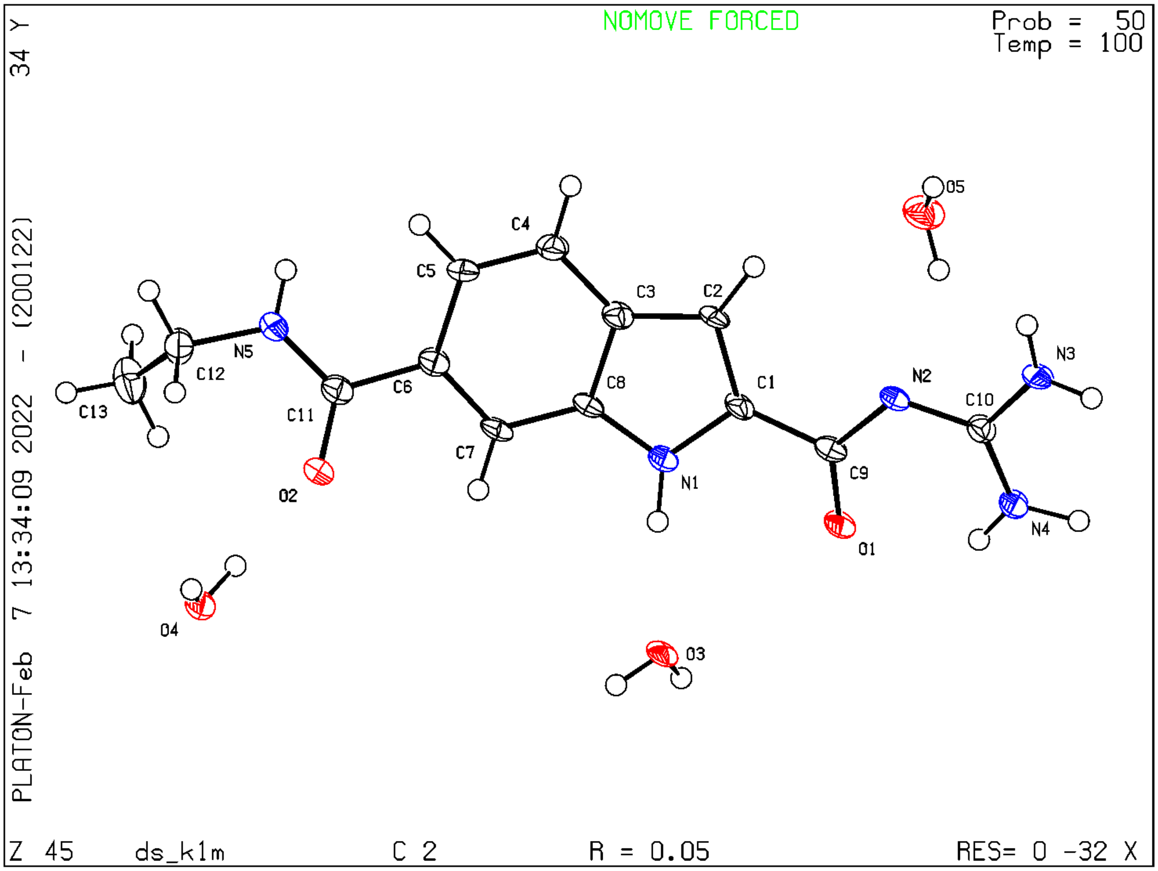

Datablock ds_k2m - ellipsoid plot

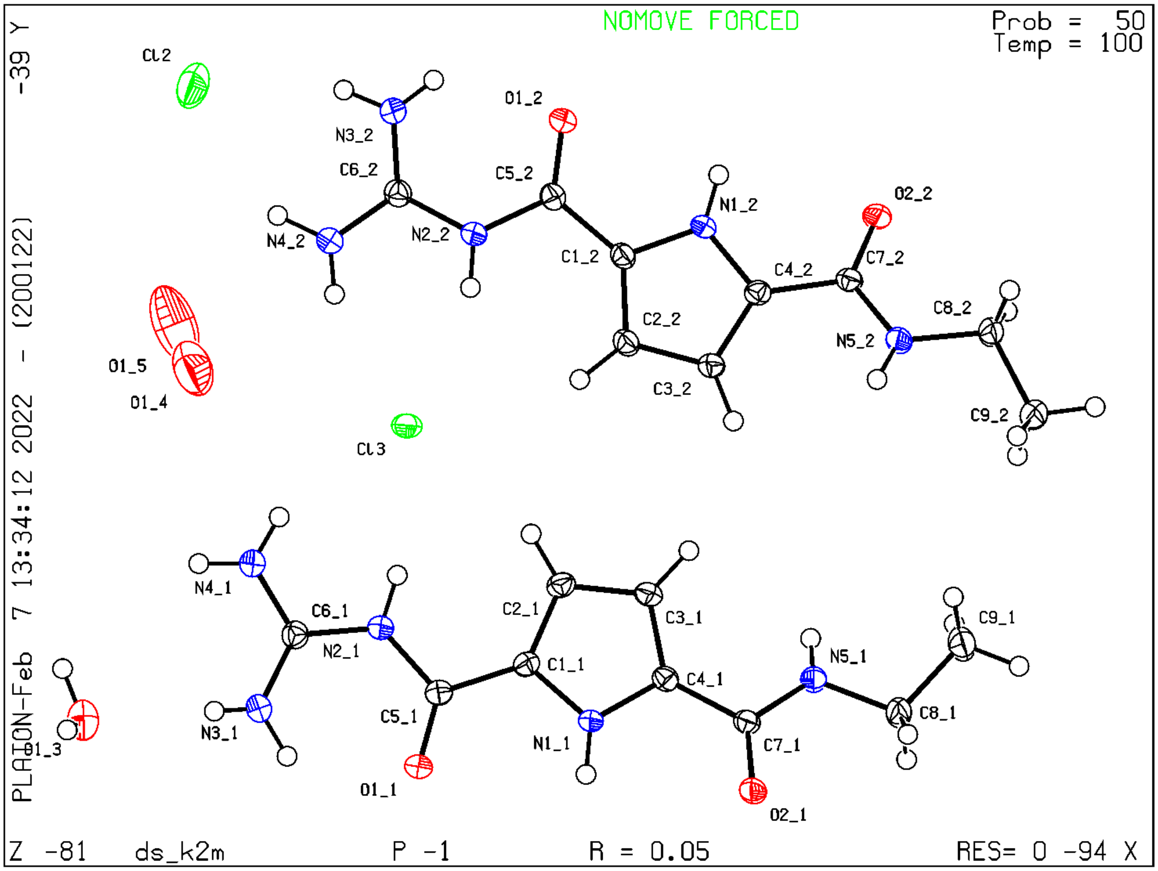

Supplement: Supplementary file 1 [file molecules-27-03005-s001.zip › X-ray files_Molecules/checkcif_(1) and (2).pdf]
